# A Multi‐Stimuli Transformational Network of Benzil‐Based Pd(II) Assemblies

**DOI:** 10.1002/anie.9050553

**Published:** 2026-07-09

**Authors:** Zhiwei Zeng, A. Priscila Gia, Alexander S. Mikherdov, Mert Acar, Björn Schmidt, Guido H. Clever

**Affiliations:** ^1^ Department of Chemistry and Chemical Biology TU Dortmund University Dortmund Germany; ^2^ School of Chemistry and Chemical Engineering Nanjing University of Science and Technology Nanjing China; ^3^ Department of Organic Chemistry Nanostructured Molecular Systems and Materials Group Universidad Autónoma de Madrid Madrid Spain

**Keywords:** chemical networks, coordination cages, post‐assembly modification, self‐assembly, supramolecular chemistry

## Abstract

Achieving precise control over supramolecular topology and nuclearity in coordination‐driven self‐assemblies through integration of multiple stimuli remains a challenge. We here introduce ligand **L^A^**, based on a flexible and reactive 1,2‐dicarbonyl benzil‐based backbone, able to adopt several conformations. A range of homo‐ and heteroleptic Pd(II) assemblies is formed with different nuclearities and topologies, connected through distinct transformation pathways. In homoleptic systems, folded and open conformations of **L^A^** afford the mononuclear complex Pd**L^A^**_2_ and the lantern‐shaped cage Pd_2_ **L^A^**_4_. Incorporation of secondary ligands **L^C^** or **L^D^** induces more expanded **L^A^** conformations, yielding *cis*‐Pd_2_ **L^A^**_2_ **L^C^**_2_ or a rare isosceles triangular Pd_3_ **L^A^**_2_ **L^D^**_4_ ring. These heteroleptic architectures can be interconverted, and both can undergo guest‐induced retro‐cage‐to‐cage transformation to regenerate homoleptic species. Furthermore, **L^A^** can also be conformationally locked through condensation of its benzil backbone with 1,2‐phenylenediamine, producing the rigid quinoxaline ligand **L^B^**. This transformation can occur in a post‐assembly fashion, converting both homoleptic and heteroleptic **L^A^**‐based structures into the Pd**L^B^**_2_ complex. The conformation and accessibility of **L^A^** reactive sites within the architectures, as well as the nature of the encapsulated guest, strongly influence assembly stability and reactivity in this condensation, reminiscent of how nature controls functional group reactivity through effects of nanoscopic confinement, conformational restriction, and allosteric regulation.

## Graphical abstract

Incorporation of a flexible and chemically reactive benzil backbone into the ligand structure allows it to access multiple conformations, enabling the formation of diverse homo‐ and heteroleptic Pd(II) assemblies with different nuclearities and topologies. This conformational versatility also supports multiple stimuli‐induced transformations driven by guest binding, secondary ligand incorporation, or condensation with phenylenediamine.

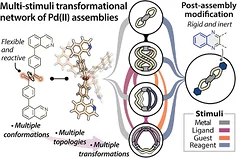

## 1 Introduction

**FIGURE 1:**
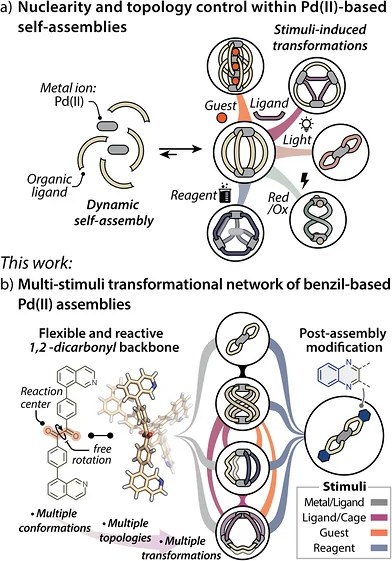
(a) Stimuli‐induced nuclearity and topology transformations of Pd(II) coordination cages. (b) Flexible ligand **L^A^**, investigated in this work, featuring a 1,2‐dicarbonyl benzil backbone that enables the formation of multiple Pd(II) assemblies with variable nuclearity, prone to multi‐stimuli‐responsive transformations.

Due to their intrinsic flexibility, biomolecular systems based on proteins, RNA, and DNA can undergo changes in shape and size in response to external stimuli, enabling them to adapt to dynamic environments, modulate their functions, and participate in multistate regulatory networks [1, 2, 3, 4]. Inspired by these natural systems, synthetic supramolecular architectures have been designed to emulate such adaptive, shape‐shifting behavior. Among them, metallosupramolecular self‐assemblies such as metallomacrocycles and coordination cages occupy a prominent position, as the dynamic nature of coordination bonds allows for reversible structural reorganization under various external stimuli (Figure 1a) [5, 6]. This responsiveness enables such artificial constructs to mimic biological processes, including allosteric binding [7], molecular recognition [8] and transport [9], enzyme‐like catalysis [10, 11], and multi‐state transformational networks [12].

One notable manifestation of size‐ and shape‐shifting behavior in metallosupramolecular architectures is a change in nuclearity and topology, that is, the number of metal atoms within an assembly and their connectivity via bridging organic ligands [13]. Many Pd(II) coordination cages are capable of altering their nuclearity/topology in response to external factors such as a change in component ratio, solvent, concentration, or temperature [14, 15, 16, 17, 18]. It was shown that the geometry of the organic ligand plays a crucial role in determining the size and shape of the resulting assembly [13, 19, 20]. While ligand conformational flexibility often enables structural adaptivity of supramolecular structure formation [21, 22, 23, 24] and facilitates interconversion between species of different nuclearities and topologies, it may also complicate the isolation of discrete assemblies [25, 26, 27, 28, 29]. In such cases, guest inclusion can be exploited to template architectures of specific dimensions or connectivity [30, 31, 32, 33]. In some instances, guest binding or exchange can even trigger interconversion between distinct assemblies [34], for example, in the reversible restructuring of azulene‐based lantern‐shaped Pd_2_L_4_ cages to Pd_4_L_8_ tetrahedra reported by us [35], or the ion‐dependent reversible interlocking of Pd_2_L_4_ cages to give Pd_4_L_8_ catenanes, first reported by Kuroda [36].

Furthermore, homoleptic cages (containing only one type of organic ligand) may undergo changes in size and nuclearity upon introduction of a second kind of ligand or through cage‐to‐cage transformations under consumption of another assembly [37]. The formation of heteroleptic structures by merging of homoleptic species can give rise to nuclearities and structural motifs that are inaccessible in assemblies based on only one kind of ligand [38, 39, 40]. Consequently, this approach has recently opened access to a wide range of new supramolecular architectures of low symmetry and unique topology [23, 41, 42, 43, 44, 45, 46].

Incorporation of addressable functional moieties such as photoswitchable [47], redox [48], or reactive [49] units into ligand backbones can provide additional control over assembly size and shape by inducing light‐, electron‐, or reagent‐driven changes in ligand geometry. For instance, the introduction of azo(het)aryl functionalities into lantern‐shaped Pd_2_L_4_ coordination cages enables their conversion, via ligand *E–Z* photoisomerization, into assemblies of different nuclearities, e.g. PdL_2_ as shown by Beves [50, 51] or Pd_3_L_6_ reported by von Krbek [52]. We introduced Pd(II) systems based on photoactive dithienylethene ligands, in which photoswitching converts Pd_3_L_6_ rings into Pd_24_L_48_ nanospheres [53], while a Pd_2_L_4_ lantern‐shaped cage can be transformed into a Pd_2_L_3_(solv)_2_ bowl [54]. Recently, Goeb and our group reported a rare case of redox‐induced topology switching between a Pd_2_L_4_ cage and a homoleptic Pd_2_L_2_(solv)_4_ macrocycle or a heteroleptic Pd_2_L_2_L′(solv)_2_ bowl using a redox‐active, π‐extended tetrathiafulvalene (exTTF)‐based ligand [55]. Examples of nuclearity and topology switching achieved through covalent post‐assembly modification also remain rare, owing to the limited number of chemical transformations capable of altering ligand geometry while preserving the integrity of the metallosupramolecular assembly [49, 56, 57]. To the best of our knowledge, concerning topology/nuclearity switching in Pd(II) systems, the only reported example was described by Pilgrim and co‐workers, who utilized an inverse electron‐demand Diels–Alder reaction on tetrazine‐based ligand backbones, followed by oxidation, to interconvert between Pd_4_L_8_ tetrahedra and Pd_2_L_4_ lantern‐shaped cages [58].

While the action of an isolated individual stimulus such as the addition of a ligand, guest, or chemical reagent or the irradiation with light has been reported to control the nuclearity and topology of Pd(II) supramolecular assemblies, the combination of several distinct stimuli within a single, interconnected transformation network remains challenging [12]. Herein, we introduce ligand **L^A^**, which contains a flexible and chemically reactive 1,2‐dicarbonyl benzil‐based backbone (Figure 1b). Unrestricted rotation around the central carbon–carbon bond allows **L^A^** to adopt several conformations, making it suitable for incorporation into a variety of homo‐ and heteroleptic Pd(II) assemblies with different nuclearities and topologies. Its flexibility allows it to progress between different conformational states in response to external stimuli, such as the addition of a guest or secondary ligand, thus inducing the formation of specific metallosupramolecular structures. In addition, the 1,2‐dicarbonyl group can undergo condensation with diamines, for example, 1,2‐phenylenediamine, to generate a rigid quinoxaline backbone, further altering ligand geometry. This reactivity provides an additional chemical stimulus for the post‐assembly transformation into new species. Taken together, the combined action of three different types of stimuli (ligand, guest, and reagent) allowed us to establish a multistate transformational network that connects a range of homoleptic and heteroleptic Pd(II) assemblies of different sizes and shapes.

## 2 Results and Discussion

### 2.1 Homoleptic Assembly Formation

**SCHEME 1:**
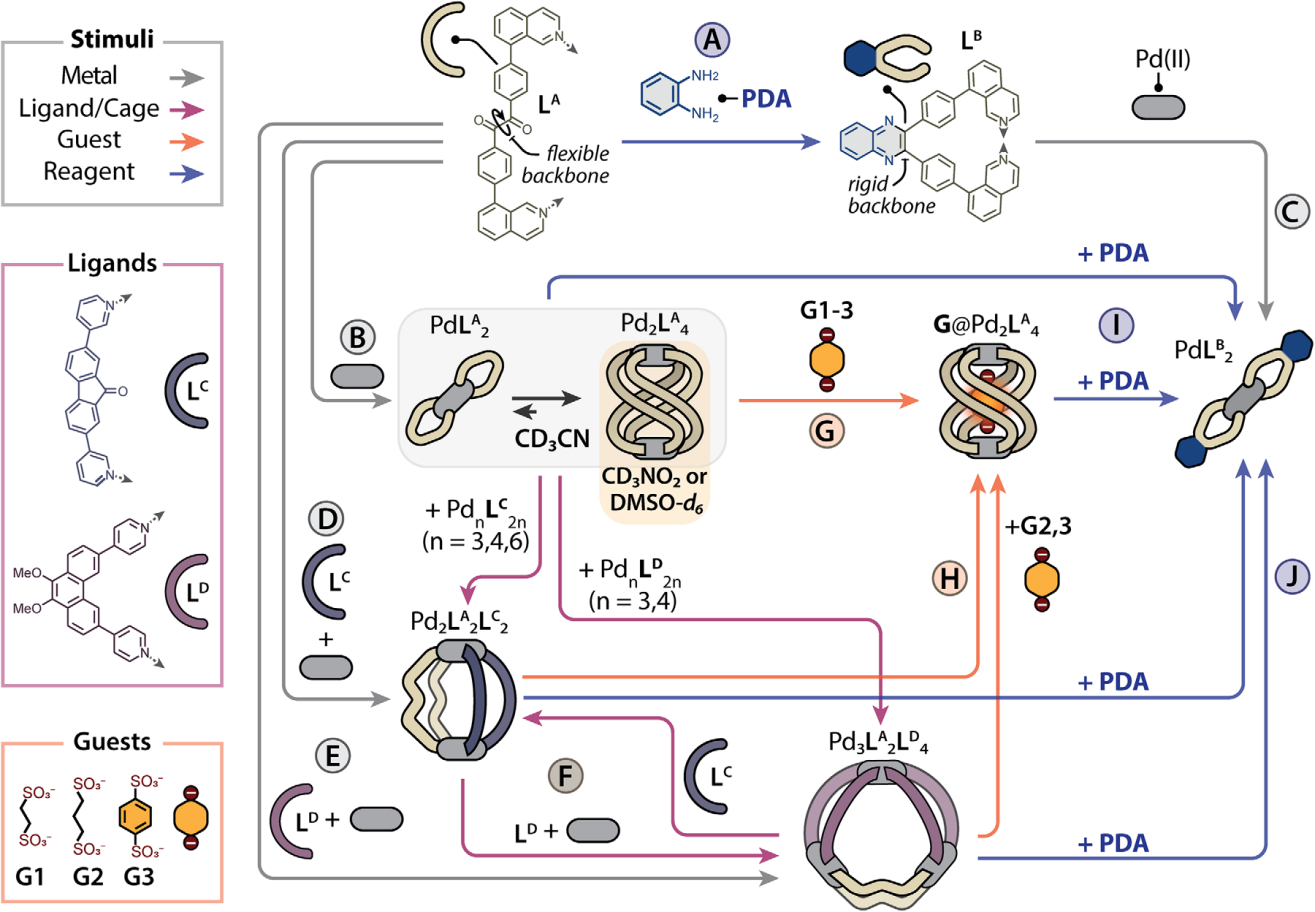
Transformations of ligand **L^A^** and its multiple Pd(II)‐based self‐assemblies. (A) Condensation of **L^A^** with 1,2‐phenylenediamine (**PDA**); self‐assembly of Pd_2_ **L^A^**_4_/Pd**L^A^**_2_ (B), Pd**L^B^**_2_ (C), Pd_2_ **L^A^**_2_ **L^C^**_2_ (D), and Pd_3_ **L^A^**_2_ **L^D^**_4_ (E) species; (F) interconversion of Pd_2_ **L^A^**_2_ **L^C^**_2_ and Pd_3_ **L^A^**_2_ **L^D^**_4_ heteroleptic assemblies; (G) guest encapsulation in cage Pd_2_ **L^A^**_4_; (H) guest‐induced transformation of Pd_2_ **L^A^**_2_ **L^C^**_2_ and Pd_3_ **L^A^**_2_ **L^D^**_4_ heteroleptic assemblies into **G**@Pd_2_ **L^A^**_4_; post‐assembly condensation of homoleptic (I) and heteroleptic (J) Pd(II) species with **PDA**.

Benzil‐based ligand **L^A^** with isoquinoline donor groups can react with 1,2‐phenylenediamine (**PDA**) under the transformation of its flexible 1,2‐dicarbonyl backbone into a rigid quinoxaline moiety in the resulting ligand **L^B^** (Scheme 1A) [59]. This transformation acts as a conformational lock and results in a drastic change in ligand shape, affecting its mode of interaction with metal cations. While flexible **L^A^** has a hinge‐like shape, allowing it to adopt several conformations suitable for forming various assembly topologies, rigid derivative **L^B^** has a pincer‐like shape, more favorable for metal chelation.

**FIGURE 2:**
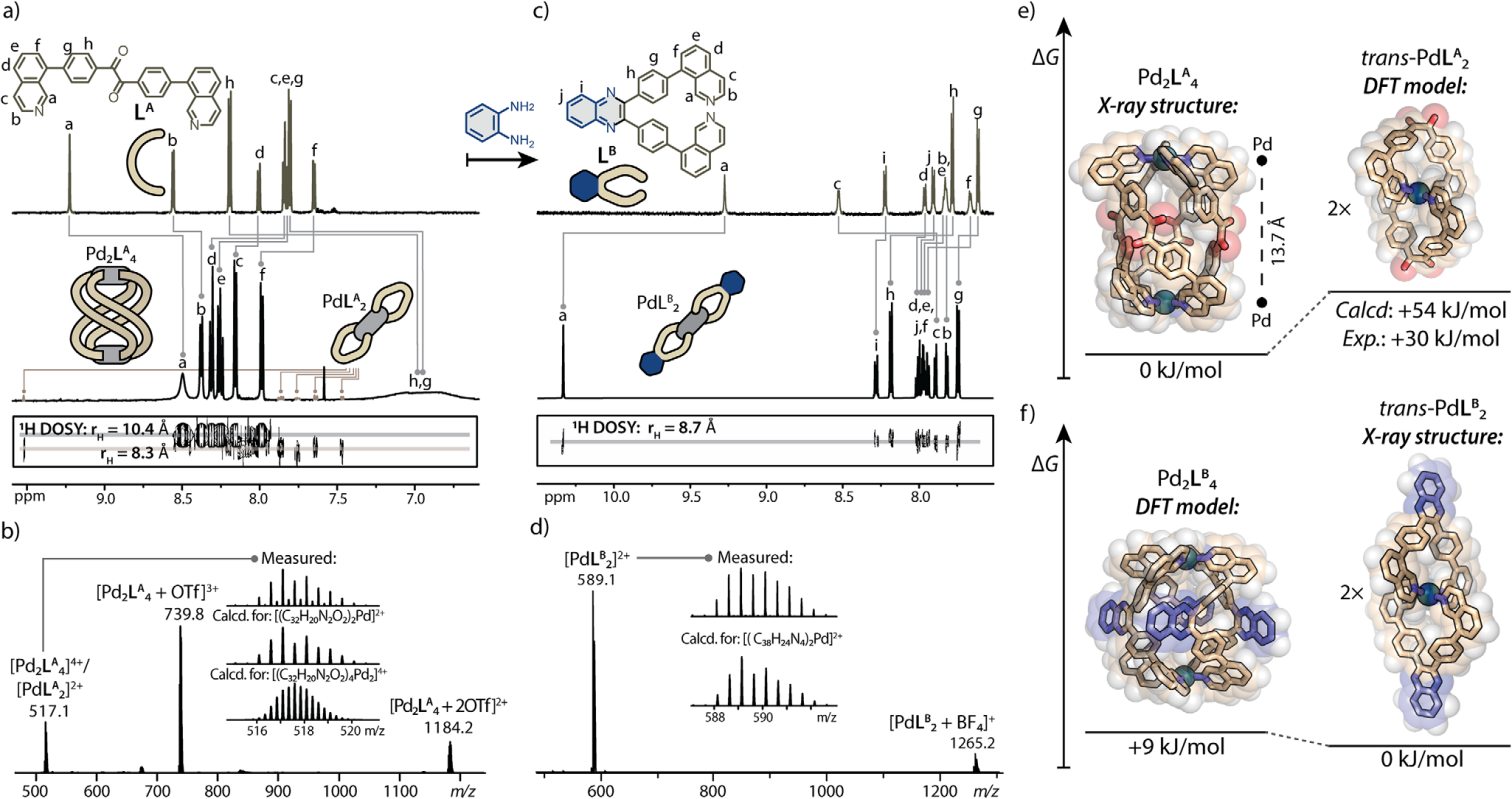
(a) ^1^H NMR spectra (CD_3_CN) of **L^A^** (top) and the Pd_2_ **L^A^**_4_/Pd**L^A^**_2_ mixture (with OTf^–^ counter‐ions; bottom) with DOSY traces for Pd_2_ **L^A^**_4_ and Pd**L^A^**_2_; (b) ESI mass spectrum of the Pd_2_ **L^A^**_4_/Pd**L^A^**_2_ mixture with OTf^–^ counter‐ions; (c) ^1^H NMR spectra (CD_3_CN) of **L^B^** (top) and Pd**L^B^**_2_ (bottom) with DOSY trace for Pd**L^B^**_2_; (d) ESI mass spectrum of Pd**L^B^**_2_; (e, f) X‐ray structures (Pd_2_ **L^A^**_4_ and Pd**L^B^**_2_), DFT models (Pd**L^A^**_2_ and Pd_2_ **L^B^**_4_) and calculated free energy differences between corresponding cages and mononuclear complexes.

The reaction of ligand **L^A^** (2 eq.) with [Pd(CH_3_CN)_4_](X)_2_ (1.1 eq.) (X = BF_4_^–^ or OTf^–^) in CD_3_CN at room temperature (RT) almost immediately produced two species distinct from the initial ligand (Scheme 1B). Over time, one species gradually converted into the other, indicating the presence of kinetic and thermodynamic products (Figure S17). After 5 days at room temperature (RT), the kinetic product had almost completely disappeared, giving an equilibrium product ratio of approximately 11:1 for the OTf^–^ counterion (Figure 2a). For the assembly with the BF_4_^–^ counterion, the NMR signals of the thermodynamic product were highly broadened at room temperature (complicating the determination of the product ratio) but became noticeably sharper upon heating to 70°C, closely matching the signal positions of the thermodynamic product formed with triflate (Figure S10). These observations suggest that the assembly with BF_4_^–^ leads to a more dynamic structure, whereas the larger and more coordinative OTf^–^ anion more effectively stabilizes a distinct conformation of reduced structural flexibility [60]. In both systems, ESI‐MS analysis revealed ions corresponding to a Pd_2_ **L^A^**_4_ cage and a mononuclear Pd**L^A^**_2_ complex (Figure 2b and Figure S12), consistent with the presence of two products in solution. Additionally, ^1^H DOSY measurements indicated two species with hydrodynamic radii (*r*_H_) of approximately 10 Å for the major thermodynamic product and approximately 8 Å for the minor kinetic one (Table S5), corresponding to larger Pd_2_ **L^A^**_4_ and smaller Pd**L^A^**_2_ structures, respectively (Figure 2a). Notably, the use of alternative solvents such as CD_3_NO_2_ and DMSO‐*d_6_* leads to further stabilization and exclusive formation of the Pd_2_ **L^A^**_4_ cage structure, as confirmed by ESI‐MS (Figure S12) and NMR analyses (Figure S10). Interestingly, in DMSO‐*d_6_*, a twofold splitting of the ligand signals is observed, accompanied by an increase in the hydrodynamic radius to 11.1 Å. A similar behavior was previously observed by us in an acridone‐based Pd(II) helical cage, where such an effect was caused by a solvent‐induced (also from CD_3_CN to DMSO‐*d_6_*) transition from a helicate to a broken‐symmetry mesocate‐like structure [22]. Indeed, DFT optimization of the Pd_2_ **L^A^**_4_ structure starting from a mesocate conformation leads to a “half‐helical” cage geometry, in which one Pd(isoquinoline)_4_ unit is twisted while the other remains untwisted (Figure S58). This geometry is consistent with the observed twofold signal splitting in the NMR spectrum and features an increased Pd–Pd separation (15.1 Å), in agreement with the larger hydrodynamic radius in DSMO‐*d_6_* solution.

Vapor diffusion of diethyl ether into the reaction mixture with BF_4_^–^ counter‐ions in CD_3_CN yielded crystals suitable for X‐ray diffraction, allowing the self‐assembled structure to be determined in the solid state. As expected, the crystal structure corresponds to the Pd_2_ **L^A^**_4_ cage assembly, which adopts a helical shape (Figure 2e). This finding also explains the signal broadening observed in the NMR spectra of Pd_2_ **L^A^**_4_ with BF_4_^–^ counter‐ions, as the helicate cage is likely to equilibrate between structures with opposite twisting senses as well as a mesocate form in solution [22, 61]. In the solid state, cage Pd_2_ **L^A^**_4_ crystallizes in the achiral *P*‐1 space group, and the unit cell contains both *P*‐ and *M*‐helical species, each encapsulating one acetonitrile and one BF_4_^–^ ion in their cavities (Figure S57). The azimuthal angle defined by the N‐Pd vectors of a single ligand in the helical assembly was found to be approximately 135°, while the Pd–Pd separation in the prolate structure is approximately 13.7 Å, thus rather matching the ^1^H DOSY NMR results obtained for the larger (and major) species obtained in solution that seems to additionally associate with outside‐bound anions.

The NMR and ESI‐MS data show that the conformational flexibility of **L^A^** comes with free rotation along the C─C bond in its 1,2‐dicarbonyl unit. Hence, **L^A^** can act as either a bis‐monodentate or a bidentate ligand, leading to the formation of Pd_2_ **L^A^**_4_ cages and Pd**L^A^**_2_ chelates in solution. The equilibrium constant between these species with OTf^–^ counter‐anions in CD_3_CN at 295 K was estimated to be 1.8 × 10^5^ M^−1^, with a free energy difference of about –30 kJ/mol favoring the formation of the cage structure. The higher stability of the helical cage structure was also supported by DFT calculations (Figure 2e; geometry optimization at the r^2^scan‐3c [62]/CPCM(CH3CN) [63] level and single‐point energy calculations at the ωB97X [64]‐D4 [65]/def2TZVPP [66]/SMD [67]‐DRACO [68](CH_3_CN) level with thermostatistical corrections by the mRRHO approach [69]; see Section S7, Supporting Information). For the mononuclear assembly, the calculations suggest that the *trans*‐Pd**L^A^**_2_ isomer is about 35 kJ/mol more energetically favorable than a hypothetical *cis*‐isomer (Table S6).

In contrast to **L^A^**, the reaction of **L^B^** featuring a rigid quinoxaline backbone with Pd(II) in CD_3_CN produces only one product (Scheme 1C), exhibiting sharp signals in the ^1^H NMR spectrum whose positions more closely resemble those of the mononuclear Pd**L^A^**_2_ species rather than the Pd_2_ **L^A^**_4_ cage (Figure 2c). The ^1^H DOSY measurements revealed an *r*_H_ value of 8.7 Å for this species, which is larger than for the Pd**L^A^**_2_ chelate but smaller than for the Pd_2_ **L^A^**_4_ cage. Meanwhile, ESI‐MS clearly indicates the presence of ions corresponding exclusively to the mononuclear Pd**L^B^**_2_ complex (Figure 2d). Furthermore, single‐crystal X‐ray diffraction confirmed the assumed *trans*‐Pd**L^B^**_2_ structure in the solid state (Figure 2f). According to DFT calculations, for **L^B^** the formation of the mononuclear *trans*‐Pd**L^B^**_2_ structure is indeed more favorable than that of the Pd_2_ **L^B^**_4_ cage (Δ*G*_calcd_ = +9 kJ/mol, Figure 2f), most probably due to a larger ligand strain energy (+13 kJ/mol per ligand) that the rigid and planar quinoxaline backbones would experience in a cage assembly. The change of counter‐ion (OTf^–^), solvent (CD_3_NO_2_ or DMSO‐*d_6_*), or guest addition (aliphatic or aromatic disulfonates) does not affect the self‐assembly outcome.

### 2.2 Heteroleptic Assembly Formation

We next investigated the ability of the studied ligands to form heteroleptic assemblies in the presence of Pd(II) cations (Scheme 1D,E). As a bis‐monodentate ligand, the open conformation of **L^A^** is expected to have converging metal‐binding vectors due to its isoquinoline donor groups. Therefore, as shape‐complementary partners, we selected 2,7‐di(pyridin‐3‐yl)‐fluoren‐9‐one (**L^C^**) and 3,6‐di(pyridin‐4‐yl)‐9,10‐dimethoxyphenanthrene (**L^D^**), which feature diverging binding vectors. These ligands have previously enabled the clean formation of *cis*‐Pd_2_ **A**_2_ **B**_2_‐type heteroleptic assemblies when combined with converging ligands containing isoquinoline donor groups and rigid acridone or carbazole backbones [22, 70]. Moreover, using **L^C^** in combination with a flexible chiral ligand, we have recently achieved, for the first time, a low‐symmetry isosceles triangular Pd_3_ **A**_2_ **B**_4_ topology [41].

**FIGURE 3:**
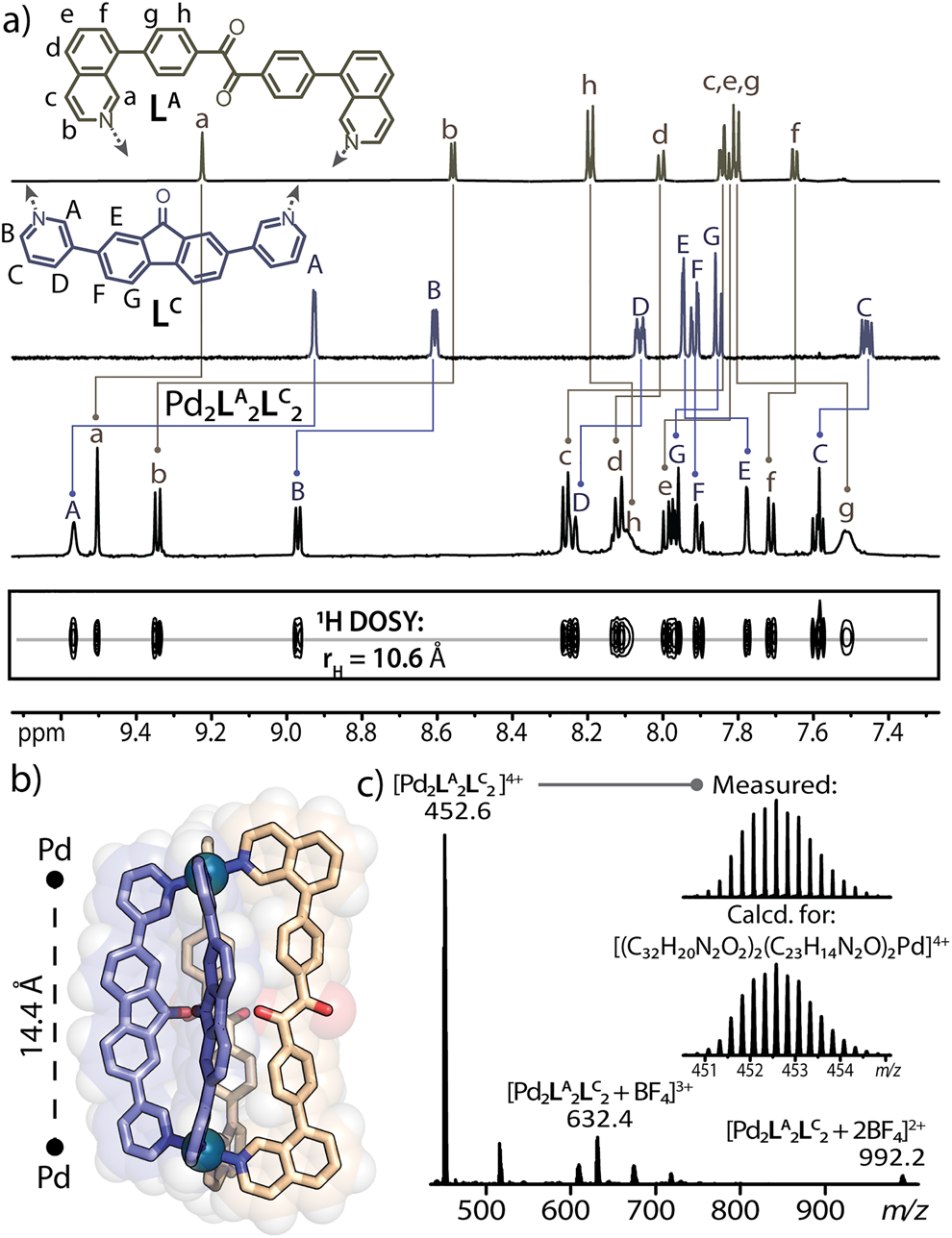
(a) ^1^H NMR spectra (CD_3_CN) of **L^A^** (top), **L^C^** (middle), and Pd_2_ **L^A^**_2_ **L^C^**_2_ (bottom) species with DOSY trace for Pd_2_ **L^A^**_2_ **L^C^**_2_; (b) X‐ray structure of heteroleptic cage Pd_2_ **L^A^**_2_ **L^C^**_2_ with Pd–Pd distance. (c) ESI‐MS spectrum of cage Pd_2_ **L^A^**_2_ **L^C^**_2_. Minor peaks belong to traces of homoleptic [Pd_2_ **L^A^**_4_]^4+^ (m/z = 517.1), [Pd_2_ **L^A^**_4_ + BF_4_]^3+^ (719.1), and [Pd_2_ **L^C^**_2_(CX_3_CN)_4_ + 2BF_4_]^2+^ (X = H, D; 610.1) and heteroleptic [Pd_2_ **L^A^**_3_ **L^C^** + BF_4_]^3+^ (632.4).

The reaction of **L^A^**, **L^C^**, and [Pd(CH_3_CN)_4_](BF_4_)_2_ in a 1:1:1 ratio at 70°C for 90 h resulted in the formation of a new species (Scheme 1D), showing one distinct set of signals for both **L^A^** and **L^C^** ligands, different from those of the free ligands and their corresponding homoleptic assemblies (Figure 3a). The same product could also be obtained through the reaction of the corresponding homoleptic cages (Figure S30). ^1^H DOSY measurements revealed an *r*_H_ value of 10.6 Å for this species (Figure 3a), which is larger than that of the homoleptic Pd_2_ **L^A^**_4_ cage. We were successful in obtaining an X‐ray structure of the *cis*‐Pd_2_ **L^A^**_2_ **L^C^**_2_ cage that showed a Pd–Pd distance increase to 14.4 Å as compared to the homoleptic Pd_2_ **L^A^**_4_ helicate, consistent with the DOSY results (Figure 3b). Furthermore, ESI‐MS analysis confirmed the proposed stoichiometry, showing ions corresponding to [Pd_2_ **L^A^**_2_ **L^C^**_2_ + nBF_4_]^4–^ *^n^* (*n* = 0–2; Figure 3c). Notably, altering the component ratio of **L^A^**, **L^C^**, and Pd(II) did not lead to the formation of any other new discrete species.

**FIGURE 4:**
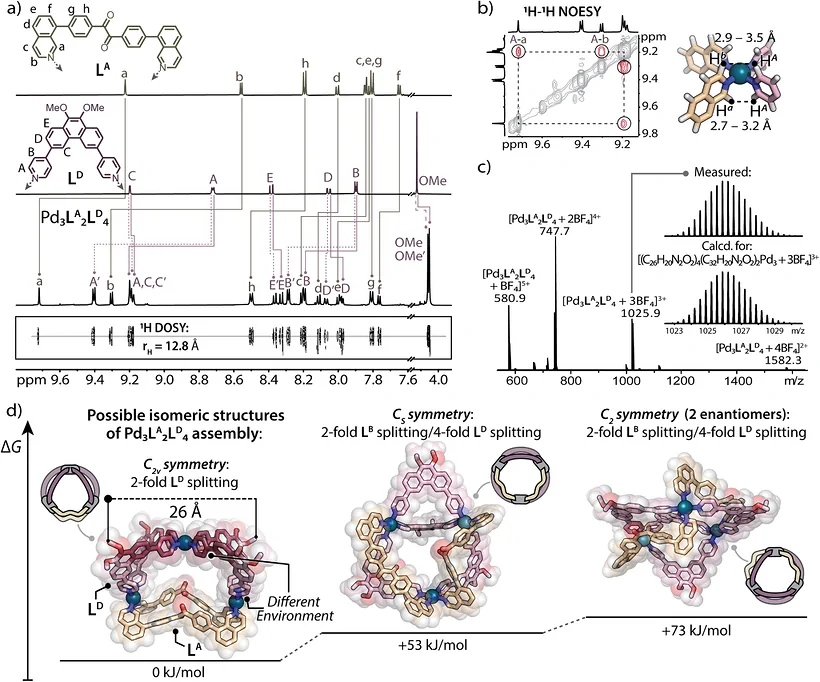
(a) ^1^H NMR spectra (CD_3_CN) of **L^A^** (top), **L^D^** (middle), and Pd_3_ **L^A^**_2_ **L^D^**_4_ (bottom) with the associated DOSY trace for Pd_3_ **L^A^**_2_ **L^D^**_4_; (b) fragment of ^1^H‐^1^H NOESY spectrum (CD_3_CN) of Pd_3_ **L^A^**_2_ **L^D^**_4_ with selected cross‐peaks confirming heteroleptic assembly and the depiction of corresponding inter‐ligand contacts from the DFT model; (c) ESI mass spectrum of Pd_3_ **L^A^**_2_ **L^D^**_4_; (d) DFT models of possible Pd_3_ **L^A^**_2_ **L^C^**_4_ isomers and calculated free energy differences, supporting the isosceles triangular ring structure.

Meanwhile, conducting the reaction of Pd(II) cations with ligands **L^A^** and **L^D^** in a 1:1:1 ratio resulted in a mixture, with some NMR signals corresponding to Pd_n_ **L^A^**_2n_ (*n* = 1, 2) species, while others were distinct from those of free **L^D^** or Pd_n_ **L^D^**_2n_ (*n* = 3, 4) assemblies. Further addition of **L^D^** and Pd(II) to the reaction mixture resulted in a cleaner NMR spectrum and a decrease in the intensity of the Pd_2_ **L^A^**_4_/Pd**L^A^**_2_ signals. Based on these observations, we conducted the reaction using a stoichiometry of **L^A^**, **L^D^**, and Pd(II) in a 2:4:3 ratio, which allowed the exclusive formation of a single clean assembly (Scheme 1E and Figure 4a). Moreover, the same species can also be obtained through cage‐to‐cage transformation from the corresponding homoleptic cages Pd_n_ **L^A^**_2n_ (*n* = 1, 2) and Pd_n_ **L^D^**_2n_ (*n* = 3, 4). However, in this case, the assembly proceeds much more slowly and less cleanly as compared to the direct reaction of Pd(II) with the ligands (Figure S31).

Single crystals of this assembly were obtained; however, they exhibited very weak diffraction, even under synchrotron irradiation. Therefore, to elucidate the structure, we employed detailed NMR and MS analyses combined with DFT calculations (Figure 4). The ^1^H DOSY measurements confirmed that all NMR signals of the heteroleptic assembly belong to a single species with an *r*_H_ of 12.8 Å (Figure 4a), while ^1^H–^1^H NOESY analysis revealed NOE interactions between the protons in the *ortho* positions of the isoquinoline and pyridine moieties of **L^A^** and **L^D^** (Figure 4b), further confirming the incorporation of both ligands into a single molecule. The ESI mass spectrum revealed the presence of [Pd_3_ **L^A^**_2_ **L^D^**_4_ + nBF_4_]^6–n^ (*n* = 1–4) ions, definitively confirming a rare Pd_3_ **L^A^**_2_ **L^D^**_4_ heteroleptic stoichiometry (Figure 4c). For such a Pd_3_ **L^A^**_2_ **L^D^**_4_ composition, three isomers are conceivable [41, 46], exhibiting *C_2v_*, *C_s_*, and *C_2_* (with two enantiomers) symmetries (Figure 4d). The ^1^H NMR spectrum of Pd_3_ **L^A^**_2_ **L^D^**_4_ shows a single set of signals for the **L^A^** ligand and a twofold splitting of the **L^D^** signals, resulting in three sets of ligand signals with a 1:1:1 integral ratio (Figure 4a). This pattern is only consistent with the *C_2v_*‐symmetric isosceles triangular topology, in which the symmetry is broken only for the **L^D^** ligands, as they are neighbored on one side by identical **L^D^** ligands and on the other side by **L^A^** ligands. In contrast, the other two isomers with *C_s_* and *C_2_* symmetries would produce at least a twofold splitting of the **L^A^** signals and a fourfold splitting of the **L^C^** signals, which was not observed. Geometrical analysis of the modelled isosceles triangular Pd_3_ **L^A^**_2_ **L^D^**_4_ structure indicates that the longest inter‐fragment distances are approximately 25–26 Å, consistent with the ^1^H DOSY measurements. Furthermore, DFT calculations indicate that this topology is the most energetically favorable structure among all Pd_3_ **L^A^**_2_ **L^D^**_4_ isomers (by –53 and –73 kJ/mol, respectively, Figure 4d) and also more stable than a binuclear heteroleptic *cis*‐Pd_2_ **L^A^**_2_ **L^D^**_2_ cage (by –8 kJ/mol for the transformation: 4 × Pd_2_ **L^A^**_2_ **L^D^**_2_ + Pd_4_ **L^D^**_8_ ⇌ 4 × Pd_3_ **L^A^**_2_ **L^D^**_4_; Figure S59 and Table S7). Interestingly, for the **L^C^** ligand (which was previously employed by us to access a comparable isosceles triangular Pd_3_ **A**_2_ **B**_4_ topology [41]), the experimentally found binuclear heteroleptic Pd_2_ **L^A^**_2_ **L^C^**_2_ complex is significantly more stable than a tentative Pd_3_ **L^A^**_2_ **L^C^**_4_ species, both according to DFT calculations (by –188 kJ/mol for the 3 × Pd_3_ **L^A^**_2_ **L^C^**_4_ ⇌ 3 × Pd_2_ **L^A^**_2_ **L^C^**_2_ + Pd_3_ **L^C^**_6_ transformation; Figure S59 and Table S7) and according to the experimental observations.

Remarkably, both heteroleptic cages Pd_2_ **L^A^**_2_ **L^C^**_2_ and Pd_3_ **L^A^**_2_ **L^D^**_4_ can be partially interconverted by adding the corresponding ligands **L^C^** or **L^D^** and additional Pd(II) (Scheme 1F). Addition of 2 eq. of **L^C^** to Pd_3_ **L^A^**_2_ **L^D^**_4_ induces partial conversion to Pd_2_ **L^A^**_2_ **L^C^**_2_, giving a 3:2 ratio of Pd_3_ **L^A^**_2_ **L^D^**_4_ to Pd_2_ **L^A^**_2_ **L^C^**_2_ after 18 h at RT, which is then not changing anymore over time (Figure S32). Conversely, adding 1 eq. of Pd(II) and 4 eq. of **L^D^** to Pd_2_ **L^A^**_2_ **L^C^**_2_ yields the same ratio, 3:2 in favor of Pd_3_ **L^A^**_2_ **L^D^**_4_ after 57 h at RT. In both cases, free ligands are released, and the formation of additional unidentified species is observed (Figure S33). Subsequent heating at 70°C does not noticeably promote the transformation further but instead decreases the concentration of both cages and results in the formation of more side products.

We also tested the formation of Pd(II) heteroleptic assemblies involving the quinoxaline‐backboned ligand **L^B^** and either **L^C^** or **L^D^**; however, in these cases, only the corresponding homoleptic structures formed in narcissistic coexistence, as these ligands appear to be not shape‐complementary to each other.

### 2.3 Guest Binding and Guest‐Induced Transformations

Next, we examined the guest‐binding properties of the obtained assemblies using three disulfonate dianions of increasing size: ethane‐1,2‐disulfonate (**G1**), propane‐1,3‐disulfonate (**G2**), and benzene‐1,4‐disulfonate (**G3**) (Scheme 1G,H).

**FIGURE 5:**
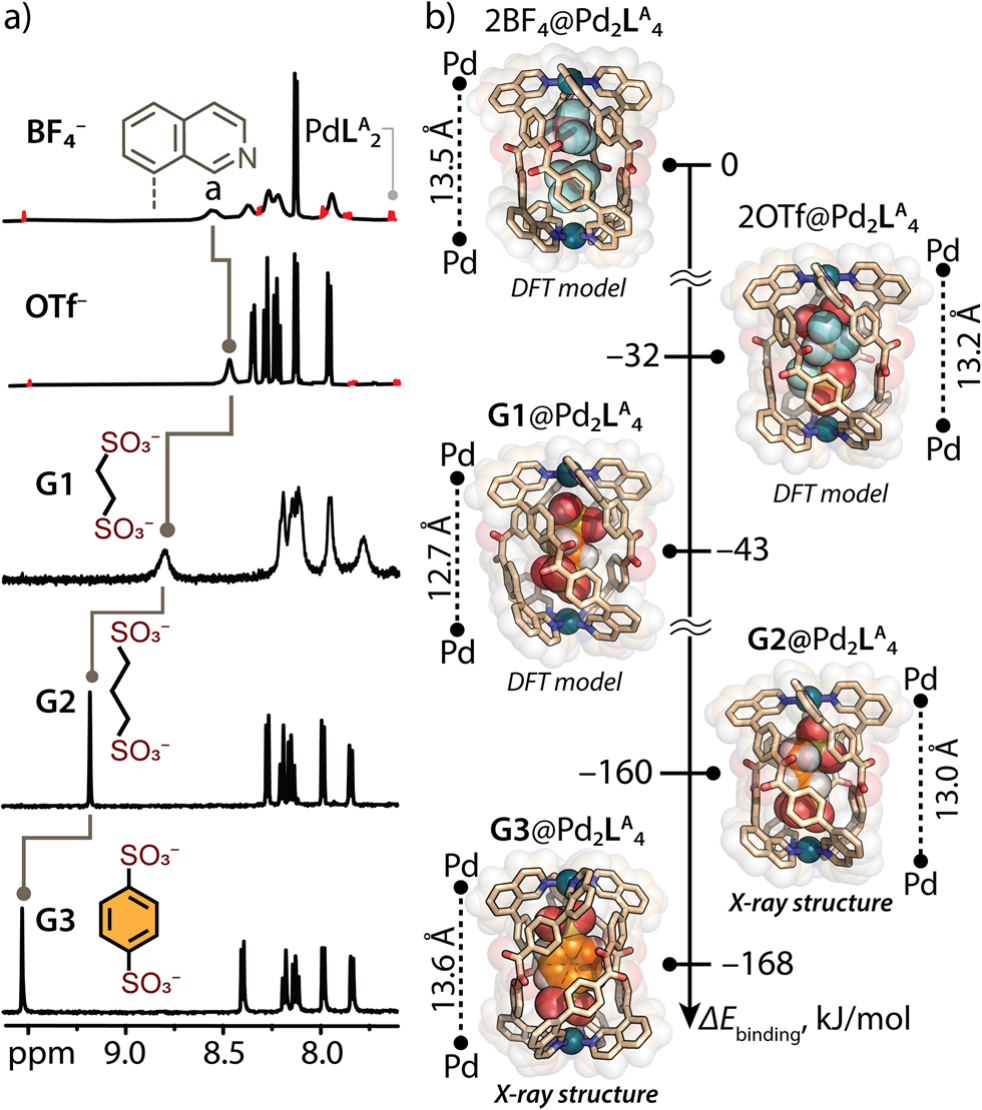
(a) Partial ^1^H NMR spectra (CD_3_CN) of (BF_4_)_2_@[Pd_2_ **L^A^**_4_], (OTf)_2_@[Pd_2_ **L^A^**_4_]_,_ **G1**@[Pd_2_ **L^A^**_4_]_,_ **G2**@[Pd_2_ **L^A^**_4_], **G3**@[Pd_2_ **L^A^**_4_]; (b) optimized DFT models and X‐ray structures of host‐guest complexes with various guests as well as theoretically calculated relative guest binding energies.

Binding of all three guests by the homoleptic Pd_2_ **L^A^**_4_ cage in CD_3_CN proceeds in the slow exchange regime in a 1:1 ratio as indicated by NMR and ESI‐MS data (Figures S34–S38) as well as X‐ray crystallography for the host‐guest complexes with **G2** and **G3** (Figure 5 and Figure S57). Furthermore, the addition of guests results in the disappearance of any residual mononuclear Pd**L^A^**_2_ species. Similar to what was observed for assembly formation with different counteranions (BF_4_^–^ or OTf^–^), the NMR profiles of the host‐guest complexes were found to strongly depend on the nature of the anionic guest. Binding of the smallest disulfonate guest **G1** resulted in broadened signals, thus indicating a highly dynamic structure. At the same time, binding of **G2** and **G3** resulted in mostly sharp NMR profiles with a noticeably down‐field shifted signal corresponding to the ***a*** protons of the isoquinoline moieties, indicating hydrogen bond formation between host and guest [71]. Guest displacement experiments indicate that **G1** can be fully displaced by **G2** or **G3** in Pd_2_ **L^A^**_4_, while the addition of **G2** to **G3**@Pd_2_ **L^A^**_4_ results in a 2:3 **G2**@Pd_2_ **L^A^**_4_ to **G3**@Pd_2_ **L^A^**_4_ equilibrium mixture (Figure S37). These results are also in line with DFT calculations, suggesting the following guest affinity row for Pd_2_ **L^A^**_4_ cage: BF_4_^–^ < OTf^–^ < **G1** < **G2** < **G3** (Figure 5b), thus correlating with guest size [72]. Notably, the flexible and dynamic nature of **L^A^** allows the Pd_2_ **L^A^**_4_ cage to expand and contract, adjusting its size depending on the encapsulated guest. This behavior is evidenced by Pd–Pd distance variations observed in the solid‐state structures of the host–guest complexes (13.0 Å for **G2** vs. 13.6 Å for **G3**), as well as by corresponding differences in the ^1^H DOSY‐based hydrodynamic radii (9.6 Å for **G2** vs 9.8 Å for **G3**, Table S5).

For the heteroleptic cages Pd_2_ **L^A^**_2_ **L^C^**_2_ and Pd_3_ **L^A^**_2_ **L^D^**_4_, binding of **G1** proceeds rather in a fast‐exchange regime. Pd_2_ **L^A^**_2_ **L^C^**_2_ exhibits *exo*‐binding of **G1** indicated by the shift of only outside pointing protons (Figure S39), which also agrees with the x‐ray data indicating a very small cavity, allowing only solvent molecules to fit inside (Figure S55). At the same time, Pd_3_ **L^A^**_2_ **L^D^**_4_, with a larger cavity, mainly shows shifting of inside pointing protons, indicating encapsulation (Figure S40). Both assemblies gradually precipitate upon the addition of more than 1 eq. of **G1**, preventing us from determining association constants. Precipitation was also observed when 1 eq. or more of **G2** or **G3** was added. In contrast, the addition of only 0.5 eq. of **G2** or **G3** did not induce immediate precipitation but led to the appearance of NMR signals corresponding to the **G2**/**G3**@Pd_2_ **L^A^**_4_ host‐guest complexes (Figures S41 and S42). The strong affinity of homoleptic Pd_2_ **L^A^**_4_ for **G2** and **G3**, combined with the conformational flexibility of ligand **L^A^**, thus enables a retro‐cage‐to‐cage transformation in which the heteroleptic assemblies revert back to the homoleptic Pd_2_ **L^A^**_4_ cage (Scheme 1H) [14]. As both heteroleptic cages contain only half of the homoleptic cage (corresponding to two **L^A^** units), 0.5 eq. of the guests should be the stoichiometrically required amount for this transformation. At room temperature, the retro‐cage‐to‐cage transformations are very slow. At 70°C, heating Pd_2_ **L^A^**_2_ **L^C^**_2_ with 0.5 eq. of guest for about 4 days results in approximately 50% conversion of Pd_2_ **L^A^**_2_ **L^C^**_2_ to **G3**@Pd_2_ **L^A^**_4_ and about 40% conversion to **G2**@Pd_2_ **L^A^**_4_, consistent with the established guest‐affinity trends of Pd_2_ **L^A^**_4_. Further heating leads to gradual precipitation of both systems. For Pd_3_ **L^A^**_2_ **L^D^**_4_, formation of **G2**/**G3**@Pd_2_ **L^A^**_4_ complexes is also observed by NMR and ESI‐MS (Figures S43–S45), but precipitation occurs even more rapidly upon heating, not allowing estimation of the conversion values for the retro‐cage‐to‐cage transformations in this case.

### 2.4 Post‐Assembly Condensation With 1,2‐Phenylenediamine

Finally, we examined the possibility of a post‐assembly transformation of the 1,2‐dicarbonyl backbone of **L^A^** within the homo‐ and heteroleptic assemblies by condensation with stoichiometric amounts of 1,2‐phenylenediamine (**PDA**) in CD_3_CN (Scheme 1I and J, Figures S46–S56).

The uncomplexed ligands **L^A^** and **L^B^** are only partially soluble in CD_3_CN (maximal concentration approximately 0.3 mM ≈ 10% of the concentration used in the self‐assemblies), but free **L^A^** still reacts with **PDA** at room temperature slightly faster than when it is part of an assembly within the initial reaction phase (Figure S56). For the homoleptic Pd**L^A^**_2_ and Pd_2_ **L^A^**_4_ systems, we observed slight dependence of the reaction rate on the assembly nuclearity. At RT, during the early stages of the reaction, the freshly formed kinetic Pd**L^A^**_2_ species undergoes condensation with **PDA** producing Pd**L^B^**_2_ faster than the thermodynamic Pd_2_ **L^A^**_4_ cage product (Figure S56). However, in parallel with the condensation, Pd**L^A^**_2_ also dimerizes to the Pd_2_ **L^A^**_4_ cage structure (representing the faster process), and the **PDA**‐related reaction rates for the two assemblies converge as the conversion progresses.

During the reaction with **PDA**, minor release of free **L^A^** was observed by NMR in both cases (which was subsequently consumed), whereas no formation of free **L^B^** was detected. In addition, several transient intermediate or side species were detected by NMR, including a heteroleptic mononuclear Pd**L^A^L^B^** complex, a Pd(II)‐bound **PDA** species, and other unidentified species that were gradually consumed during the reaction (Figures S47 and S48). Analysis of the reaction mixture of Pd_2_ **L^A^**_4_ with **PDA** by ESI‐MS revealed several possible reaction intermediates (Figure S49). Consistent with the NMR results, ions corresponding to the Pd**L^A^L^B^** complex were observed, whereas no signals corresponding to heteroleptic cage species Pd_2_ **L^A^** *_x_* **L^B^** *_y_* (*x* + *y* = 4) were detected. Moreover, signals corresponding to mono‐condensation products of **PDA** with the dicarbonyl group of **L^A^** coordinated to a single Pd(II) center were observed, namely [Pd**L^A^**_2_ + n**PDA** − nH_2_O]^2+^ (*n* = 1, 2) ions. In contrast, no signals corresponding to mono‐condensation products of the free ligand and cage structures were detected. These results indicate that the reaction most likely proceeds via the mononuclear Pd(II) assemblies, which exist in equilibrium with the cage structure and adopt a more suitable *syn*‐conformation concerning the central dicarbonyl moiety for condensation with **PDA**. Theoretical calculations further suggest that the formation of even a single **L^B^** ligand within the Pd_2_ **L^A^**_4_ cage would introduce significant strain (Figure S60), substantially reducing cage stability and promoting its dissociation into mononuclear species, which were detected as reaction intermediates.

Interestingly, the counterion (or encapsulated guest) within the Pd_2_ **L^A^**_4_ cage can significantly affect the condensation process. The formation rate of Pd**L^B^**_2_ decreases as the affinity of the Pd_2_ **L^A^**_4_ cage for the anionic guest increases, following the sequence BF_4_^–^ > **G1** > **G2** > **G3** (Figure S56). Noteworthy, for the **G1**@Pd_2_ **L^A^**_4_ and **G2**@Pd_2_ **L^A^**_4_ host‐guest complexes with aliphatic disulfonates, we observed slower reaction rates than for the Pd_2_ **L^A^**_4_ host, but they were accompanied by a more pronounced release of free **L^A^** and minor formation of uncomplexed **L^B^** (Figures S50 and S51). In addition, signals corresponding to free Pd_2_ **L^A^**_4_ host were detected after addition of **PDA** indicating the guest release during the reaction. In contrast, for the **G3**@Pd_2_ **L^A^**_4_ complex, which exhibits the slowest condensation rate, only trace amounts of free **L^A^** were observed, with no formation of free **L^B^** or Pd_2_ **L^A^**_4_ (Figure S52).

Heteroleptic cages Pd_2_ **L^A^**_2_ **L^C^**_2_ and Pd_3_ **L^A^**_2_ **L^D^**_4_ undergo condensation with **PDA** also noticeably slower than the homoleptic Pd_2_ **L^A^**_4_ assembly. Notably, while Pd_3_ **L^A^**_2_ **L^D^**_4_ remains relatively stable in the presence of **PDA** but gradually reacts with it at RT, Pd_2_ **L^A^**_2_ **L^C^**_2_ shows almost no conversion to Pd**L^B^**_2_ at all. Instead, it predominantly undergoes disassembly, releasing free **L^C^** as well as the forming Pd(II) complex of **PDA** and some unidentified species as side products (Figures S53 and S54). Analysis of the reaction mixtures containing heteroleptic cages by ESI‐MS did not reveal any additional intermediate species beyond those observed for the reaction with the Pd_2_ **L^A^**_4_ complex, indicating that in this case the reaction most likely also proceeds via a mononuclear complex. Under heating conditions (70°C in CD_3_CN), homoleptic cage Pd_2_ **L^A^**_4_ (with BF_4_^–^ counterion) converts to Pd**L^B^**_2_ with approximately 80% yield within 24 h. At the same time, Pd_3_ **L^A^**_2_ **L^D^**_4_ yields only about 60% of Pd**L^B^**_2_ after 7 days under the same conditions (Figure S55). For Pd_2_ **L^A^**_2_ **L^C^**_2_, complete consumption of the starting cage is observed after 1.5 days at 70°C, but only approximately 30% Pd**L^B^**_2_ is formed, with the remainder undergoing decomposition pathways.

### 2.5 Ligand Conformation Analysis and Conformation Effects in Cages

**FIGURE 6:**
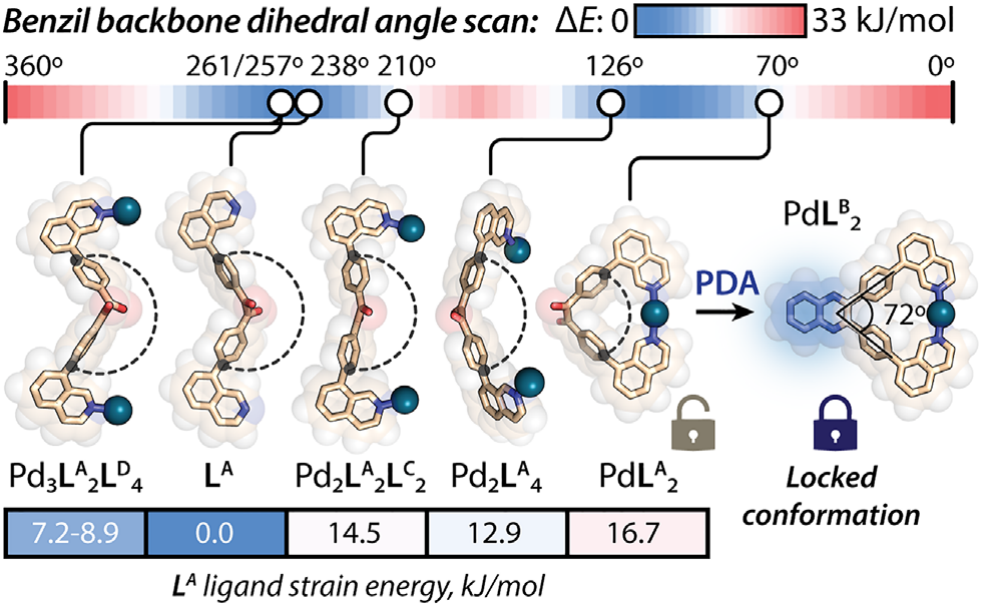
Heat map of relaxed scan calculated energies of the dihedral angle between the phenyl rings of the benzil backbone (top); views of various conformations of ligand **L^A^** with corresponding dihedral angles between phenylene arms and ligand **L^B^** in Pd**L^B^**_2_ (middle); and calculated relative **L^A^** ligand strain energy [Δ*E*_strain_ = *E*(**L^A^**_assembly_) − *E*(**L^A^**_free_)] in homo‐ and heteroleptic Pd(II) assemblies (bottom).

A comprehensive analysis of the X‐ray and DFT‐optimized structures of all homo‐ and heteroleptic assemblies reveals several distinct conformational states that **L^A^** can adopt within Pd(II) supramolecular architectures (Figure 6 and Table S4). These conformations not only populate to fit the geometry of the assembly but also seem to define the reactivity of **L^A^** within the respective assembly as well as the overall assembly stability.

In the mononuclear *trans*‐Pd**L^A^**_2_ complex, **L^A^** adopts a folded conformation with a dihedral angle of approximately 70° between the phenylene arms. This geometry places the dicarbonyl moiety almost in a *syn*‐arrangement, which is optimal for condensation with **PDA** [59], yielding the *trans*‐Pd**L** ^B^_2_ complex that retains a similar inter‐arm angle of 72°. This structural compatibility is consistent with the faster initial kinetics of Pd**L^A^**_2_ condensation relative to the Pd_2_ **L^A^**_4_ cage. In contrast, the helical Pd_2_ **L^A^**_4_ assembly (standing in equilibrium with Pd**L^A^**_2_ in solution) adopts a more open conformation, showing dihedral angles between ligand arms of approximately 126° in the DFT model and 124–141° in the solid‐state structures. While the dicarbonyl groups are oriented outward from the cage cavity, allowing sterically unhindered nucleophilic attack by **PDA**, this conformation departs more strongly from the *syn*‐arrangement than in the mononuclear chelate species. At the same time, the structure of Pd_2_ **L^A^**_4_ is highly dynamic and can adjust its size and shape (e.g., upon guest binding, Figure 5b), which may enable **L^A^** to adopt a more favorable conformation for condensation with **PDA**. Notably, the reactivity of the cage can also be modulated by guest binding. Although the dicarbonyl dihedral angles in the solid‐state and calculated structures of the host‐guest complexes with **G1–G3** do not significantly deviate from those of the free Pd_2_ **L^A^**_4_ host (Table S4), our experiments show that the reactivity of Pd_2_ **L^A^**_4_ can be reduced upon guest encapsulation (especially for **G3**). This is attributed to stabilization of the strongly twisted cage structure, which hinders the central dicarbonyl unit from adopting the *syn*‐conformation required for efficient conversion into the *trans*‐Pd**L^B^**_2_ complex.

The introduction of a second type of ligand can further alter the geometry of **L^A^**, increasing the dihedral angle between its arms to 210° in the optimized Pd_2_ **L^A^**_2_ **L^C^**_2_ structure (approximately 190° in the crystal) or further to 238° and 261° upon incorporation of **L^D^** in the optimized structure of the Pd_3_ **L^A^**_2_ **L^D^**_4_ assembly. Additionally, the symmetry‐breaking incorporation of a second type of ligand is expected to rigidify the assembly structure. In Pd_3_ **L^A^**_2_ **L^D^**_4_, the dicarbonyl groups adopt a similar arrangement to that in Pd_2_ **L^A^**_4_, but they point toward the interior of the cage, where steric effects may hinder interaction with **PDA** and should further reduce the reactivity of the assembly.

In contrast, the Pd_2_ **L^A^**_2_ **L^C^**_2_ cage shows a dicarbonyl orientation closer to an *anti*‐conformation, which is the least favorable for condensation with **PDA**. Also, cage‐internal dynamics that would lead to twisting of the **L^A^** ligand backbones and move the carbonyl substituents into *syn*‐arrangement for at least brief periods is not likely to occur in this non‐helical, low‐symmetry assembly. We thus hypothesize that this geometric arrangement accounts for the lower reactivity of this cage and explains why it mainly undergoes decomposition through Pd(II) abstraction by **PDA**, accompanied by release of the free ligands.

Considering the conformational landscape of the diphenyl‐1,2‐dicarbonyl backbone (evaluated by a relaxed scan of the dihedral angle between the phenyl arms; Figure 6), the conformations adopted by **L^A^** in the optimized structures of the free **L^A^** ligand (dihedral angle of 253°) and of Pd_3_ **L^A^**_2_ **L^D^**_4_ cage (dihedral angles of 238° and 261°) lie closest to the calculated energy minima. In terms of ligand strain, the extended conformations in Pd_3_ **L^A^**_2_ **L^D^**_4_ are less favorable than the free ligand by approximately 7.2–8.9 kJ/mol, likely due to additional strain in the isoquinoline and phenylene moieties arising from adjustment of the ligand geometry to the cage structure. The open conformation in Pd_2_ **L^A^**_4_ is, as expected, even more strained relative to the free ligand, with a strain energy of 12.9 kJ/mol. Even higher strain energies are observed for **L^A^** in Pd**L^A^**_2_ and Pd_2_ **L^A^**_2_ **L^C^**_2_, with Δ*E*_strain_ values of 14.5 and 16.7 kJ/mol, respectively (Figure 6 and Table S7). In both cases, the **L^A^** ligand experiences steric repulsion, either between the phenylene moieties in the folded Pd**L^A^**_2_ structure or between the phenylene and carbonyl moieties in the Pd_2_ **L^A^**_2_ **L^C^**_2_ cage. The calculation results are consistent with experimental observations: the lower ligand strain in Pd_2_ **L^A^**_4_ correlates with its higher stability and greater population relative to Pd**L^A^**_2_ in solution. Among the heteroleptic cages, the less strained Pd_3_ **L^A^**_2_ **L^D^**_4_ species displays, accordingly, a higher stability than the Pd_2_ **L^A^**_2_ **L^C^**_2_ cage.

## 3 Conclusion

In conclusion, we have shown that the intrinsic flexibility and reactivity of the benzil‐based ligand **L^A^** enable it to adopt multiple conformations, giving rise to various homoleptic and heteroleptic Pd(II) assemblies with different nuclearities, topologies, and stimuli‐responsive transformation pathways. In homoleptic systems, **L^A^** can take folded or open conformations, forming the mononuclear complex Pd**L^A^**_2_ and the Pd_2_ **L^A^**_4_ helicate, which—depending on the solvent—can coexist in solution. Incorporation of additional diverging ligands, **L^C^** or **L^D^**, bearing rigid fluorenone or phenanthrene backbones (either through direct Pd(II)‐mediated self‐assembly or via cage‐to‐cage transformation), induces dihedral angle expansion of **L^A^** upon formation of the classic heteroleptic *cis*‐Pd_2_ **L^A^**_2_ **L^C^**_2_ assembly or the rare, low‐symmetry isosceles triangular Pd_3_ **L^A^**_2_ **L^D^**_4_ ring topology. Both heteroleptic cages can be partially interconverted through addition of the corresponding ligands and Pd(II) ions, and both can also undergo partial guest‐induced retro‐cage‐to‐cage transformation back to homoleptic species. Furthermore, **L^A^** can be conformationally locked by condensation of its 1,2‐dicarbonyl backbone with 1,2‐phenylenediamine, generating the pincer‐shaped and rigid quinoxaline‐backboned ligand **L^B^**, which forms only the *trans*‐Pd**L^B^**_2_ chelate complex in the presence of Pd(II). Notably, this transformation can be performed in a post‐assembly manner, using either homoleptic or heteroleptic **L^A^**‐based architectures as precursors. We could show that the conformation adopted by **L^A^** within the different supramolecular assemblies strongly influences both its reactivity toward condensation with 1,2‐phenylenediamine and the overall assembly stability. In homoleptic cages, guest binding can further modulate the reactivity of **L^A^** within the assembly, with higher‐affinity guests exhibiting a stronger inhibitory effect on the reaction.

Overall, our work demonstrates how ligand flexibility and reactivity can be strategically harnessed to control supramolecular geometry, nuclearity, and topology in Pd(II) coordination assemblies, enabling multiple interconversions between different states using ligand, guest, and reagent as stimuli. Observed relationships between ligand conformation, assembly structure, guest presence, and the course of the studied chemical transformations also highlight how hierarchical supramolecular organization can modulate functional group reactivity. Such control over the conformation and accessibility of specific reactive sites can be compared to how nature steers the reactivity of chemical functionality through nanoscopic confinement, conformational restriction, and allosteric regulation. Furthermore, the obtained Pd(II)‐based assembly system can serve as a versatile platform for the design of adaptive, chemically addressable metallosupramolecular architectures, with potential applications in information transduction and processing, selective guest recognition, and stimuli‐responsive catalysis under confinement.

## Author Contributions

G.H.C. and A.S.M. conceived and designed the project. Z.Z., A.P.G., M.A. performed synthetic work, compound characterization, experiments and analysed the data. A.S.M. performed further experiments, theoretical and crystallographic studies, analysed the data, and prepared the initial manuscript draft. B.S. performed DOSY and VT NMR measurements, and processed crystallographic data. G.H.C. and A.S.M. supervised the work. All authors discussed the results, and the manuscript was written through contributions of all authors.

## Conflicts of Interest

The authors declare no conflicts of interest.

## Supporting information




**Supporting File 1**: The authors have cited additional references within the Supporting Information [73, 74, 75, 76, 77, 78, 79, 80, 81, 82, 83, 84, 85, 86, 87, 88, 89].

**Supporting File 2**: anie73516‐sup‐0002‐SuppMat.zip.

## Data Availability

The data that support the findings of this study will be openly available in the RESOLVdata repository in machine‐readable form at https://data.tu‐dortmund.de/dataverse/resolv under the link https://doi.org/10.17877/RESOLV‐2026‐TBVPLR.
