# Supplementary material for: A Multi‐Stimuli Transformational Network of Benzil‐Based Pd(II) Assemblies
Source: Angew Chem Int Ed Engl. 2026 Jul 9;65(36):e9050553. doi: 10.1002/anie.9050553 (PMC13528552; doi:10.1002/anie.9050553)
Supplement: Supplementary file 1 [file ANIE-65-e9050553-s002.pdf]

Supporting information for

A Multi-Stimuli Transformational Network of Benzil-based Pd(II) Assemblies

Zhiwei Zeng, A. Priscila Gia, Alexander Mikherdov,* Mert Acar, Björn Schmidt, and Guido H. Clever*

Department of Chemistry and Chemical Biology,

TU Dortmund University, Otto-Hahn Straße 6, 44227, Dortmund, Germany,

E-mail: aleksandr.mikherdov@tu-dortmund.de

E-mail: guido.clever@tu-dortmund.de

Contents

S1. General Information 3

S2. Ligand syntheses and characterization 4

S2.1 Synthesis of L^A ligand 4

S2.2 Synthesis of L^B ligand 6

S3. Self-assembly of coordination cages 9

S3.1 Self-assembly of $[Pd_nL^A{}_{2n}](X)_{2n}$ 9

S3.2 Self-assembly of $PdL^B{}_2$ 13

S3.3 Self-assembly of $Pd_2L^A{}_2L^C{}_2$ 15

S3.4 Self-assembly of $Pd_3L^A{}_2L^D{}_4$ 18

S3.5 Cage-to-cage transformations 20

S4. Host-guest study 23

S4.1 Host-guest study of homoleptic and heteroleptic cages. 23

S4.2 Guest-induced transformation of heteroleptic cages 26

S5. Post-assembly modification 30

S6. X-ray crystallography 40

S6.1 Crystal samples preparation 40

S6.2 X-ray data collection and refinement details 40

S7. DFT calculations 45

S7.1 Calculations details 45

S7.2 Assemblies relative stability 47

S7.3 Guest binding energies 49

S7.4 Ligand conformational analysis 49

S8. References 54

S1. General Information

Unless otherwise stated, all chemicals and standard solvents were purchased from Sigma Aldrich, Acros Organics, Carl Roth, TCI Europe, VWR, ABCR, or other suppliers and were used as received. Deuterated solvents were obtained from Eurisotop. For synthesis, HPLC-grade solvents were used, and for purification, technical-grade solvents were employed. Where necessary, experiments were conducted under a nitrogen atmosphere using standard Schlenk techniques. Reactions were monitored using thin-layer chromatography (TLC) on silica-coated aluminium plates (Merck, silica 60, fluorescence indicator F254, thickness 0.25 mm). For column chromatography, silica (Merck, silica 60, 0.02–0.063 mesh ASTM) was used as the stationary phase unless stated otherwise. Ligand $\mathbf{L^C}$ and $\mathbf{L^D}$ were obtained according to the literature reported procedures.[39,main text, 69]

NMR spectroscopic data was measured on the spectrometers Bruker AV 500 Avance NEO, AV 600 Avance NEO, or AV 700 Avance NEO. For ^{1}H and ^{13}C NMR spectra, chemical shifts were calibrated to the solvent lock signal. Chemical shifts δ are given in ppm, coupling constants J in Hz. All spectra were recorded in standard 5 mm NMR tubes. All proton and carbon signals were assigned with the aid of 2D NMR spectra. ^{1}H DOSY NMR spectra were recorded with a *dstebpgp3s* pulse sequence with diffusion delays D20 of 0.08 s and gradient powers P30 of 1400 µs.[70,71] The data analyses of the corresponding signals in the 1D spectra were performed in Dynamics Center 2.8.9 (Bruker BioSpin, Rheinstetten, Germany) to obtain the diffusion coefficients D using the Stejskal–Tanner equation.[72,73] Hydrodynamic radii r_H were calculated using the Stokes–Einstein equation.

Mass spectra and trapped ion mobilograms were recorded with Bruker ESI timsTOF spectrometer (positive mode). For the calibration, AgilentTM ESI Low Concentration Tuning Mix was used.

S2. Ligand syntheses and characterization

S2.1 Synthesis of L^A ligand

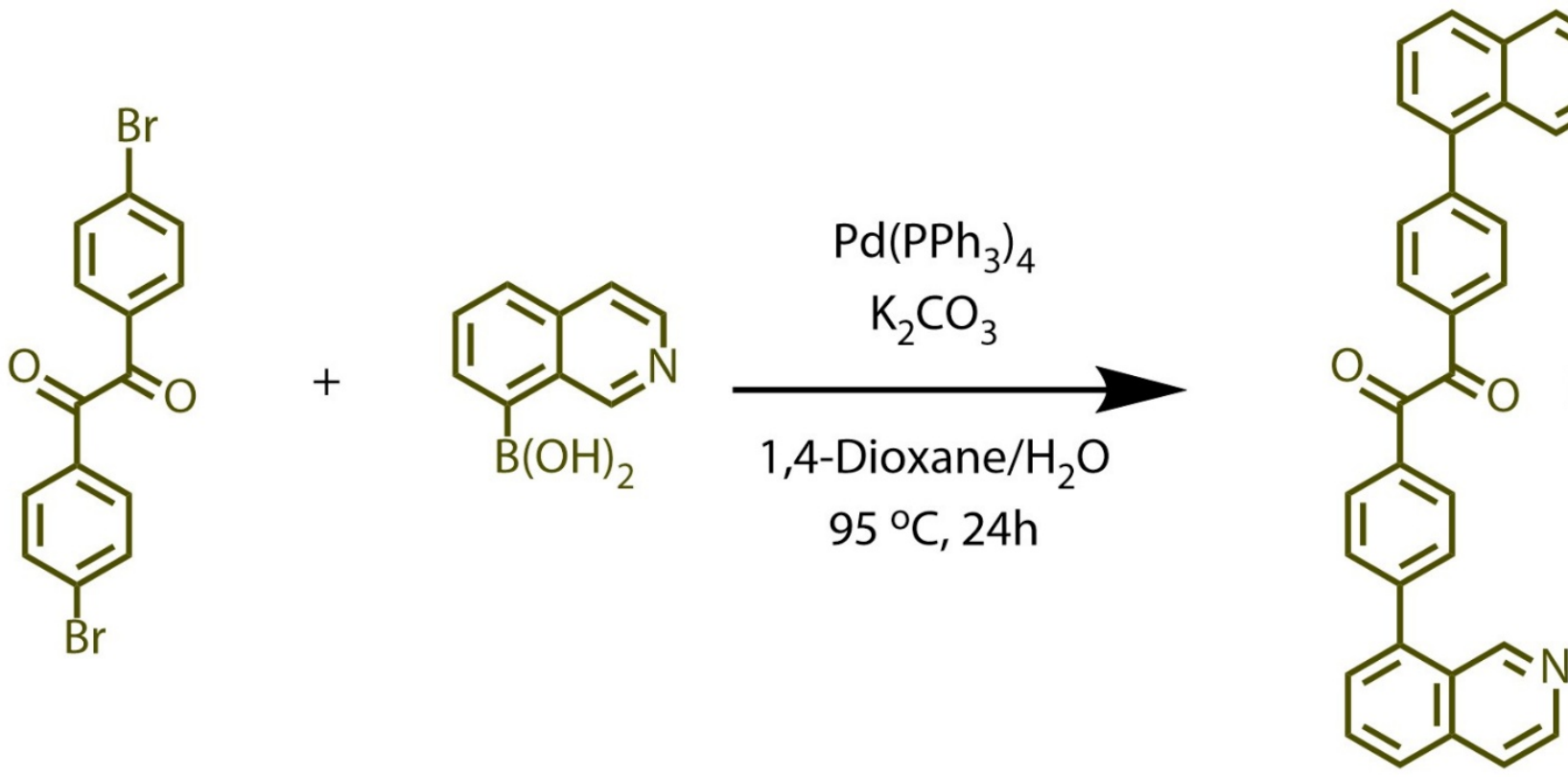

L^A

Scheme S1. Synthesis of **L^A** ligand.

4,4'-Dibromobenzil (183 mg, 0.50 mmol), isoquinoline-8-boronic acid (259 mg, 1.50 mmol), and K_2CO_3 (249 mg, 1.80 mmol) were added to a mixture of 1,4-dioxane (20 mL) and water (4 mL). The solution was degassed by bubbling nitrogen for 15 min, after which $Pd(PPh_3)_4$ (58 mg, 0.05 mmol) was added. The reaction mixture was heated at 95 °C and stirred for 24 h. After cooling to room temperature, the product was extracted with dichloromethane. The organic phase was dried over $MgSO_4$ and concentrated under reduced pressure. The crude product was collected and washed with acetone to afford **L^A** as a yellow solid (149 mg, 64% yield).

^{1}H NMR (500 MHz, CD_3CN) δ 9.22 (s, 2H), 8.56 (d, *J* = 5.7 Hz, 2H), 8.21 – 8.18 (m, 4H), 8.00 (d, *J* = 8.3 Hz, 2H), 7.85 (d, *J* = 3.0 Hz, 2H), 7.84 (d, *J* = 1.2 Hz, 2H), 7.82 – 7.79 (m, 4H), 7.65 (dd, *J* = 7.1, 1.1 Hz, 2H).

^{13}C NMR (151 MHz, CD_3CN) δ 195.62, 151.16, 146.74, 144.15, 140.06, 137.20, 133.20, 132.03, 131.05, 131.03, 129.57, 128.22, 127.02, 121.65.

HRMS-ESI $[C_{32}H_{20}N_2O_2 + H]^+$ Calculated (m/z): 465.1598, found: 465.1557.

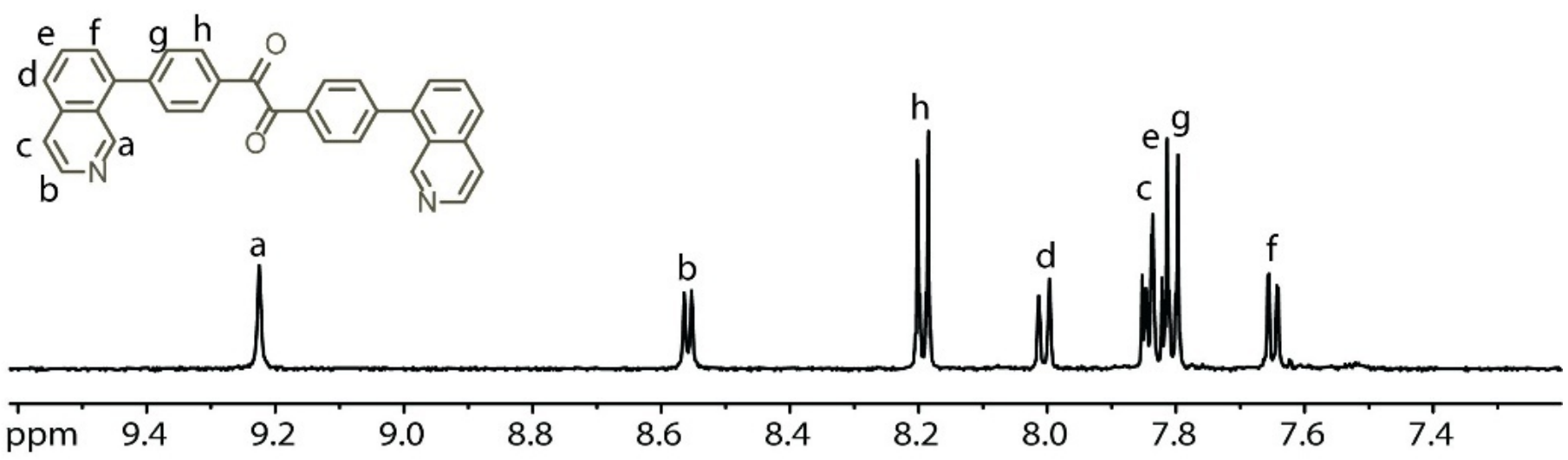

Figure S1. ^{1}H NMR spectrum (500 MHz, 298K, CD_3CN) of **L[A]**.

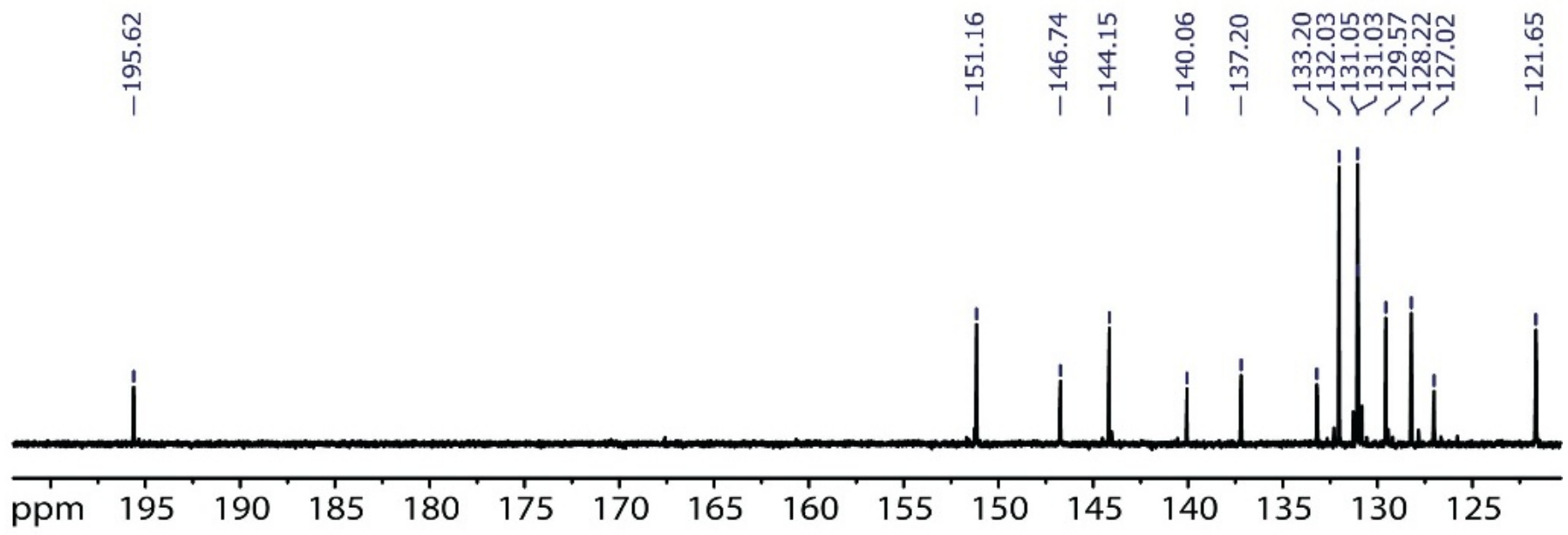

Figure S2. ^{13}C NMR spectrum (151 MHz, 298K, CD_3CN) of **L[A]**.

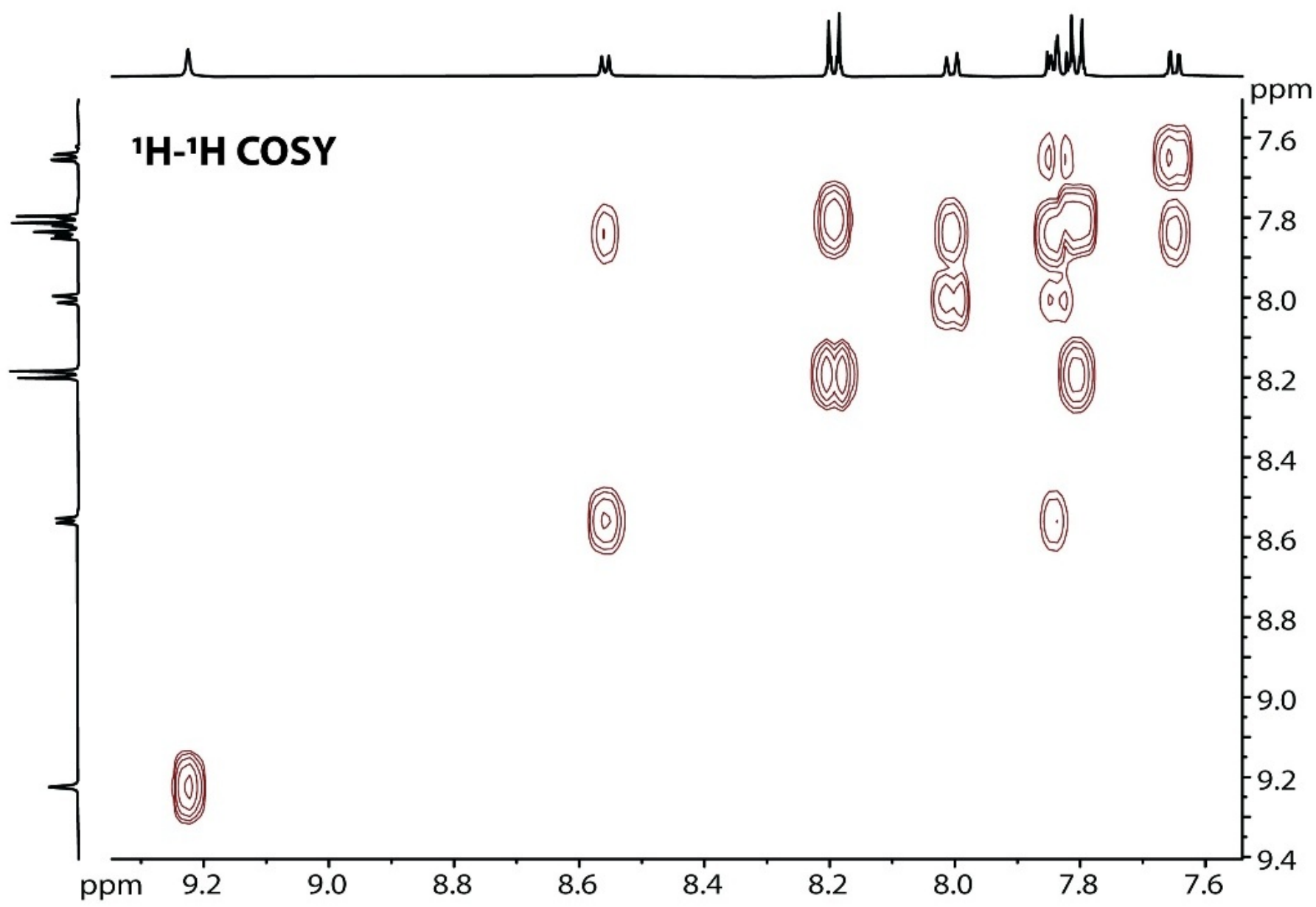

Figure S3. Partial ^{1}H-^{1}H COSY spectrum (600 MHz, 298K, CD_3CN) of **L[A]**.

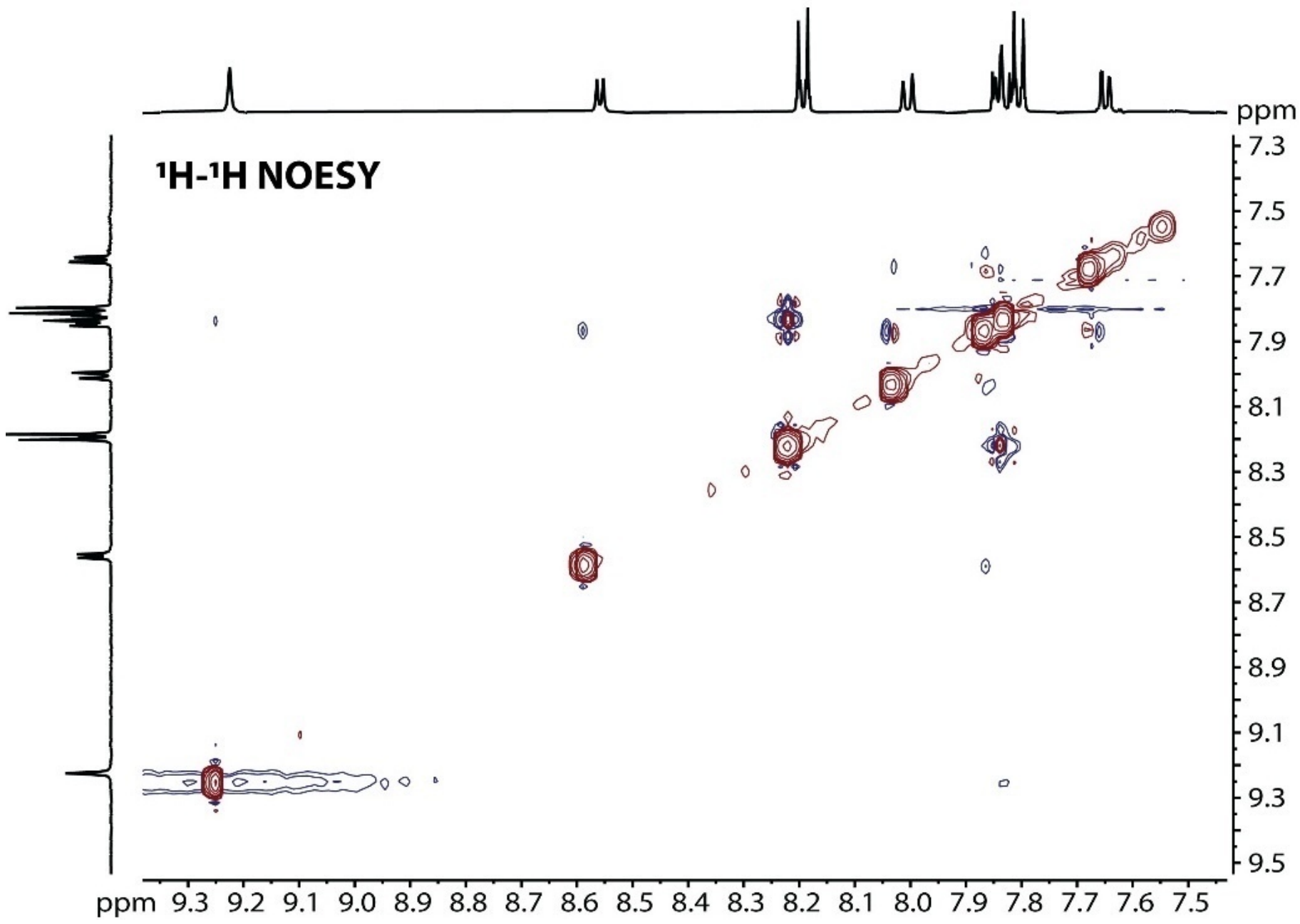

Figure S4. Partial ^{1}H-^{1}H NEOSY spectrum (600 MHz, 298K, CD_3CN) of **L^A**.

S2.2 Synthesis of L^B ligand

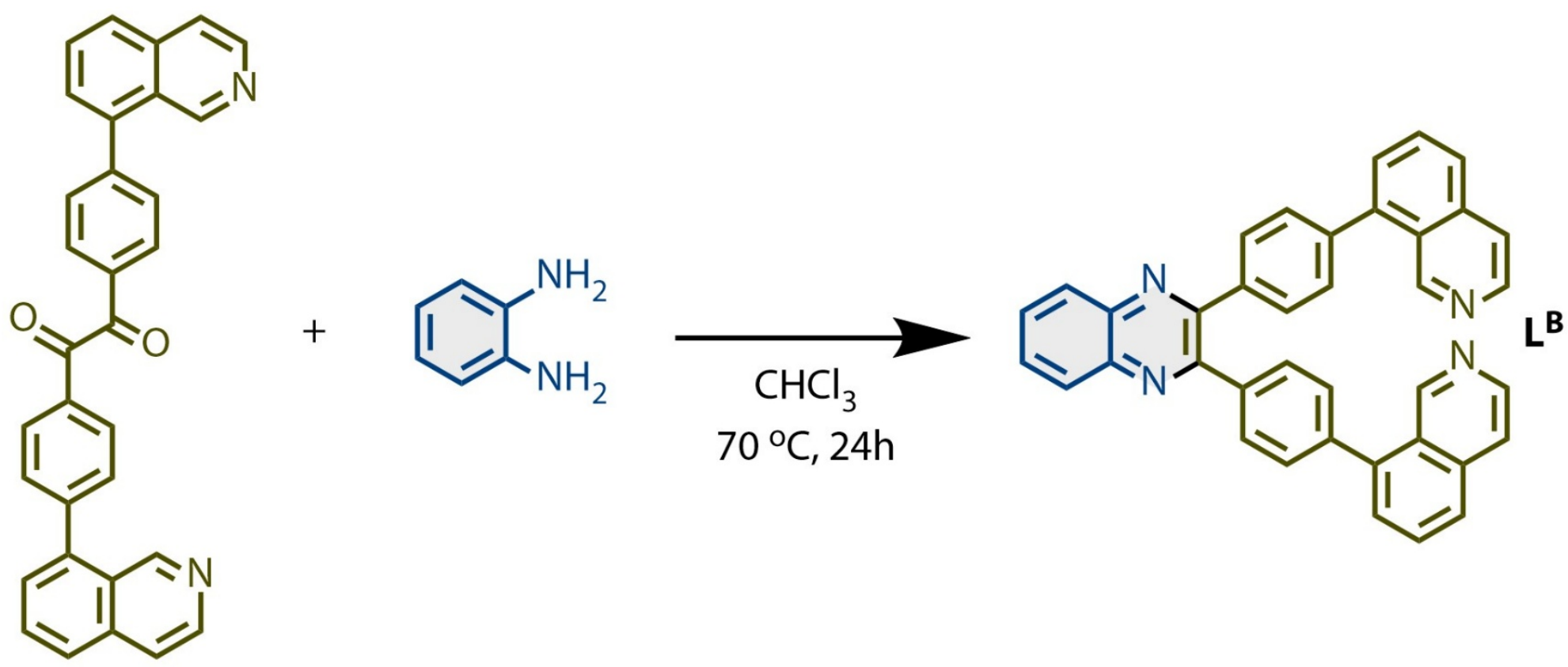

Scheme S2. Synthesis of **L^B** ligand.

1,2-bis(4-(isoquinolin-8-yl)phenyl)ethane-1,2-dione (11.6 mg, 0.025 mmol) and *o*-phenylenediamine (3.2 mg, 0.03 mmol) were added in chloroform (3 mL). The reaction mixture was heated at 70 °C and stirred for 24 h. After the mixture was cooled to room temperature, the solvents were removed under reduced pressure, and the crude product was collected and further washed with diethyl ether to obtain the pure **LB** as pale-grey solid (10.5 mg, 78% yield).

^{1}H NMR (700 MHz, CD3CN) δ 9.27 (s, 2H), 8.53 (d, J = 5.8 Hz, 2H), 8.22 (dd, J = 6.3, 3.4 Hz, 2H), 7.96 (d, J = 8.3 Hz, 2H), 7.91 (dd, J = 6.3, 3.4 Hz, 2H), 7.83 (d, J = 7.9 Hz, 2H), 7.78 (d, J = 8.3 Hz, 4H), 7.67 (d, J = 6.3 Hz, 2H), 7.61 (d, J = 8.1 Hz, 4H).

^1H NMR (500 MHz, DMSO) δ 9.18 (s, 2H), 8.53 (d, J = 5.5 Hz, 2H), 8.25 (dd, J = 6.4, 3.4 Hz, 2H), 8.03 (d, J = 8.4 Hz, 2H), 7.96 (dd, J = 6.3, 3.4 Hz, 2H), 7.93 (d, J = 5.6 Hz, 2H), 7.87 (dd, J = 8.2, 7.2 Hz, 2H), 7.79 (d, J = 8.2 Hz, 4H), 7.67 (dd, J = 7.2, 0.9 Hz, 2H), 7.62 (d, J = 8.2 Hz, 4H).

^{13}C NMR (151 MHz, DMSO) δ 153.00, 149.97, 142.93, 140.80, 139.50, 138.80, 138.52, 136.00, 130.91, 130.55, 130.36, 129.91, 129.08, 128.72, 126.76, 125.91, 120.99.

HRMS-ESI $[C_{38}H_{24}N_4 + H]^+$ Calculated (m/z): 537.2074, found: 537.2031.

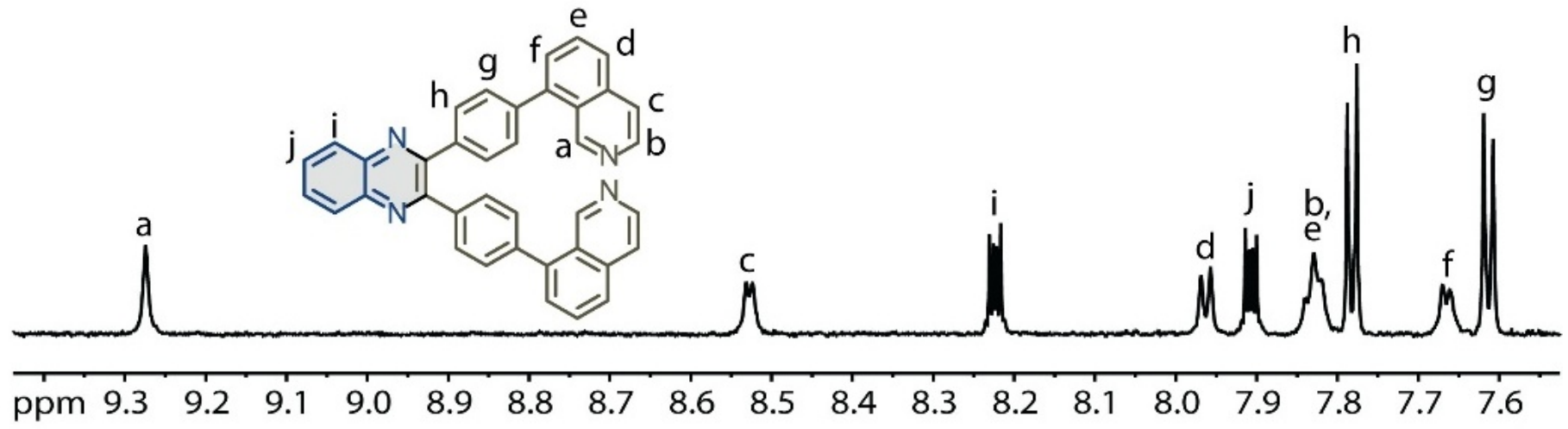

Figure S5. ^1H NMR spectrum (700 MHz, 298K, CD_3CN) of $\mathbf{L^B}$.

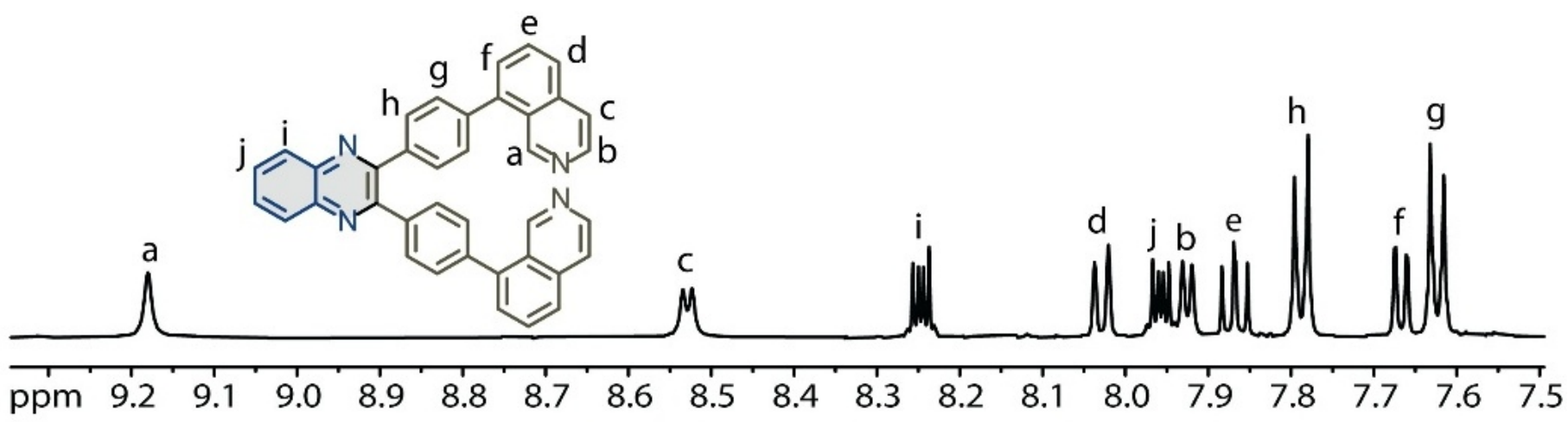

Figure S6. ^1H NMR spectrum (500 MHz, 298K, DMSO-d_6) of $\mathbf{L^B}$.

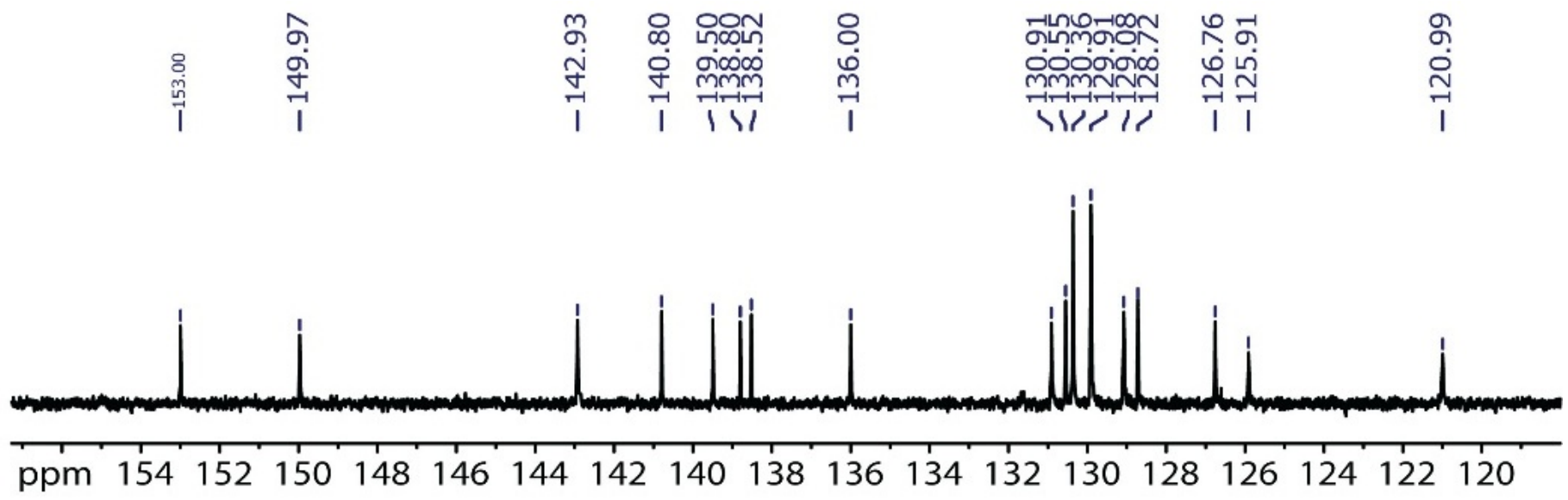

Figure S7. ^{13}C NMR spectrum (151 MHz, 298K, DMSO-d_6) of $\mathbf{L^{B1}}$.

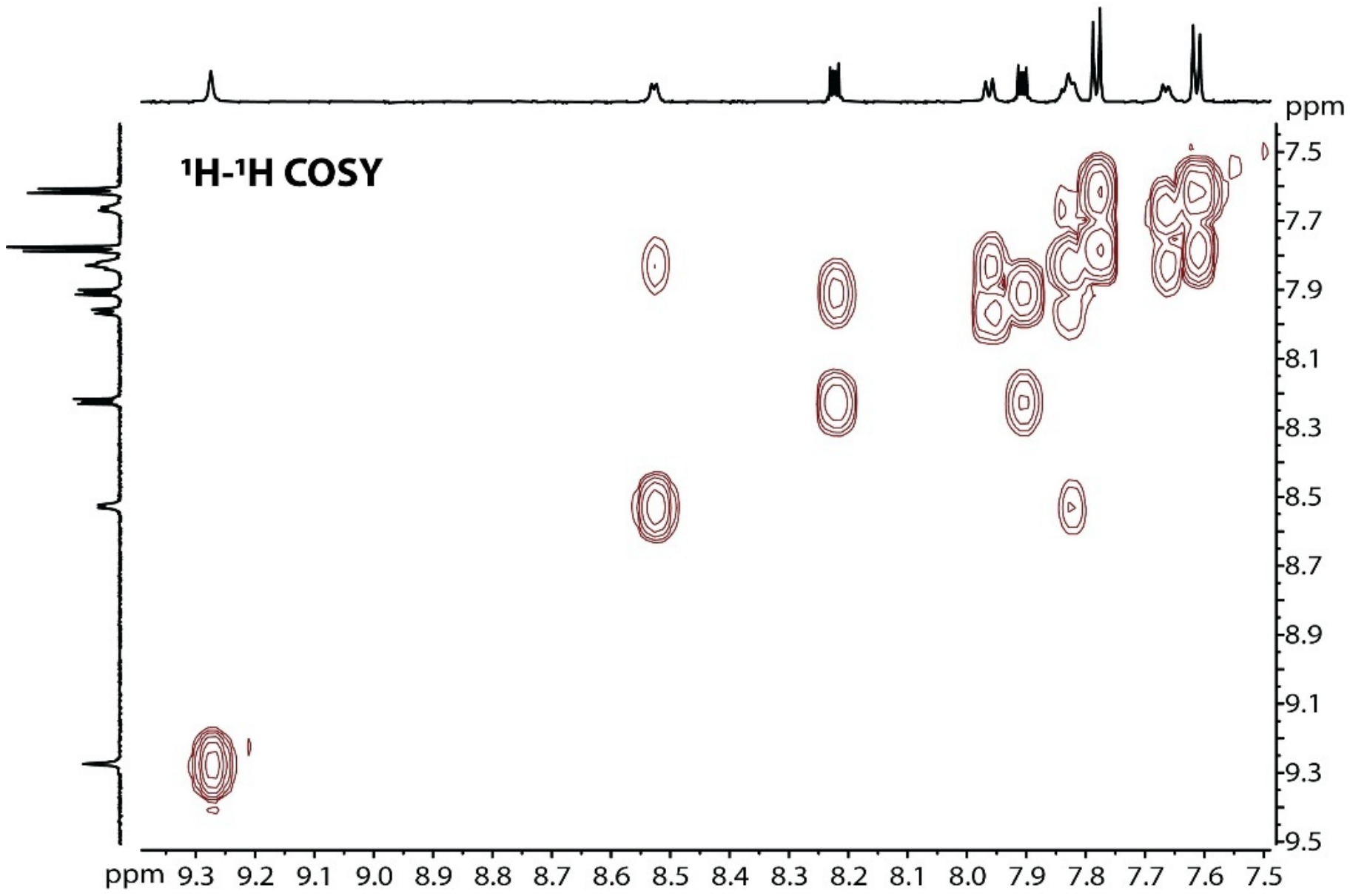

Figure S8. Partial ^1H-^1H COSY spectrum (700 MHz, 298K, CD_3CN) of **L^B^**.

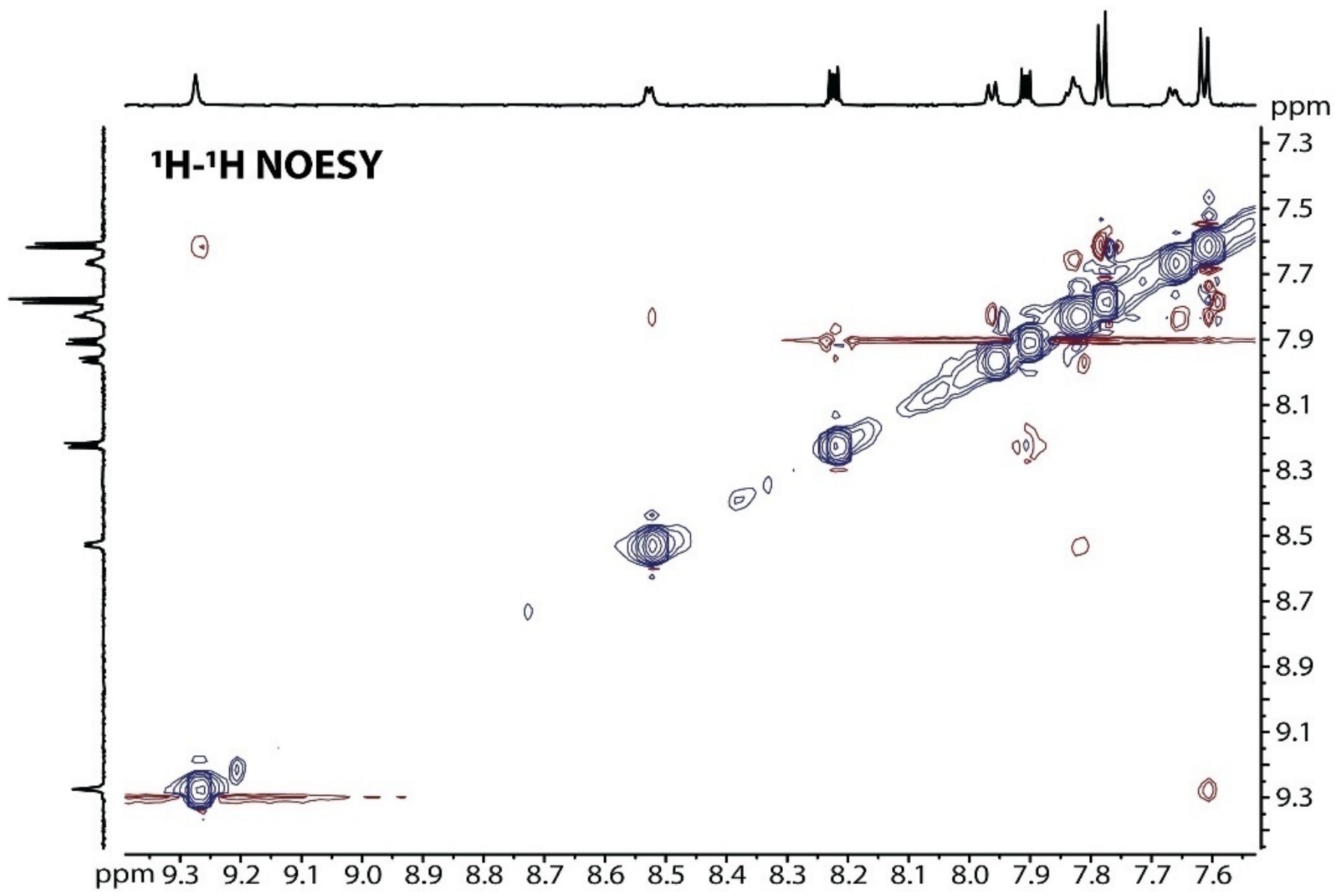

Figure S9. Partial ^1H-^1H NOESY spectrum (700 MHz, 298K, CD_3CN) of **L^B^**.

S3. Self-assembly of coordination cages

S3.1 Self-assembly of $[Pd_nL^A{}_{2n}](X)_{2n}$

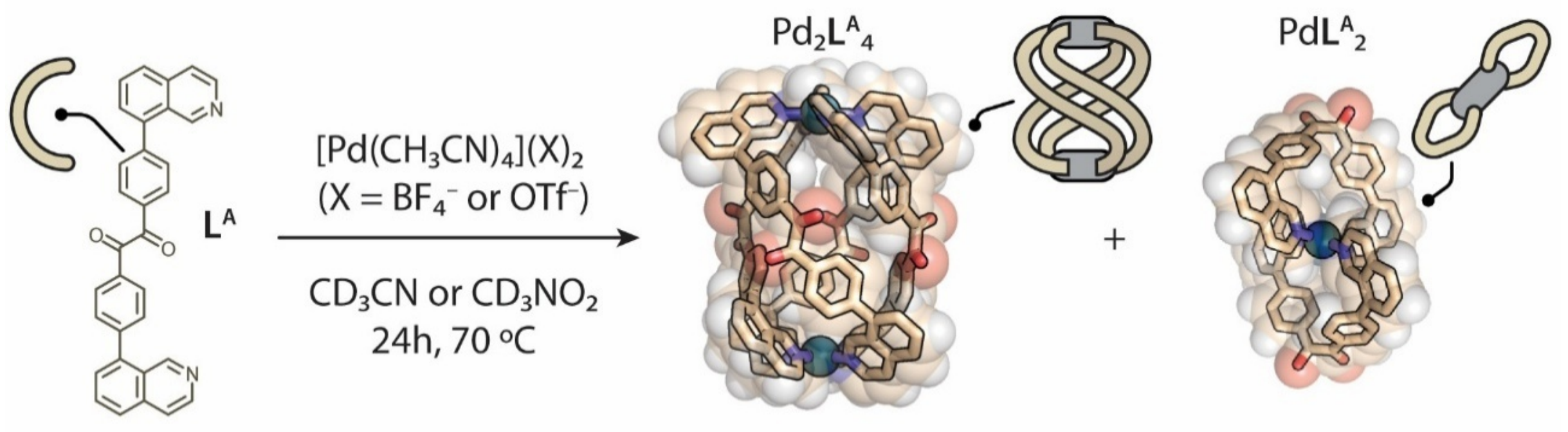

Scheme S3. Self-assembly of homoleptic $[Pd_n\mathbf{L^A}_{2n}](X)_{2n}$ (n = 1,2; X = BF_4^-, OTf^-) species.

A 3.11 mM suspension of $\mathbf{L^A}$ in CD_3CN or CD_3NO_2 (450 µl) was mixed with a 15 mM solution of $[Pd(CH_3CN)_4](X)_2$ (X = BF_4^-, OTf^-) in CD_3CN or CD_3NO_2 (50 µl) and stirred for 24 h at 70 °C to yield the $[Pd_n\mathbf{L^A}_{2n}](X)_{2n}$ (n = 1,2) species.

$[Pd_2\mathbf{L^A}_4](BF_4)_4$:

^{1}H NMR (600 MHz, CD_3CN, 343K) δ 8.59 (s, 8H), 8.38 (d, *J* = 6.4 Hz, 8H), 8.28 (d, *J* = 8.3 Hz, 8H), 8.23 (dd, *J* = 7.7, 7.2 Hz, 8H), 8.13 (d, *J* = 6.5 Hz, 8H), 8.00 (d, *J* = 6.0 Hz, 4H), 7.95 (d, *J* = 7.2 Hz, 8H), 7.15 (br, 16H, $Pd_2\mathbf{L^A}_4$), 6.90 (br, 16H).

^{1}H NMR (600 MHz, CD_3NO_2) δ 8.62 (s, 8H), 8.51 (d, J = 6.5 Hz, 8H), 8.38 – 8.31 (m, 16H), 8.21 (d, J = 6.5 Hz, 8H), 8.05 (dd, J = 6.6, 1.6 Hz, 8H), 7.13 (br, 16H), 6.94 (br, 16H).

^{13}C NMR (151 MHz, CD_3CN, 298K) δ 192.60, 154.85, 144.65, 144.09, 140.55, 137.85, 135.37, 132.19, 131.07, 128.66, 127.48, 126.18.

$[Pd\mathbf{L^A}_2](BF_4)_2$:

^{1}H NMR (500 MHz, CD_3CN) δ 9.52 (s, 4H), 8.31 (d, *J* = 8.1 Hz, 4H), 7.99 (m, 8H), 7.87 (dd, *J* = 6.0, 2.4 Hz, 8H), 7.85 (d, *J* = 6.6 Hz, 4H), 7.64 (d, *J* = 8.2 Hz, 8H), 7.47 (d, *J* = 6.6 Hz, 4H).

HRMS-ESI $[Pd_2(C_{32}H_{20}N_2O_2)_4]^{4+}$/$[Pd(C_{32}H_{20}N_2O_2)_2]^{2+}$ Calculated (m/z): 517.6050, found: 517.5897.

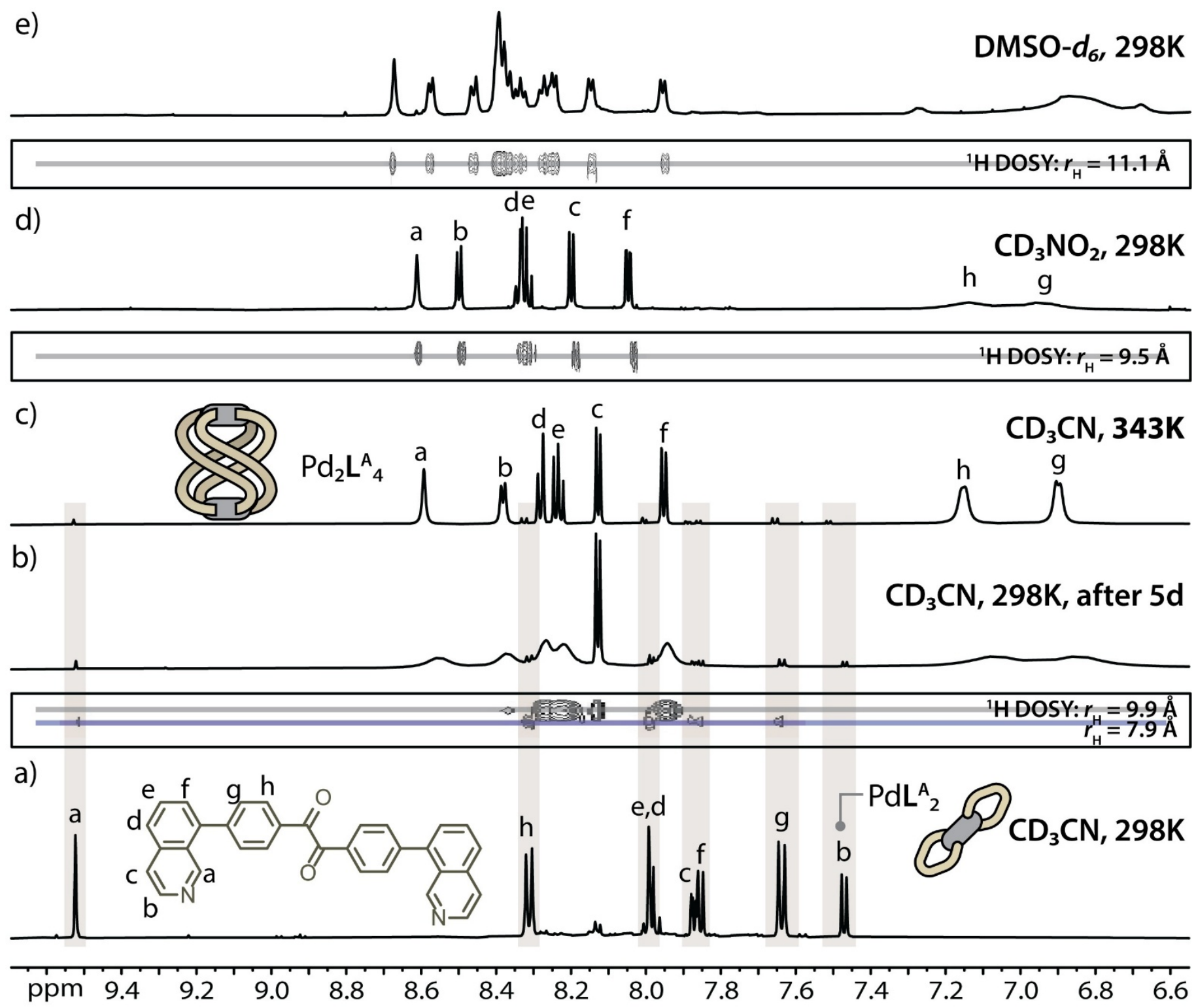

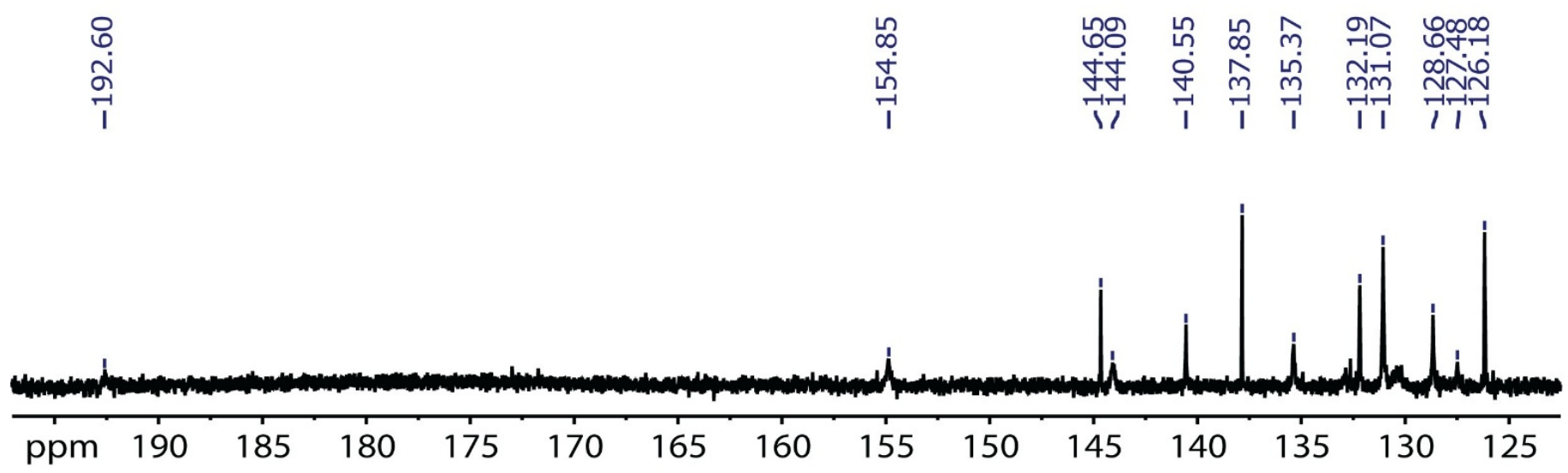

Figure S10. Stack of ^{1}H NMR spectra of (a) [Pd**L**$^{\mathbf{A}}$$_2$]$(BF_4)_2$ just after reaction start at 298K in CD_3CN, [Pd$_n$**L**$^{\mathbf{A}}$$_{2n}$]$(BF_4)_{2n}$ (n = 1,2) equilibrium mixture at (b) 298K (with corresponding ^{1}H DOSY traces) and (c) 70 °C in CD_3CN, equilibrium mixture at 298K in CD_3NO_2 (with corresponding ^{1}H DOSY traces), equilibrium mixture at 298K in DMSO-d_6 (with corresponding ^{1}H DOSY traces)

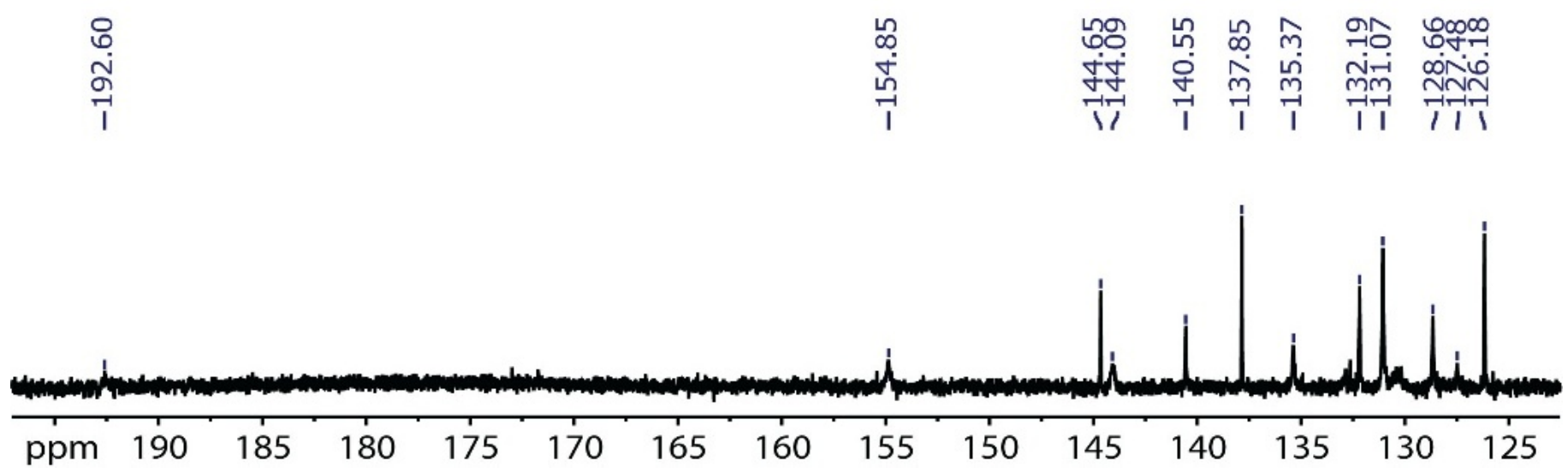

Figure S11. ^{13}C NMR spectrum (151 MHz, 298K, CD_3CN) of [Pd$_n$**L**$^{\mathbf{A}}$$_{2n}$]$(BF_4)_{2n}$ (n = 1,2) mixture.

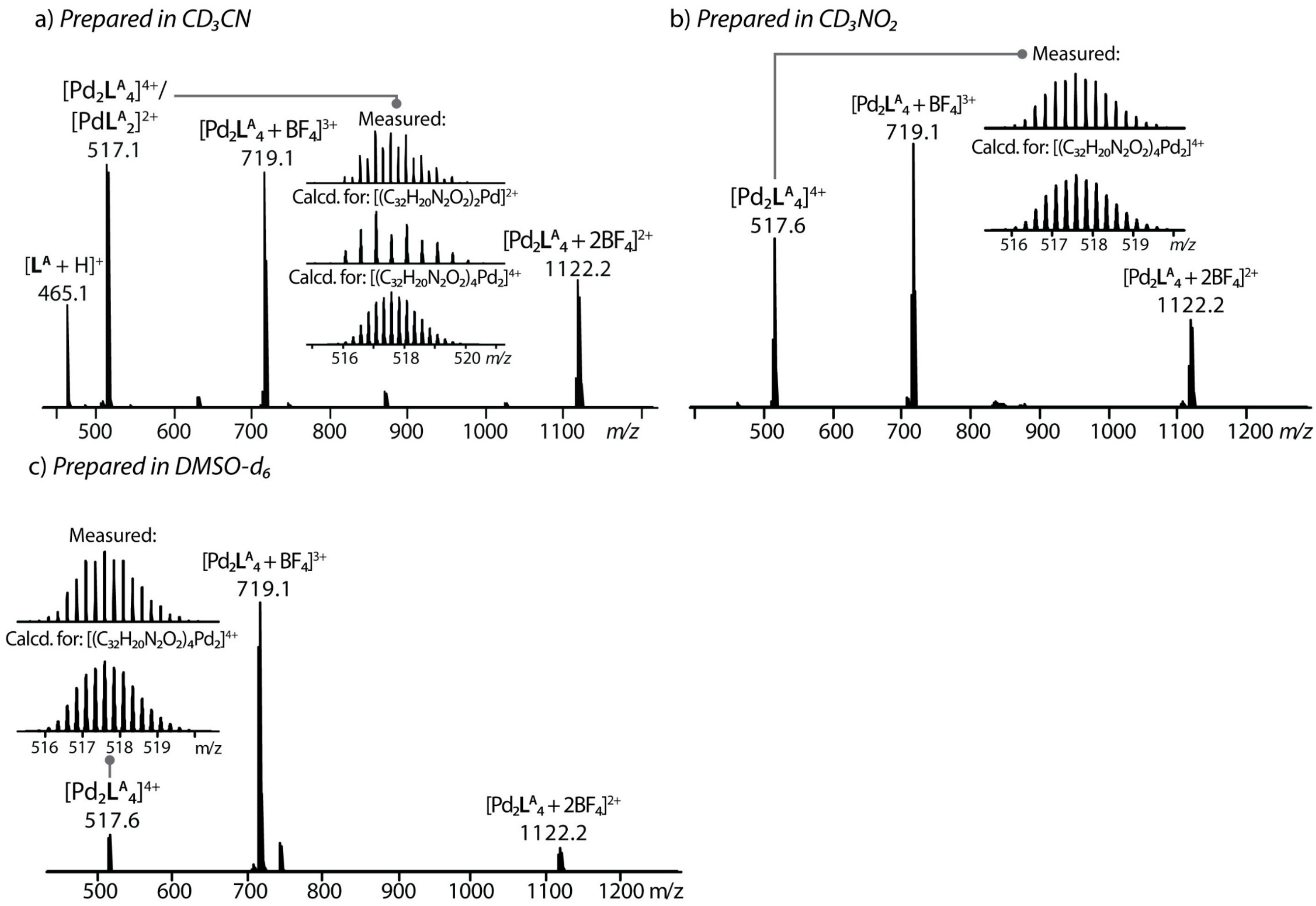

Figure S12. ESI-MS spectrum of homoleptic **L^A** assemblies prepared in different solvents.

$[Pd_2$**L**$^A{}_4](OTf)_4$:

^{1}H NMR (600 MHz, CD_3CN) δ 8.49 (s, 8H), 8.38 (d, *J* = 6.5 Hz, 8H), 8.31 (d, *J* = 8.3 Hz, 8H), 8.28 – 8.23 (m, 8H), 8.16 (d, *J* = 6.5 Hz, 8H), 7.99 (d, *J* = 7.2 Hz, 8H), 7.30 – 6.60 (br, 32H).

^{13}C NMR (151 MHz, CD3CN) δ 192.14, 155.02, 144.43, 144.31, 140.64, 137.93, 135.50, 132.82, 132.21, 131.00, 128.72, 127.34, 126.23.

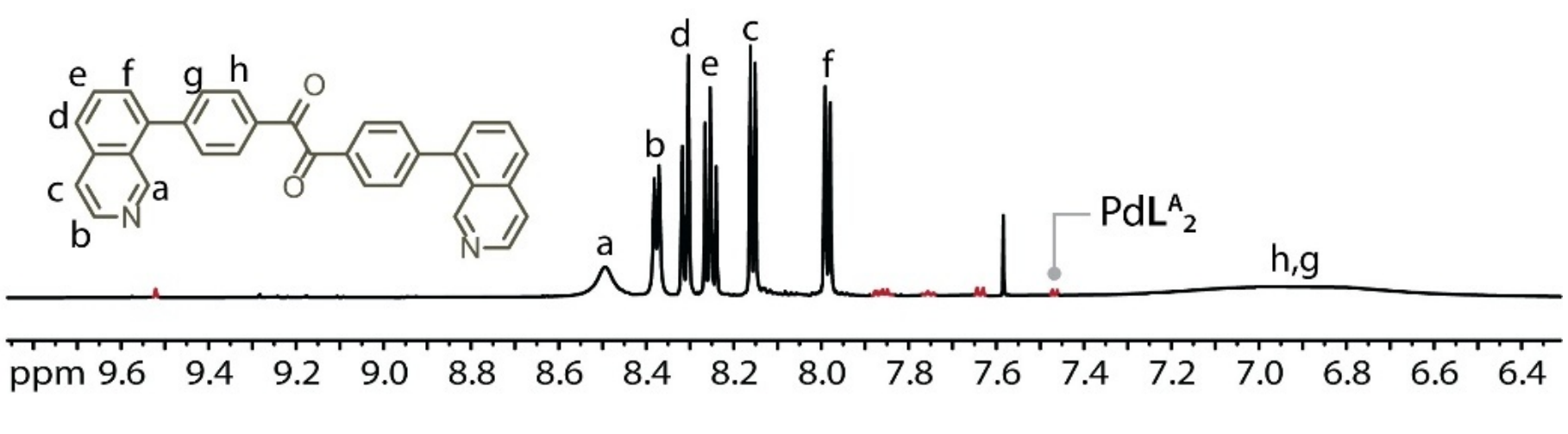

Figure S13. ^{1}H NMR spectrum (600 MHz, 298K, CD_3CN) of $[Pd_nL^A{}_{2n}](OTf)_{2n}$ (n = 1,2) mixture.

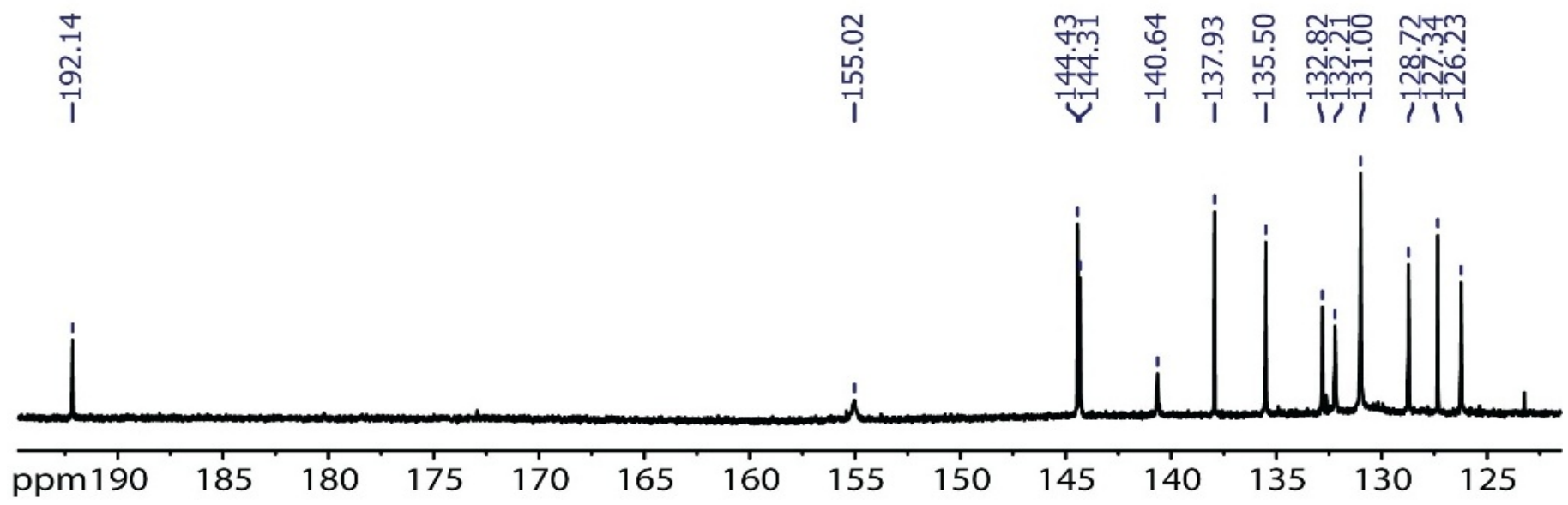

Figure S14. ^{13}C NMR spectrum (151 MHz, 298K, CD_3CN) of $[Pd_nL^A{}_{2n}](OTf)_{2n}$ (n = 1,2) mixture.

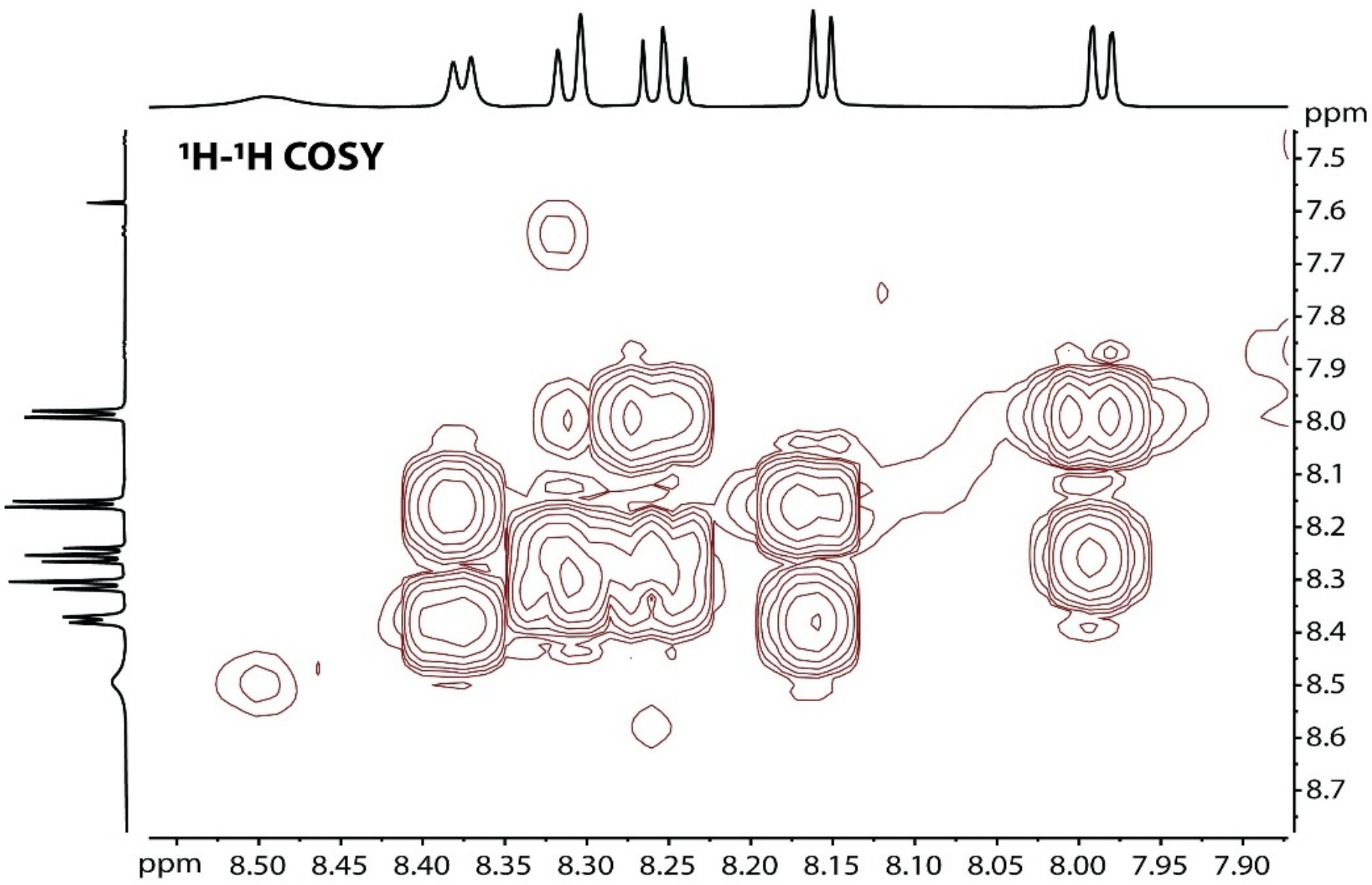

Figure S15. Partial ^1H-^1H COSY spectrum (600 MHz, 298K, CD_3CN) of $[Pd_2\mathbf{L^A}_4](OTf)_4$.

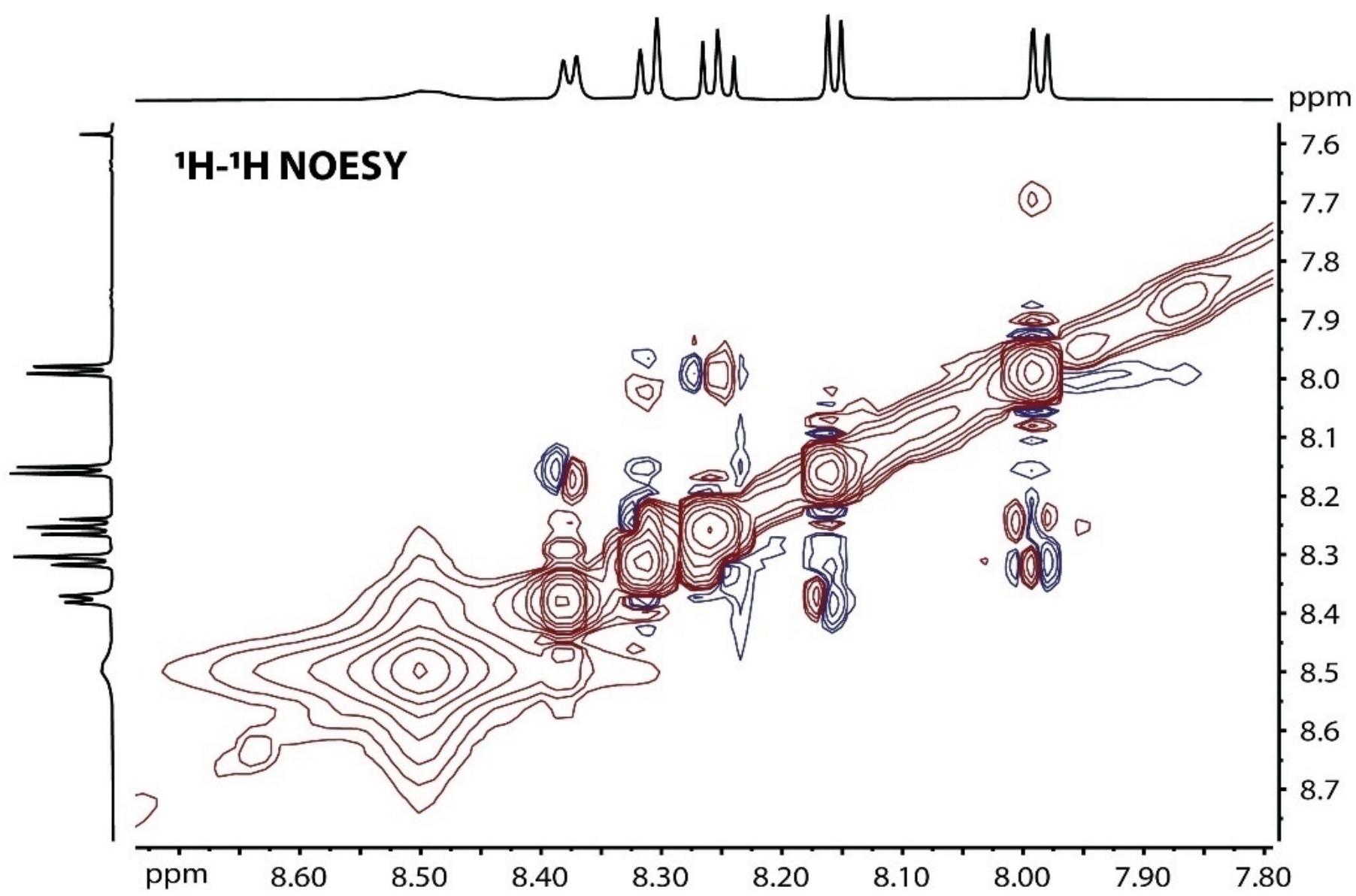

Figure S16. Partial ^1H-^1H NOESY spectrum (600 MHz, 298K, CD_3CN) of $[Pd_2\mathbf{L^A}_4](OTf)_4$.

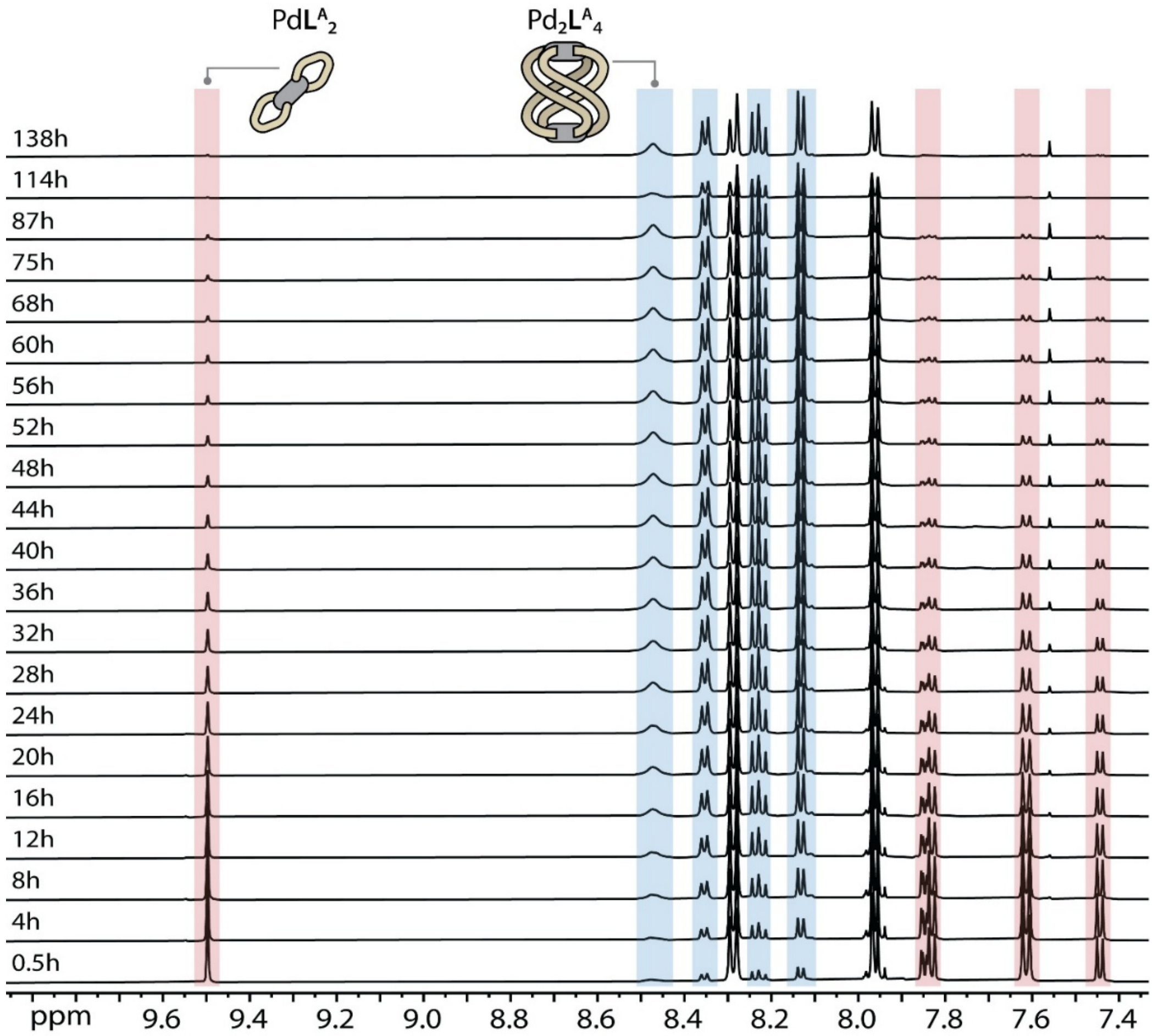

Figure S17. Monitoring of dimerization of [PdL^A_2]OTf_2 to [$Pd_2L^A_4$]$(OTf)_4$ by ^{1}H NMR at RT (500 MHz, 298K, CD_3CN).

S3.2 Self-assembly of PdL^B_2

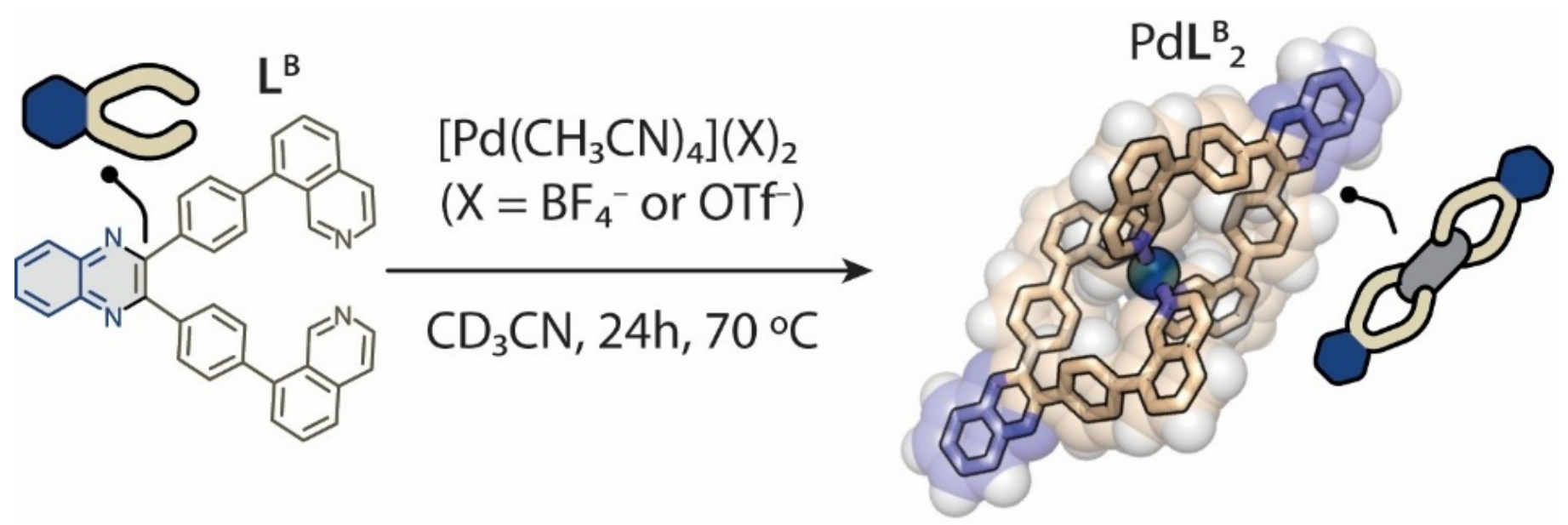

Scheme S4. Self-assembly of homoleptic PdL^B_2 complex.

A 3.11 mM suspension of L^B in CD_3CN or CD_3NO_2 (450 µl) was mixed with a 15 mM solution of [$Pd(CH_3CN)_4$]$(BF_4)_2$ in CD_3CN (50 µl) and stirred for 18 h at 70 °C to yield the PdL^B_2 species.

[PdL^A_2]$(BF_4)_2$:

^{1}H NMR (600 MHz, CD_3CN) δ 10.33 (s, 4H), 8.28 (dd, *J* = 6.3, 3.4 Hz, 4H), 8.18 (d, *J* = 8.3 Hz, 8H), 8.01 (dd, *J* = 8.2, 6.9 Hz, 4H), 7.99 – 7.93 (m, 16H), 7.89 (d, *J* = 6.5 Hz, 4H), 7.82 (d, *J* = 6.5 Hz, 4H), 7.74 (d, *J* = 8.3 Hz, 8H).

^{1}H NMR (600 MHz, CD_3NO_2) δ 10.50 (s, 4H), 8.31 (dd, *J* = 6.3, 3.4 Hz, 4H), 8.28 (d, *J* = 8.3 Hz, 8H), 8.11 – 8.05 (m, 4H), 8.01 (td, *J* = 6.8, 3.8 Hz, 16H), 7.96 (d, *J* = 6.4 Hz, 4H), 7.94 (d, *J* = 6.6 Hz, 4H), 7.87 (d, *J* = 8.4 Hz, 8H).

^{13}C NMR (151 MHz, CD_3NO_2) δ 155.85, 154.17, 142.57, 142.27, 142.17, 141.05, 139.05, 138.66, 135.83, 132.25, 132.18, 132.03, 131.94, 130.46, 128.40, 127.85, 126.88.

HRMS-ESI $[Pd(C_{38}H_{24}N_4)_2]^{2+}$ Calculated (m/z): 589.1525, found: 589.1174.

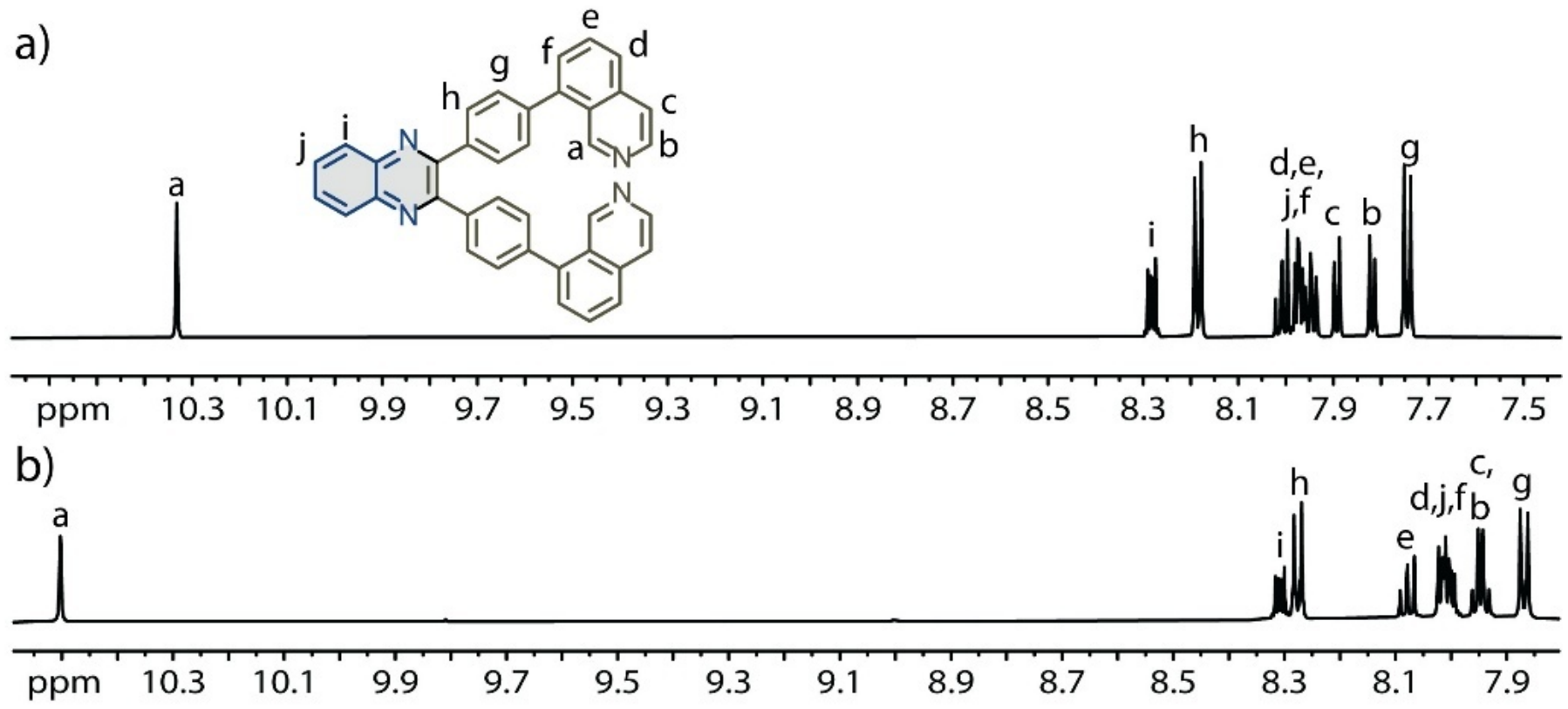

Figure S18. Stack of ^{1}H NMR spectra (600 MHz) of $[Pd\mathbf{L^B}_2](BF_4)_2$ in (a) CD_3CN and (a) CD_3NO_2.

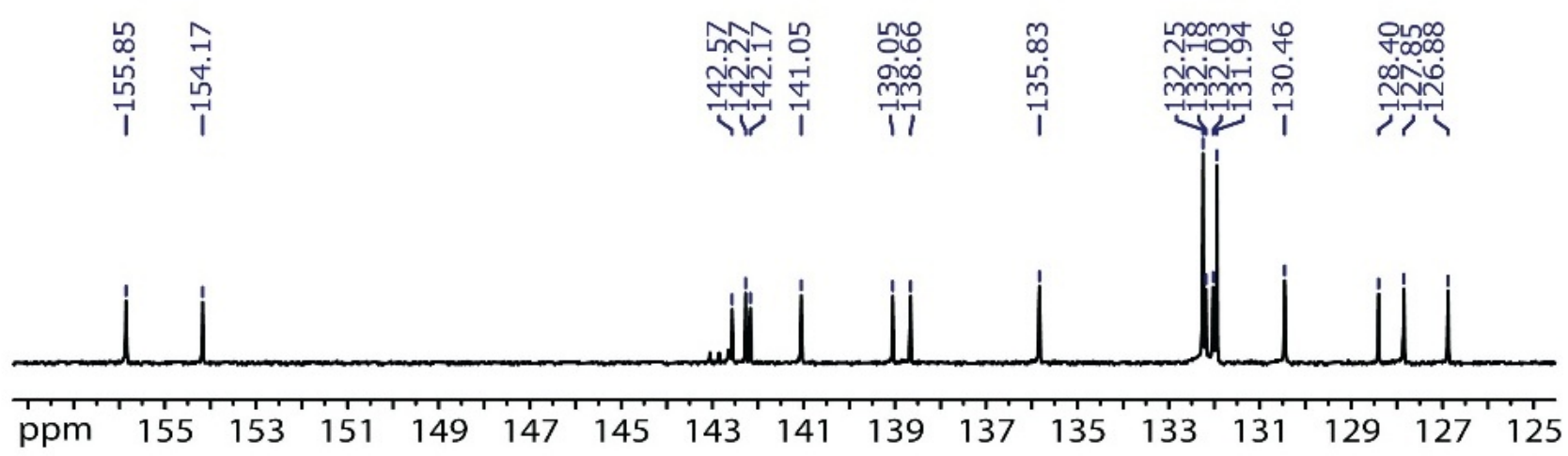

Figure S19. ^{13}C NMR spectrum (151 MHz, 298K, CD_3NO_2) of $[Pd\mathbf{L^B}_2](BF_4)_2$.

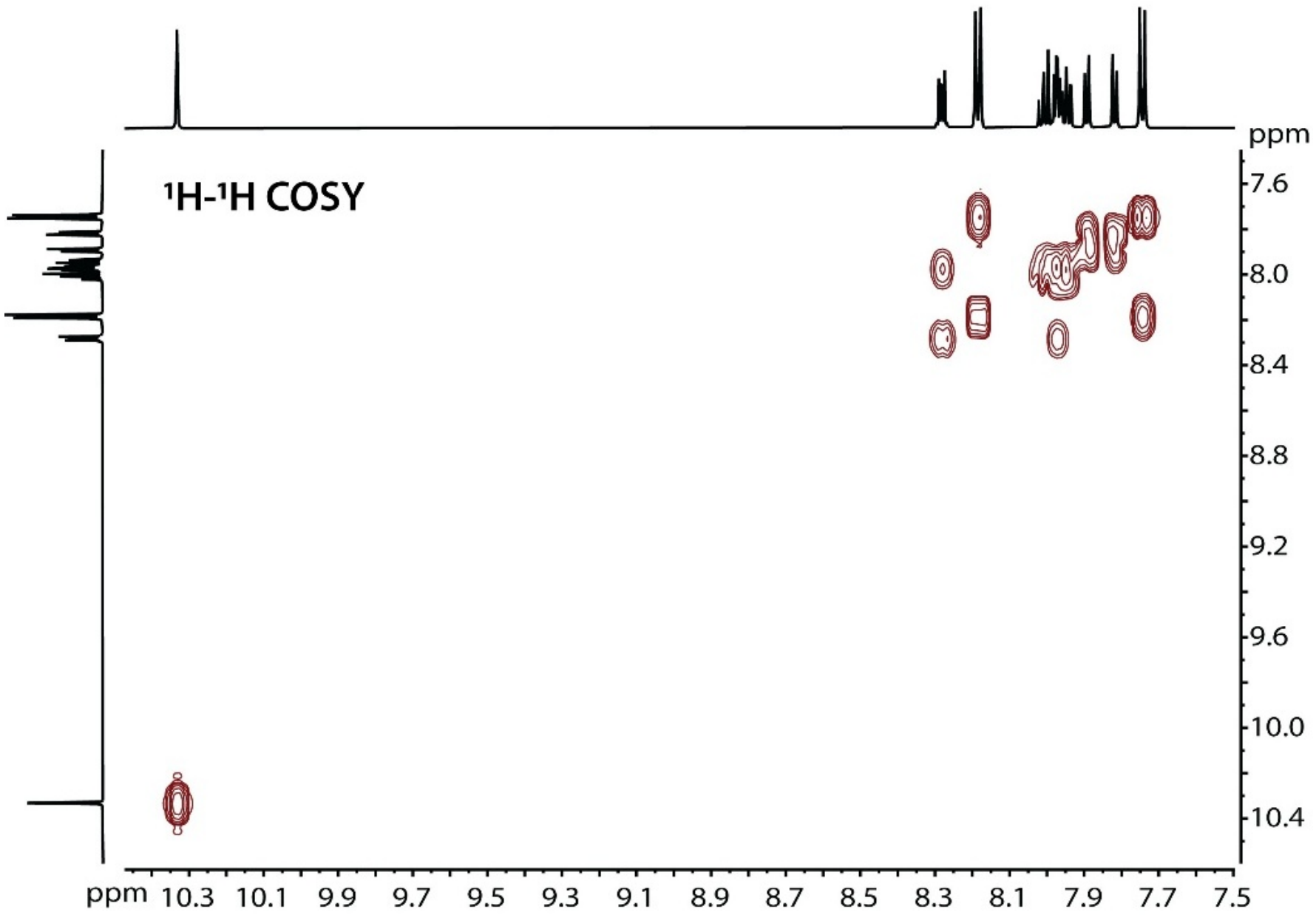

Figure S20. Partial ^{1}H-^{1}H COSY spectrum (600 MHz, 298K, CD_3CN) of [Pd**L**$^{\mathbf{B}}_{2}$]$(BF_4)_2$.

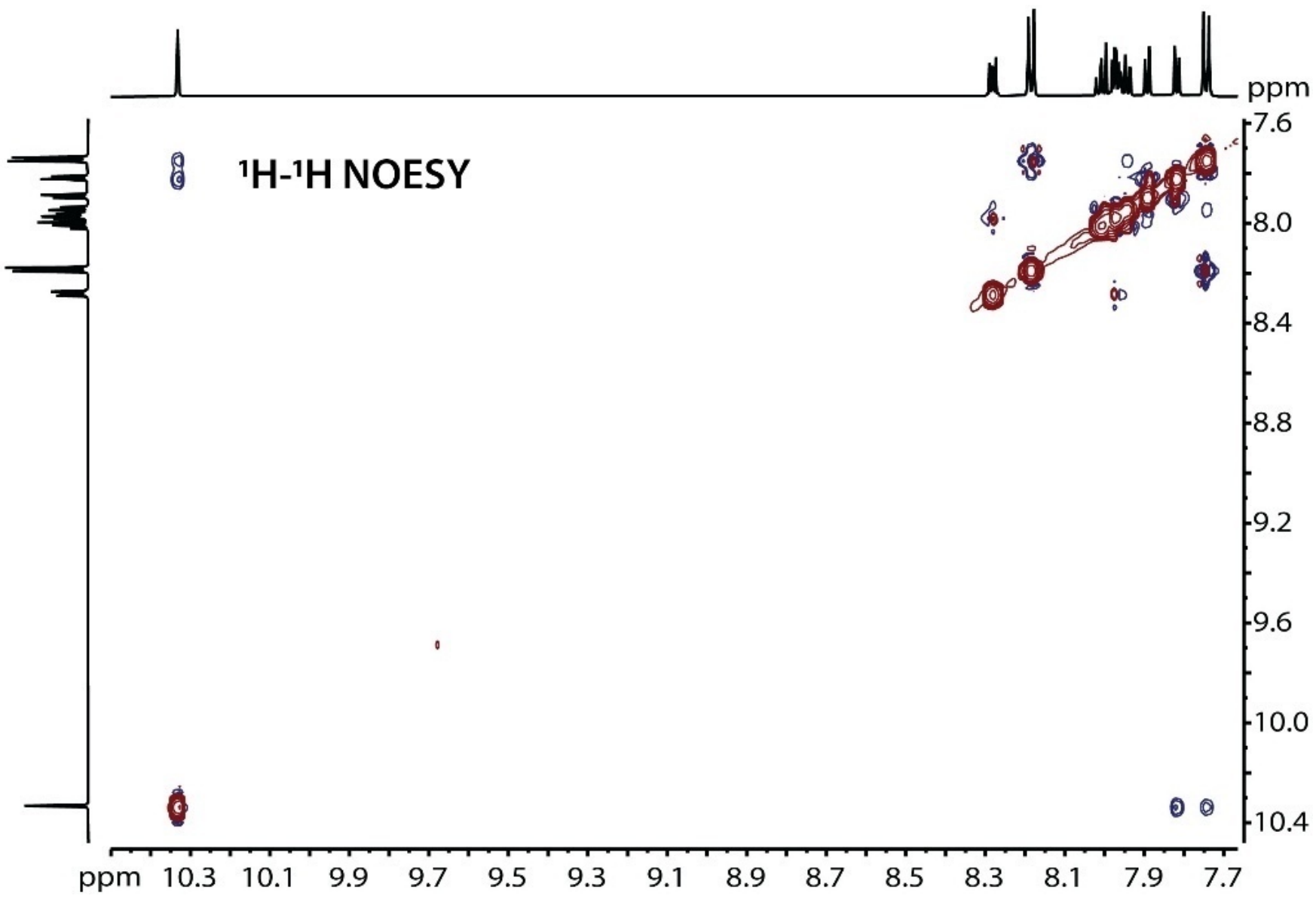

Figure S21. Partial ^{1}H-^{1}H NOESY spectrum (600 MHz, 298K, CD_3CN) of [Pd**L**$^{\mathbf{B}}_{2}$]$(BF_4)_2$.

S3.3 Self-assembly of $Pd_2L^A{}_2L^C{}_2$

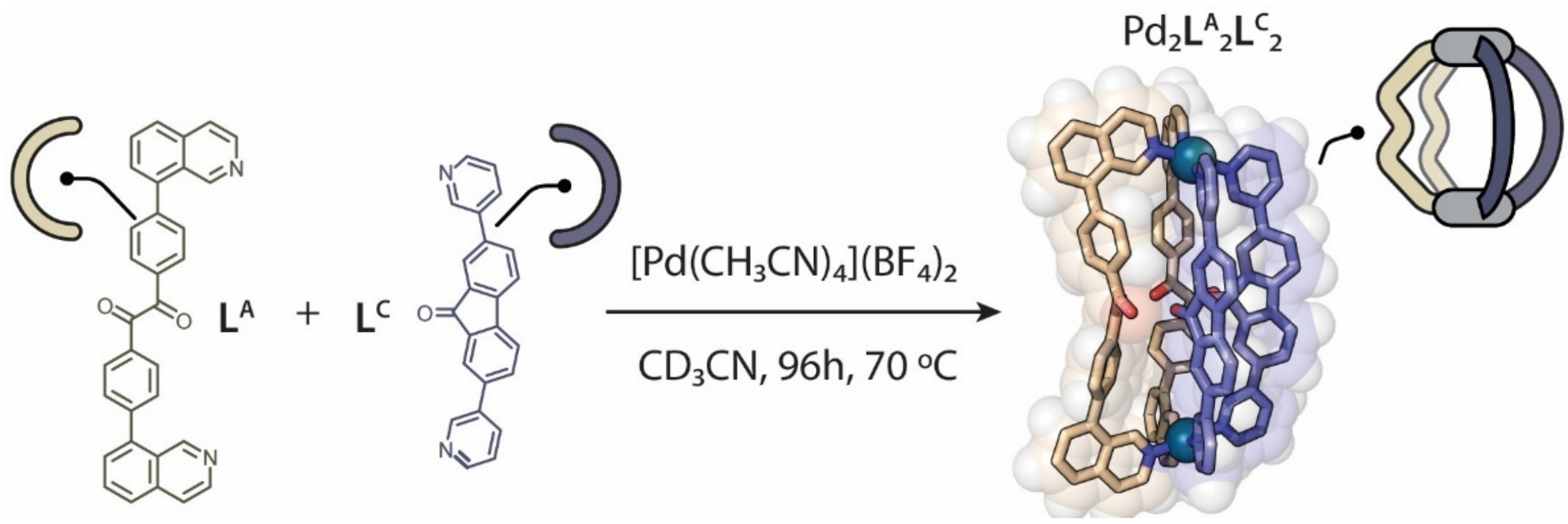

Scheme S5. Self-assembly of heteroleptic Pd$_2$**L**$^{A}_2$**L**$^{C}_2$ cage.

To a 3.11 mM solution of **L**^{A} in CD_3CN (225 µl) and a 3.11 mM solution of **L**^{C} in CD_3CN (225 µl) was added a stock solution of $[Pd(CH_3CN)_4](BF_4)_2$ (15 mM, 50 µl). The mixture was heated for 96h at 70 °C to yield the Pd$_2$**L**$^{A}_2$**L**$^{C}_2$ cage solution.

^{1}H NMR (600 MHz, CD_3CN) δ 9.57 (s, 4H), 9.50 (s, 4H), 9.34 (d, *J* = 6.6 Hz, 4H), 8.97 (dd, *J* = 5.8, 0.8 Hz, 4H), 8.26 (d, *J* = 6.6 Hz, 4H), 8.24 (d, *J* = 7.8 Hz, 4H), 8.12 (m, 12H), 7.99 (d, *J* = 7.0 Hz, 4H), 7.97 (dd, *J* = 7.4, 6.1 Hz, 4H), 7.90 (dd, *J* = 7.6, 1.7 Hz, 4H), 7.78 (s, 4H), 7.71 (dd, *J* = 6.9, 0.7 Hz, 1H), 7.59 (dd, *J* = 7.7, 5.9 Hz, 4H), 7.51 (s, 8H).

^{13}C NMR (151 MHz, CD_3CN) δ 193.23, 190.26, 154.77, 151.38, 148.25, 145.90, 144.31, 142.01, 140.74, 140.39, 139.50, 137.77, 137.40, 137.10, 135.50, 135.10, 134.73, 132.34, 132.18, 131.29, 128.50, 128.24, 128.01, 126.55, 124.04, 122.39.

HRMS-ESI $[Pd_2(C_{32}H_{20}N_2O_2)_2(C_{23}H_{14}N_2O)_2]^{4+}$ Calculated (m/z): 452.5832, found: 452.5839.

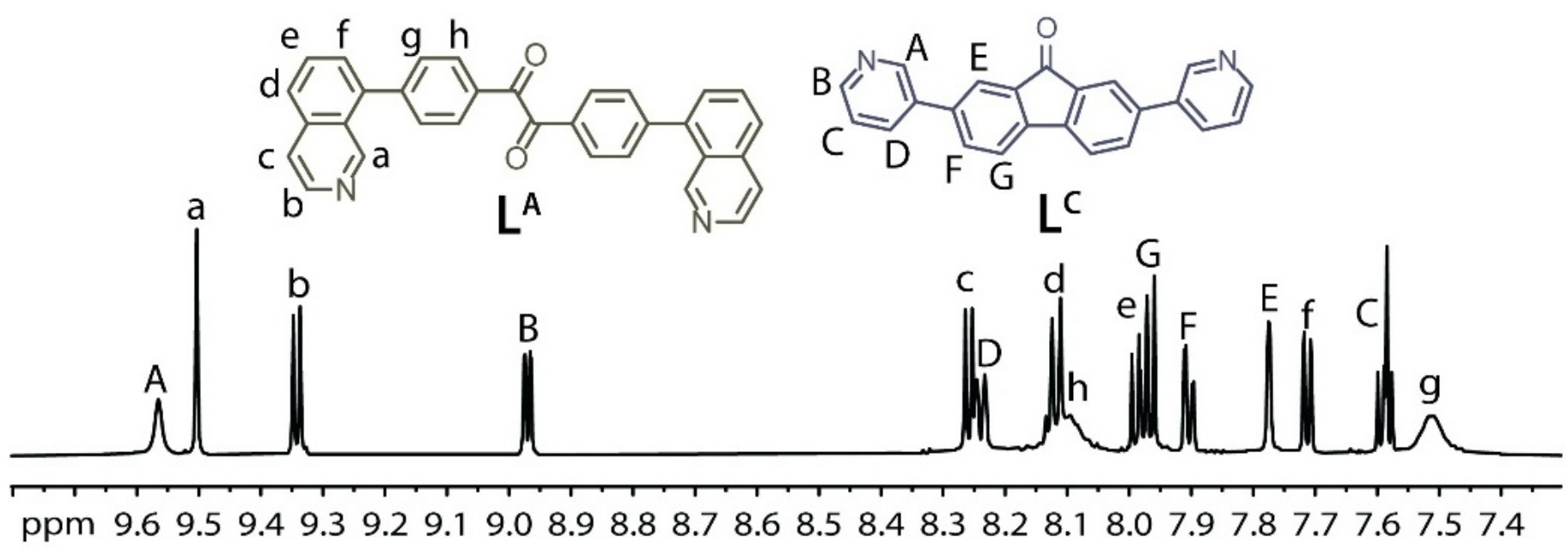

Figure S22. ^{1}H NMR spectrum (600 MHz, 298K, CD_3CN) of Pd_2**L**$^{A}{}_2$**L**$^{C}{}_2$.

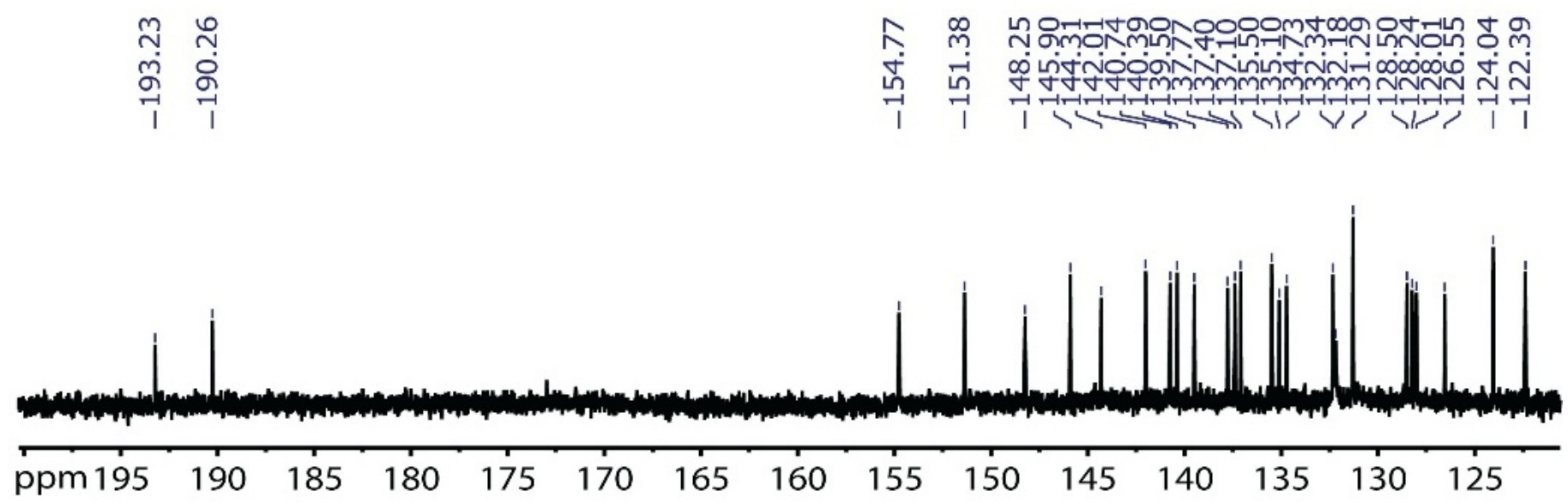

Figure S23. ^{13}C NMR spectrum (151 MHz, 298K, CD_3CN) of Pd_2**L**$^{A}{}_2$**L**$^{C}{}_2$.

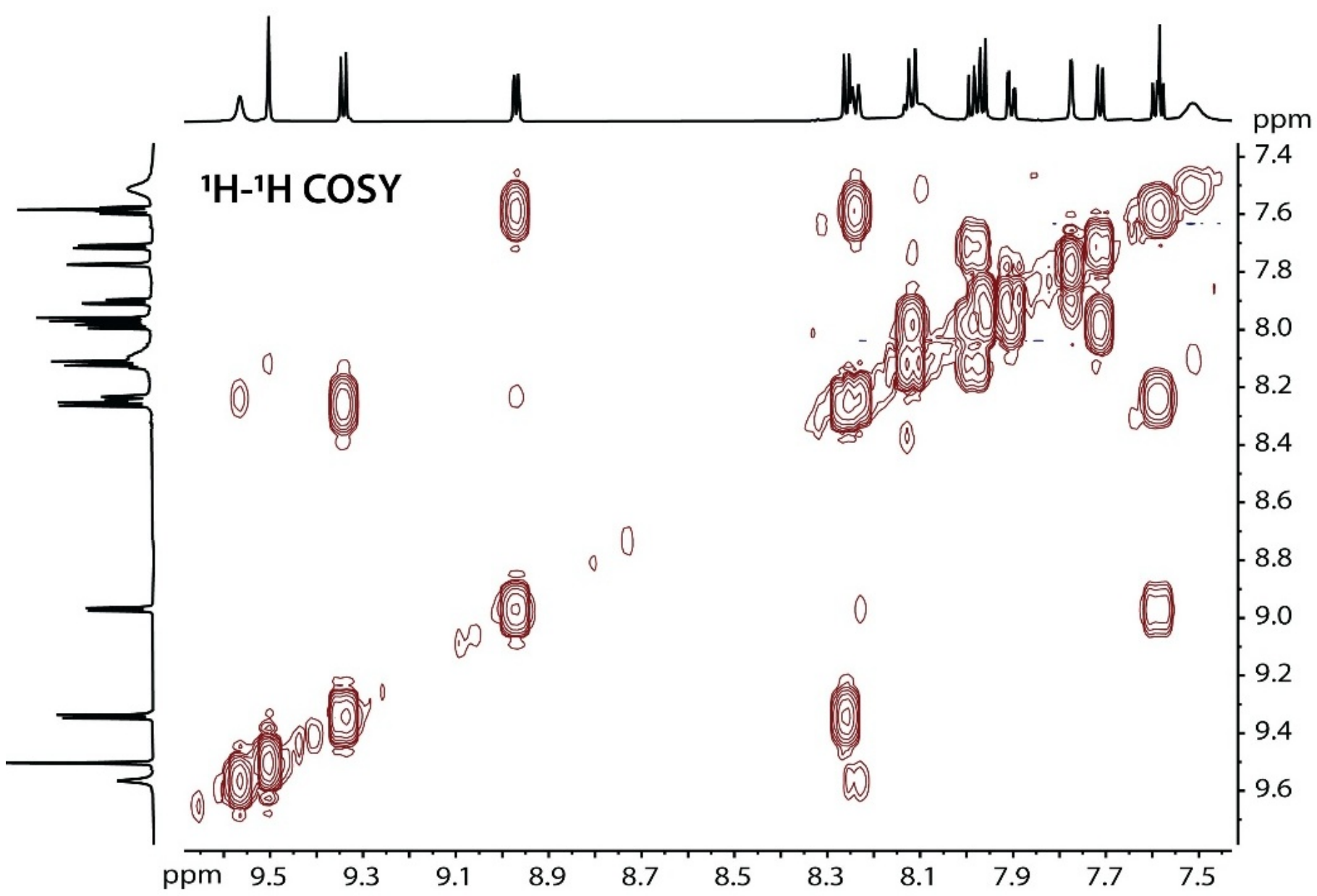

Figure S24. Partial ^{1}H-^{1}H COSY spectrum (600 MHz, 298K, CD_3CN) of Pd_2**L**$^{A}{}_2$**L**$^{C}{}_2$.

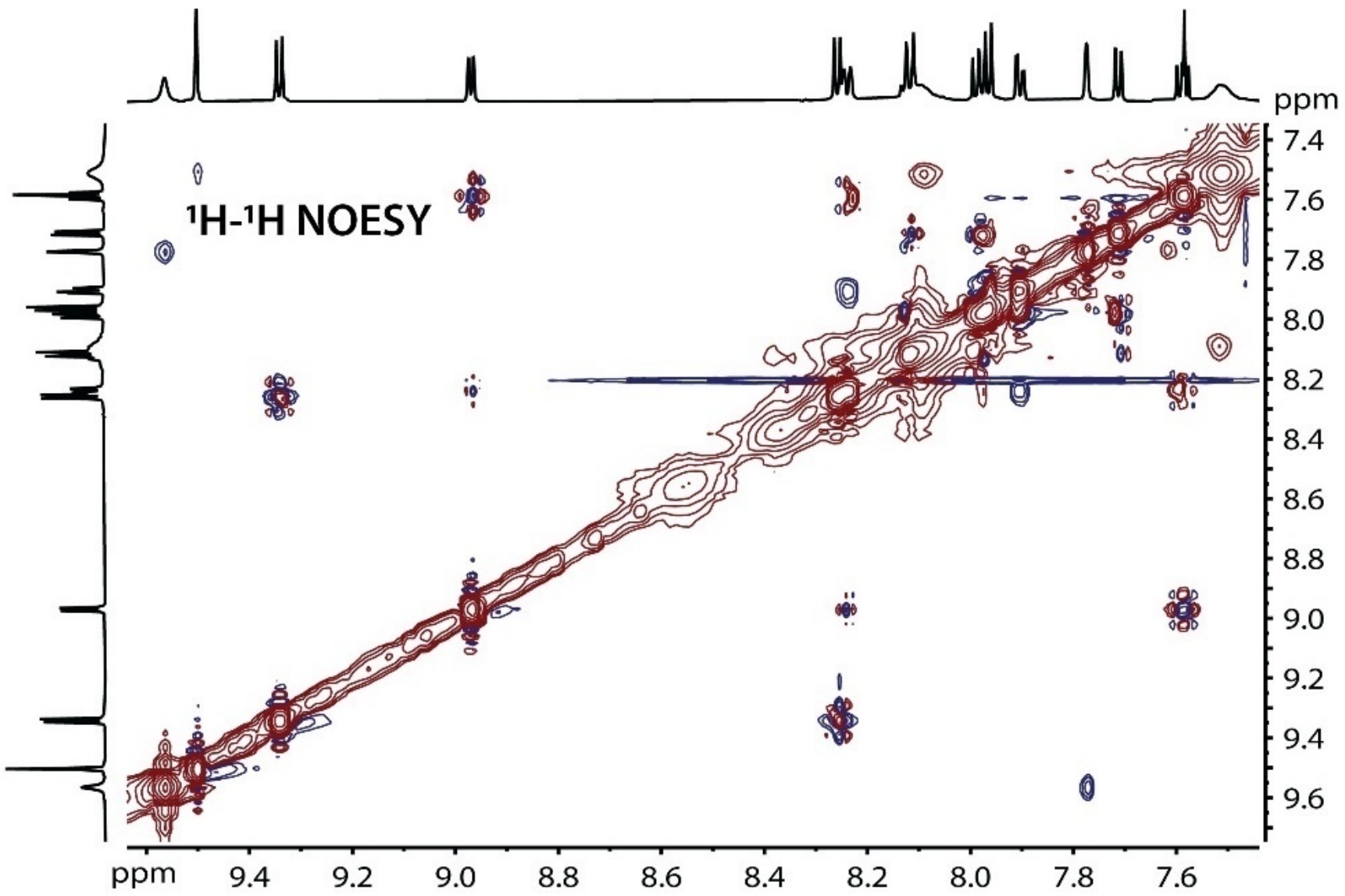

Figure S25. Partial ^{1}H-^{1}H NOESY spectrum (600 MHz, 298K, CD_3CN) of $Pd_2\mathbf{L}^{A}{}_2\mathbf{L}^{C}{}_2$.

S3.4 Self-assembly of $Pd_3\mathbf{L}^{A}{}_2\mathbf{L}^{D}{}_4$

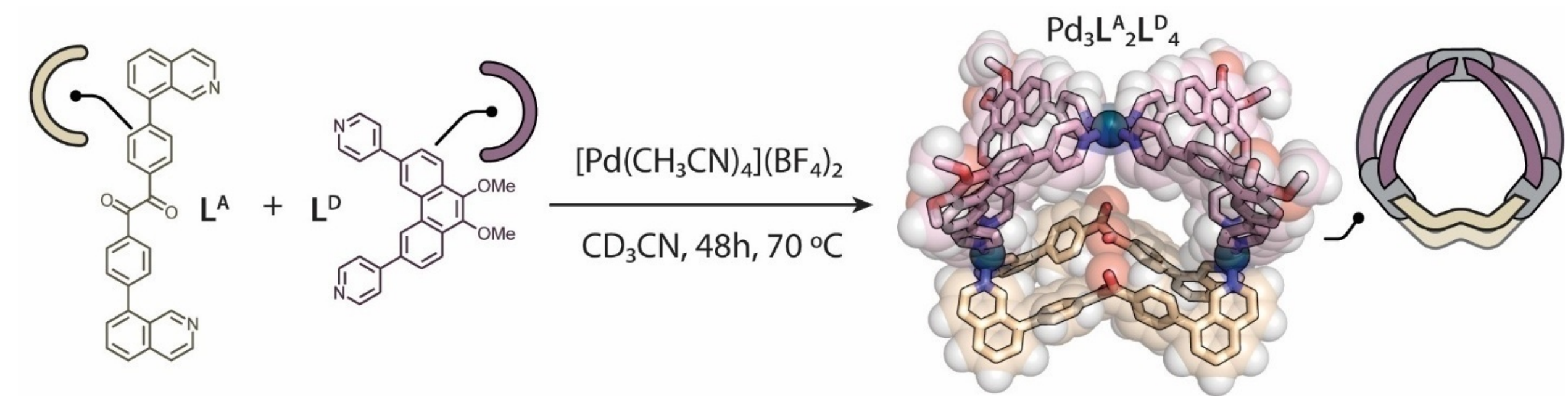

Scheme S6. Self-assembly of heteroleptic cage $Pd_3\mathbf{L}^{A}{}_2\mathbf{L}^{D}{}_4$.

To a 3.11 mM solution of $\mathbf{L}^{A}$ in CD_3CN (225 µl) and a 3.11 mM solution of $\mathbf{L}^{D}$ in CD_3CN (450 µl) was added a stock solution of $[Pd(CH_3CN)_4](BF_4)_2$ (15 mM, 50 µl). The mixture was heated for 48 h at 70 °C to yield the $Pd_3\mathbf{L}^{A}{}_2\mathbf{L}^{D}{}_4$ cage solution.

^{1}H NMR (600 MHz, CD_3CN) δ 9.72 (s, 1H), 9.43 – 9.38 (m, 2H), 9.30 (d, *J* = 6.5 Hz, 1H), 9.20 (d, *J* = 1.6 Hz, 2H), 9.19 (s, 1H), 9.18(s, 1H), 8.50 (d, *J* = 7.8 Hz, 2H), 8.36 (d, *J* = 8.6 Hz, 1H), 8.33 (d, *J* = 8.8 Hz, 1H), 8.30 – 8.27 (m, 2H), 8.21 (d, *J* = 6.9 Hz, 1H), 8.20 – 8.18 (m, 2H), 8.12 – 8.10 (m, 1H), 8.07 (d, *J* = 8.7 Hz, 1H), 8.01 – 7.99 (m, 1H), 7.99 – 7.97 (m, 1H), 7.81 (d, *J* = 7.9 Hz, 2H), 7.77 (d, *J* = 6.5 Hz, 1H).

^{13}C NMR (151 MHz, CD_3CN) δ 194.73, 172.96, 154.79, 152.42, 152.33, 152.20, 151.88, 145.84, 145.83, 145.61, 142.35, 140.81, 137.79, 137.76, 134.58, 133.51, 133.45, 132.32, 131.96, 131.81, 131.01, 129.62, 129.60, 128.44, 128.27, 126.61, 126.55, 126.37, 126.12, 125.92, 124.63, 124.58, 123.62, 123.59, 61.75, 61.74

HRMS-ESI $[Pd_3(C_{32}H_{20}N_2O_2)_2(C_{26}H_{20}N_2O_2)_4 + 3BF_4^{-}]^{3+}$ Calculated (m/z): 1025.8808, found: 1025.8755

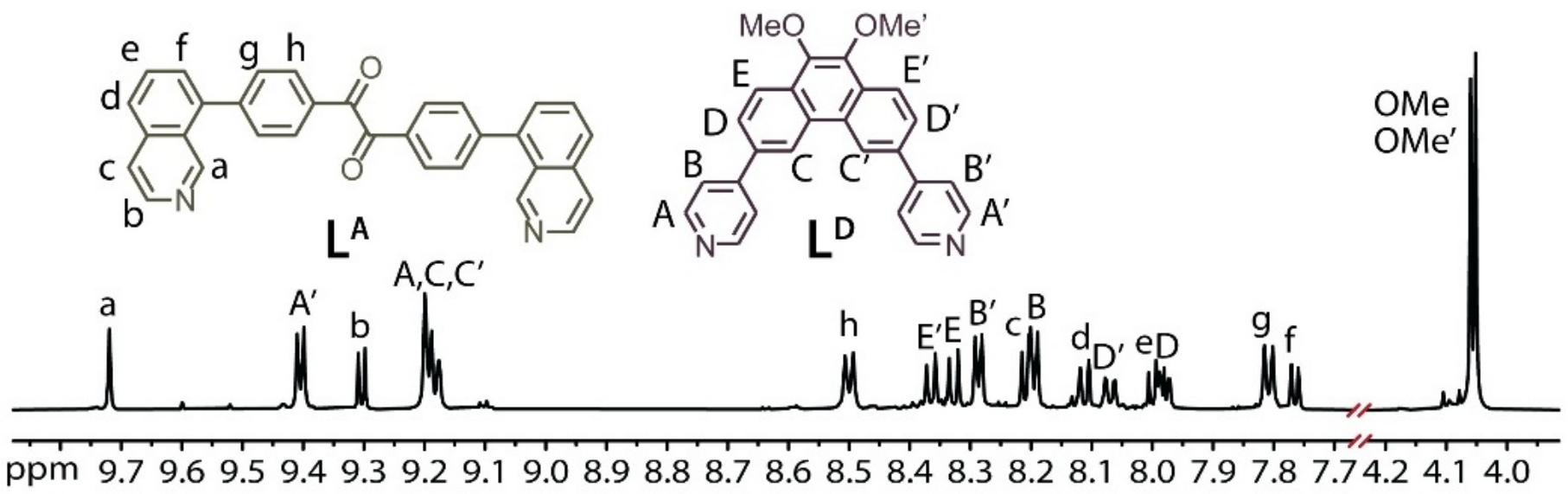

Figure S26. ^{1}H NMR spectrum (600 MHz, 298K, CD_3CN) of $Pd_3\mathbf{L^A}_2\mathbf{L^D}_4$.

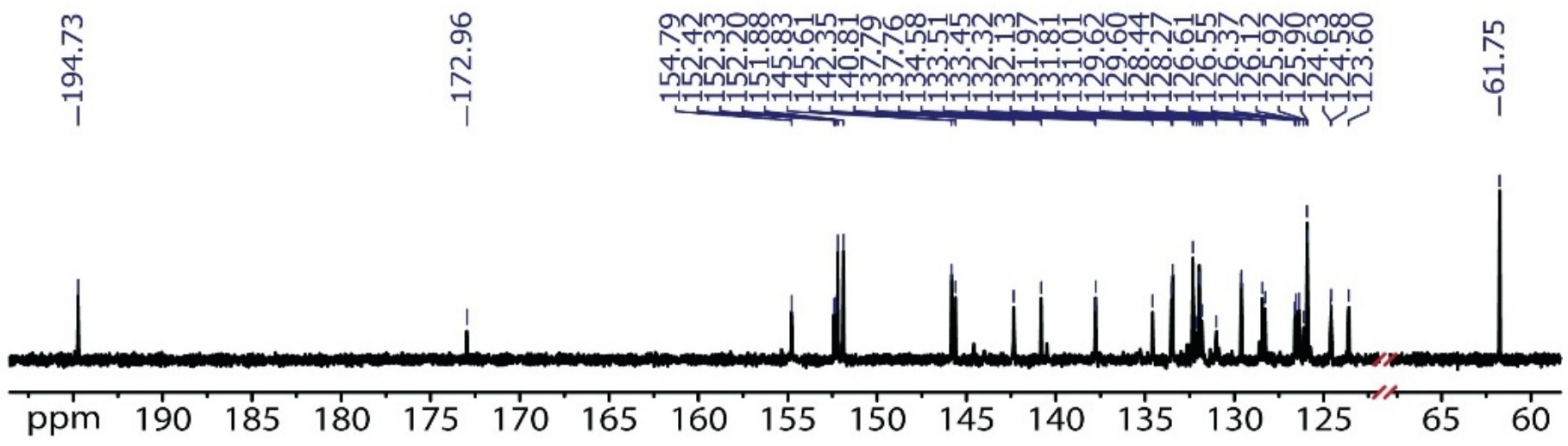

Figure S27. ^{13}C NMR spectrum (150 MHz, 298K, CD_3CN) of $Pd_3\mathbf{L^A}_2\mathbf{L^C}_4$.

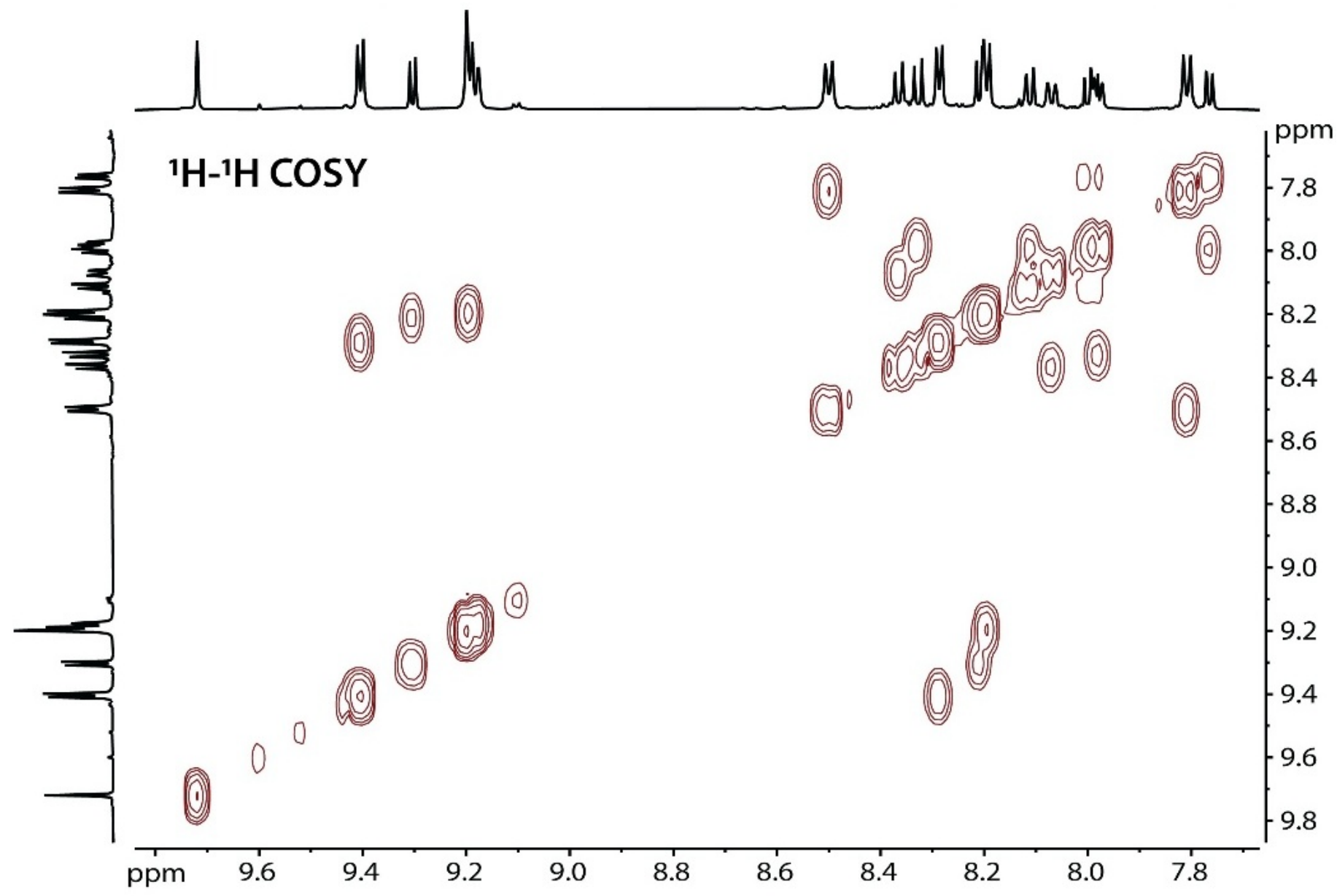

Figure S28. Partial ^{1}H-^{1}H COSY spectrum (600 MHz, 298K, CD_3CN) of $Pd_3\mathbf{L^A}_2\mathbf{L^D}_4$.

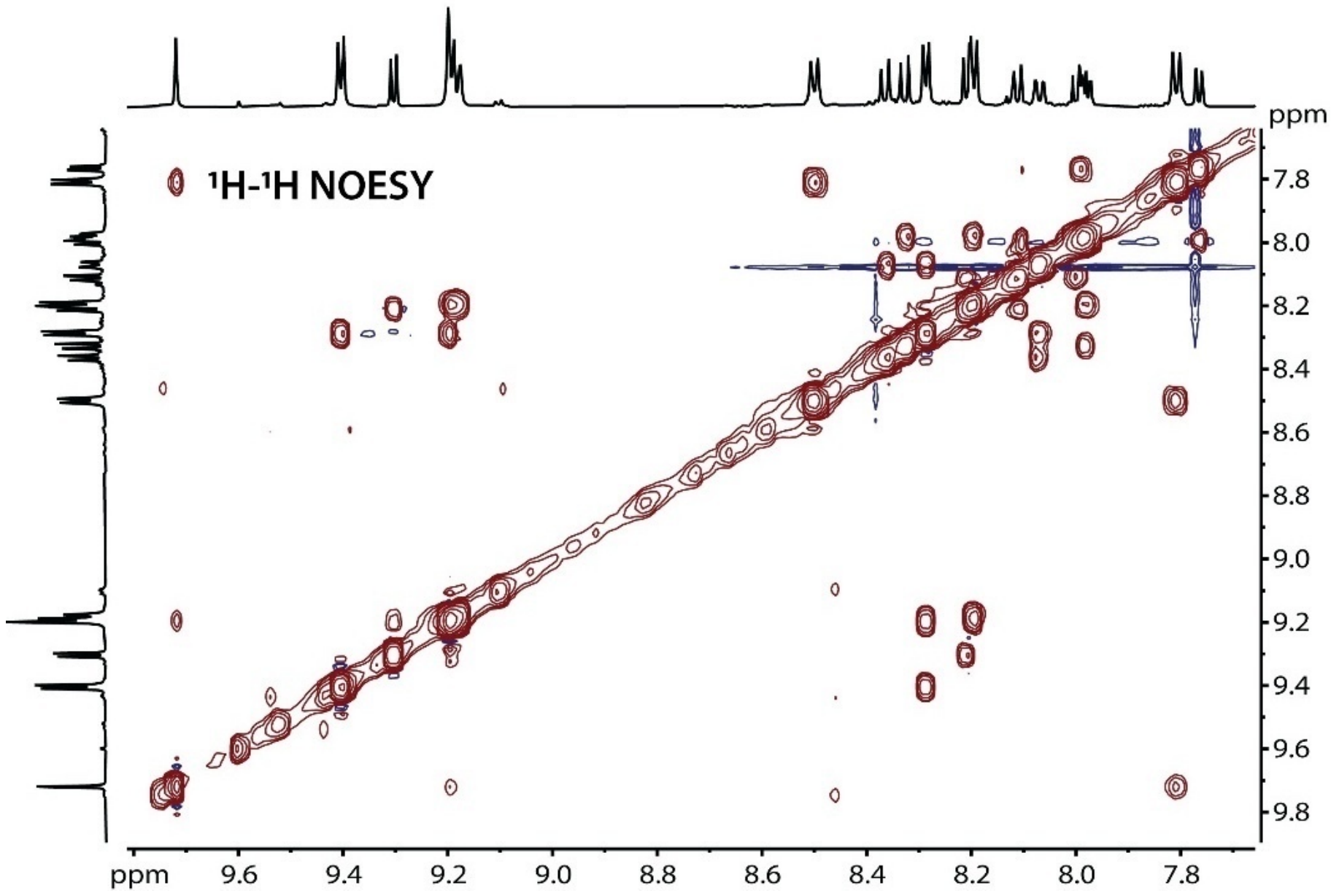

Figure S29. Partial ¹H-¹H NOESY spectrum (600 MHz, 298K, CD_3CN) of $Pd_3\mathbf{L^A}_2\mathbf{L^D}_4$.

S3.5 Cage-to-cage transformations

The cage-to-cage transformations at RT and 70 °C were studied by ¹H NMR in CD_3CN. 1,3,5-Trimethoxybenzene (1 eq.) was used as an internal standard to determine conversion.

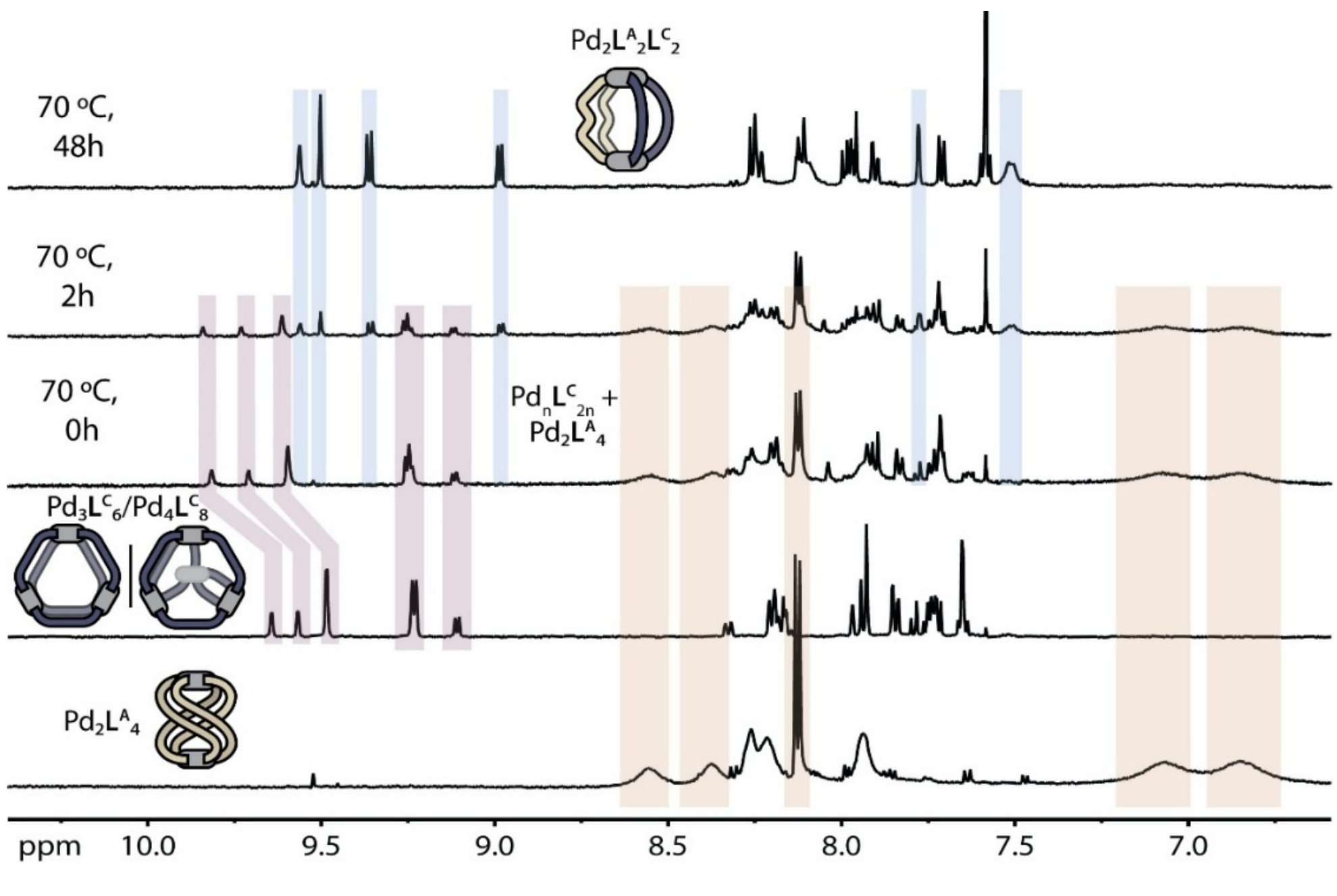

Figure S30. Stack of ¹H NMR spectra (500 MHz, 298K, CD_3CN) of $Pd_2\mathbf{L^A}_4$ and $Pd_n\mathbf{L^C}_{2n}$ (bottom two), and monitoring of their cage-to-cage transformation to $Pd_2\mathbf{L^A}_2\mathbf{L^C}_2$ after 0, 2, 48 hours (top three).

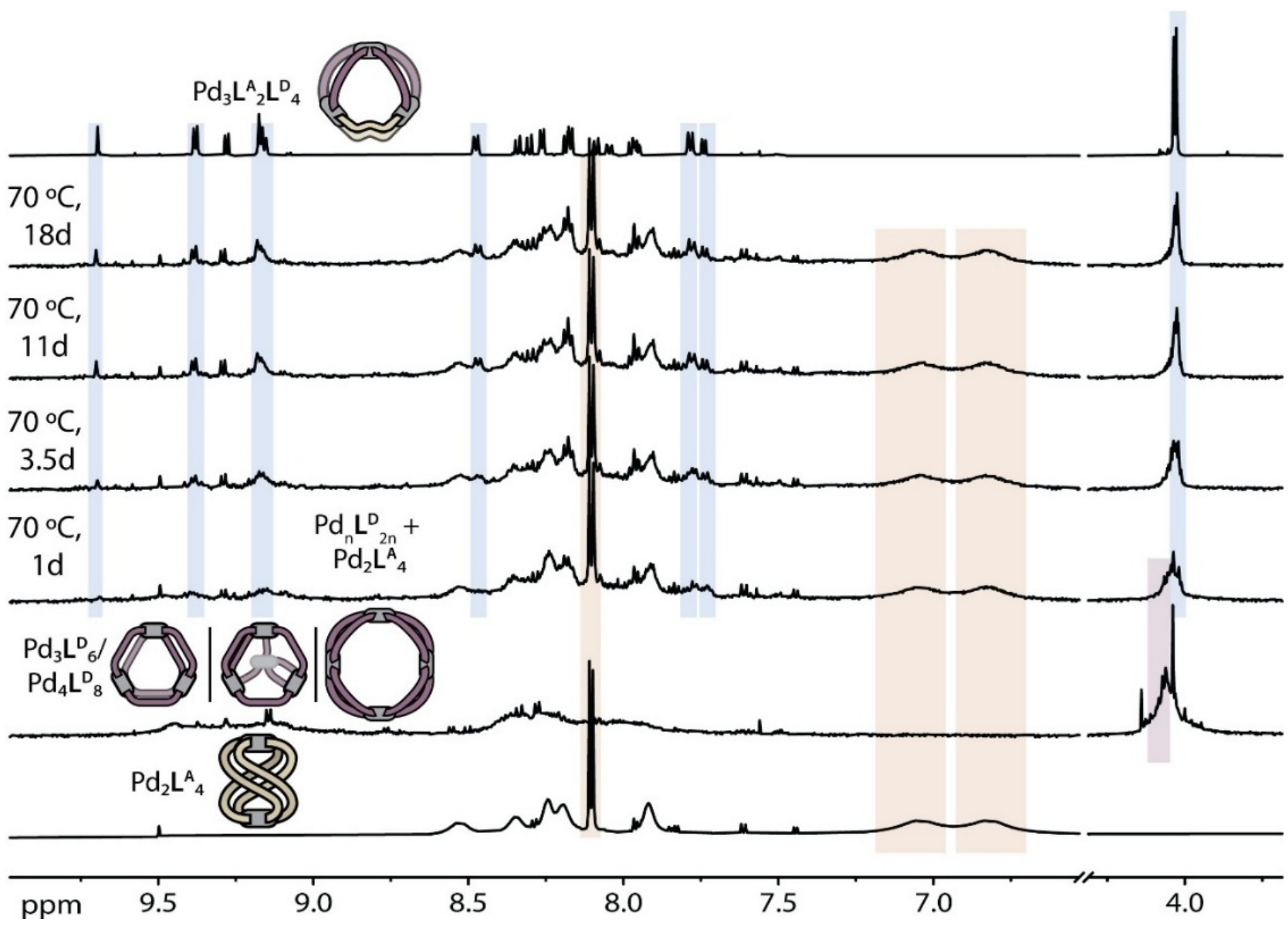

Figure S31. Stack of ^{1}H NMR spectra (500 MHz, 298K, CD_3CN) of $Pd_2\mathbf{L}^{\mathbf{A}}_4$ and $Pd_n\mathbf{L}^{\mathbf{D}}_{2n}$ (bottom two), monitoring of their cage-to-cage transformation to $Pd_3\mathbf{L}^{\mathbf{A}}_2\mathbf{L}^{\mathbf{D}}_4$ after 1, 3.5, 11, 18 days (middle four), and separately obtained $Pd_3\mathbf{L}^{\mathbf{A}}_2\mathbf{L}^{\mathbf{D}}_4$ (top).

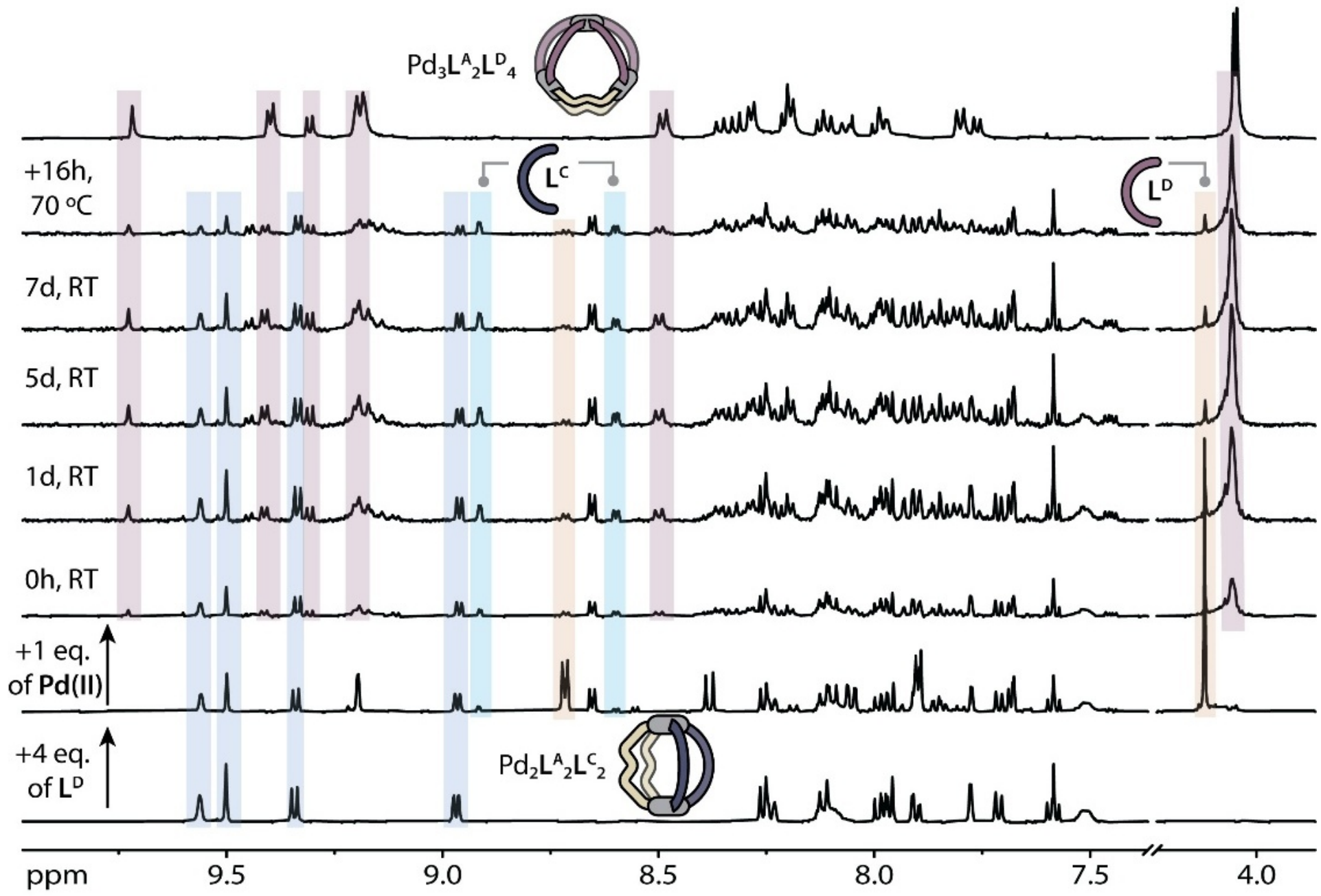

Figure S32. Stack of ^{1}H NMR spectra (500 MHz, 298K, CD_3CN) of pure $Pd_2\mathbf{L}^{\mathbf{A}}_2\mathbf{L}^{\mathbf{C}}_2$ and after addition of 4 eq. of $\mathbf{L}^{\mathbf{D}}$ and 1 eq. of Pd(II) (bottom three), monitoring of cage-to-cage transformation to $Pd_3\mathbf{L}^{\mathbf{A}}_2\mathbf{L}^{\mathbf{D}}_4$ after 1, 5, and 7 days at RT and after additional heating at 70 °C (middle five), and separately obtained pure $Pd_3\mathbf{L}^{\mathbf{A}}_2\mathbf{L}^{\mathbf{D}}_4$ (top).

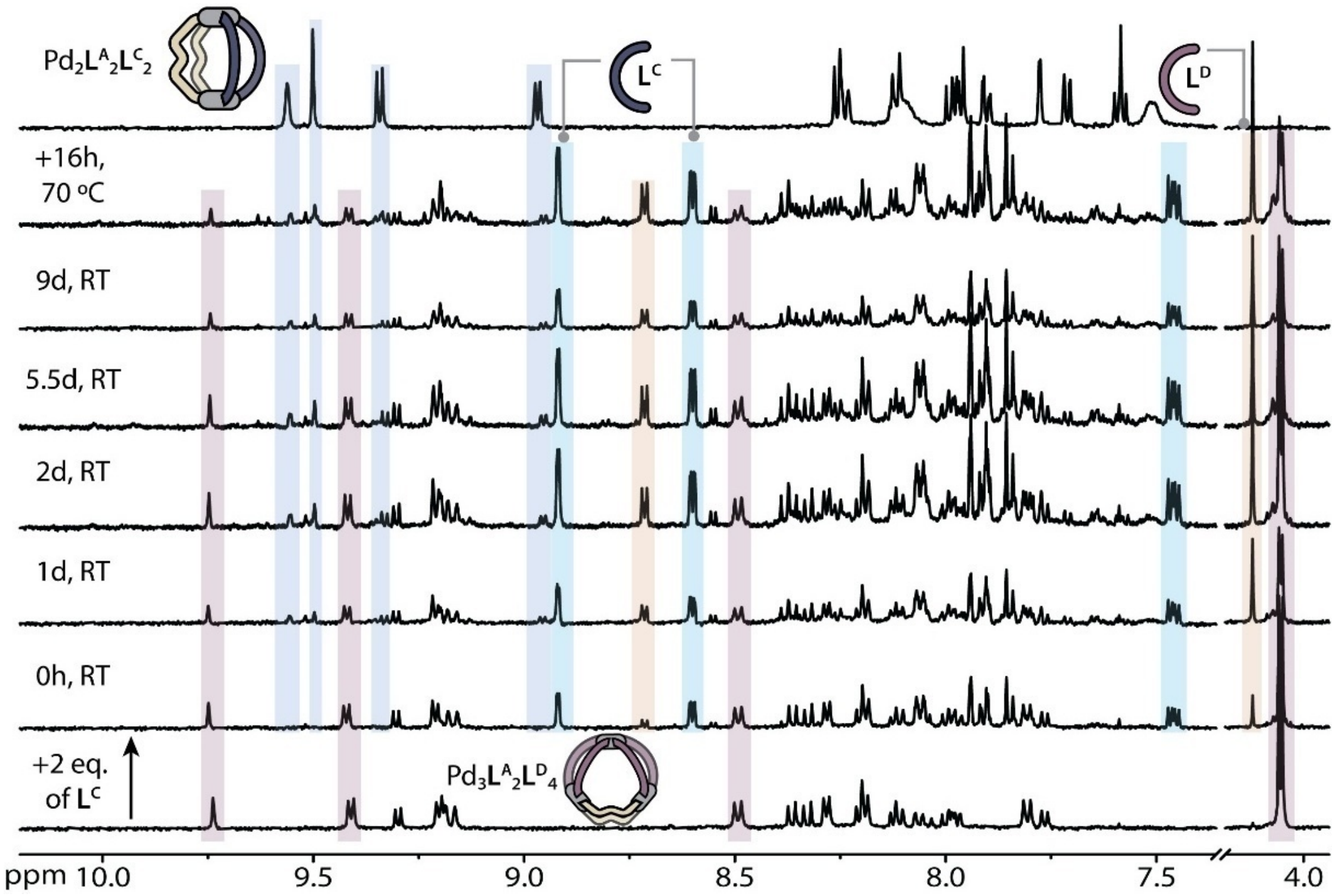

Figure S33. Stack of ^{1}H NMR spectra (500 MHz, 298K, CD_3CN) of pure $Pd_2\mathbf{L^A}_2\mathbf{L^C}_2$ and after addition of 4 eq. of $\mathbf{L^D}$ and 1 eq. of Pd(II) (bottom three), monitoring of cage-to-cage transformation to $Pd_3\mathbf{L^A}_2\mathbf{L^D}_4$ after 1, 5, and 7 days at RT and after additional heating at 70 °C (middle five), and separately obtained pure $Pd_3\mathbf{L^A}_2\mathbf{L^D}_4$ (top).

S4. Host-guest study

S4.1 Host-guest study of homoleptic and heteroleptic cages.

The host-guest chemistry of homoleptic and heteroleptic cages was studied by ^1H NMR in CD_3CN at RT. The guest displacement experiments were conducted at 70 °C for 16h in CD_3CN. 1,3,5-Trimethoxybenzene (1 eq.) was used as an internal standard to determine conversion.

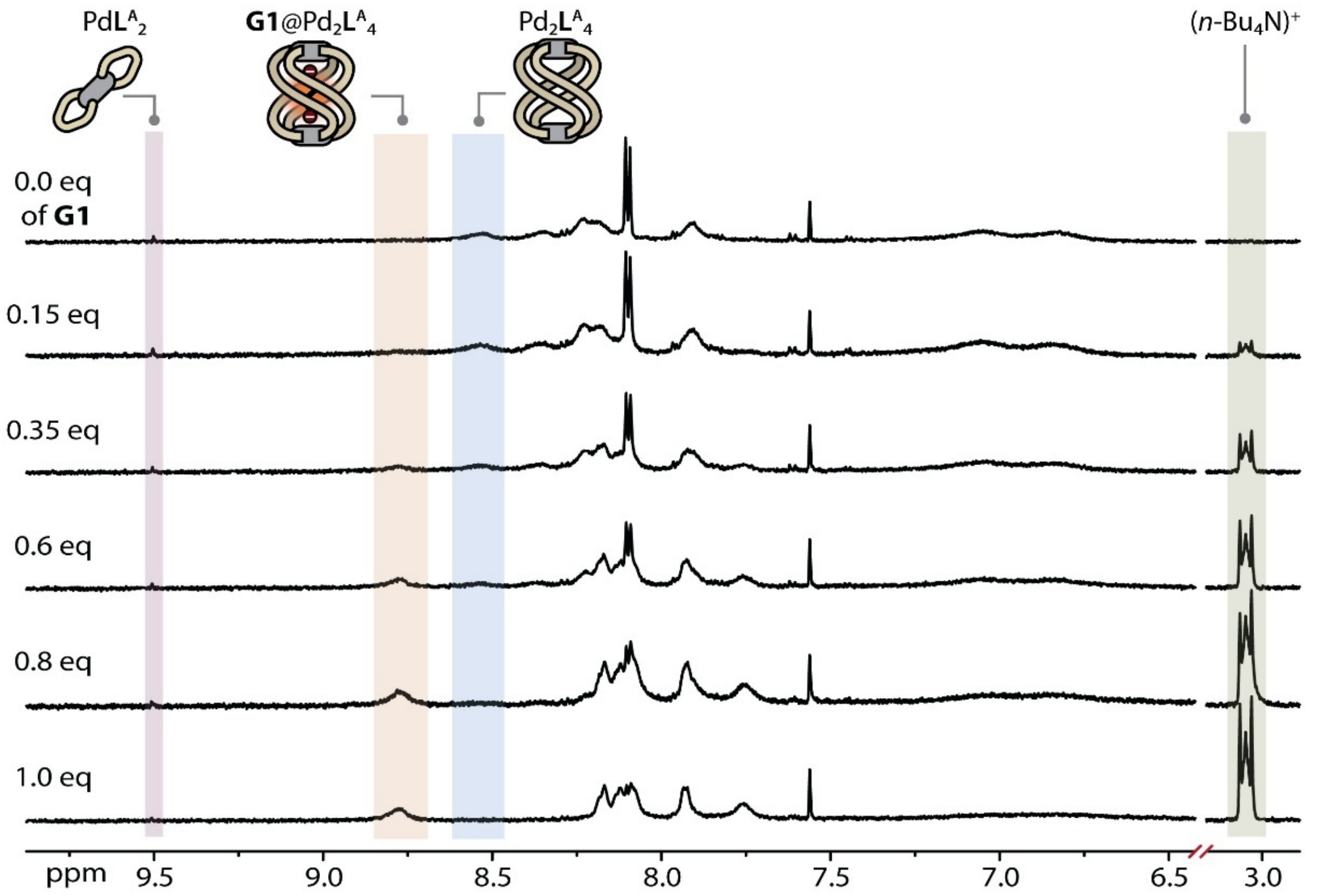

Figure S34. ^1H NMR (500 MHz, 298K, CD_3CN) titration of $[Pd_n\mathbf{L^A}_{2n}](BF_4)_{2n}$ (n = 1,2) (0.22 mM) by ethane-1,2-disulfonate (**G1**).

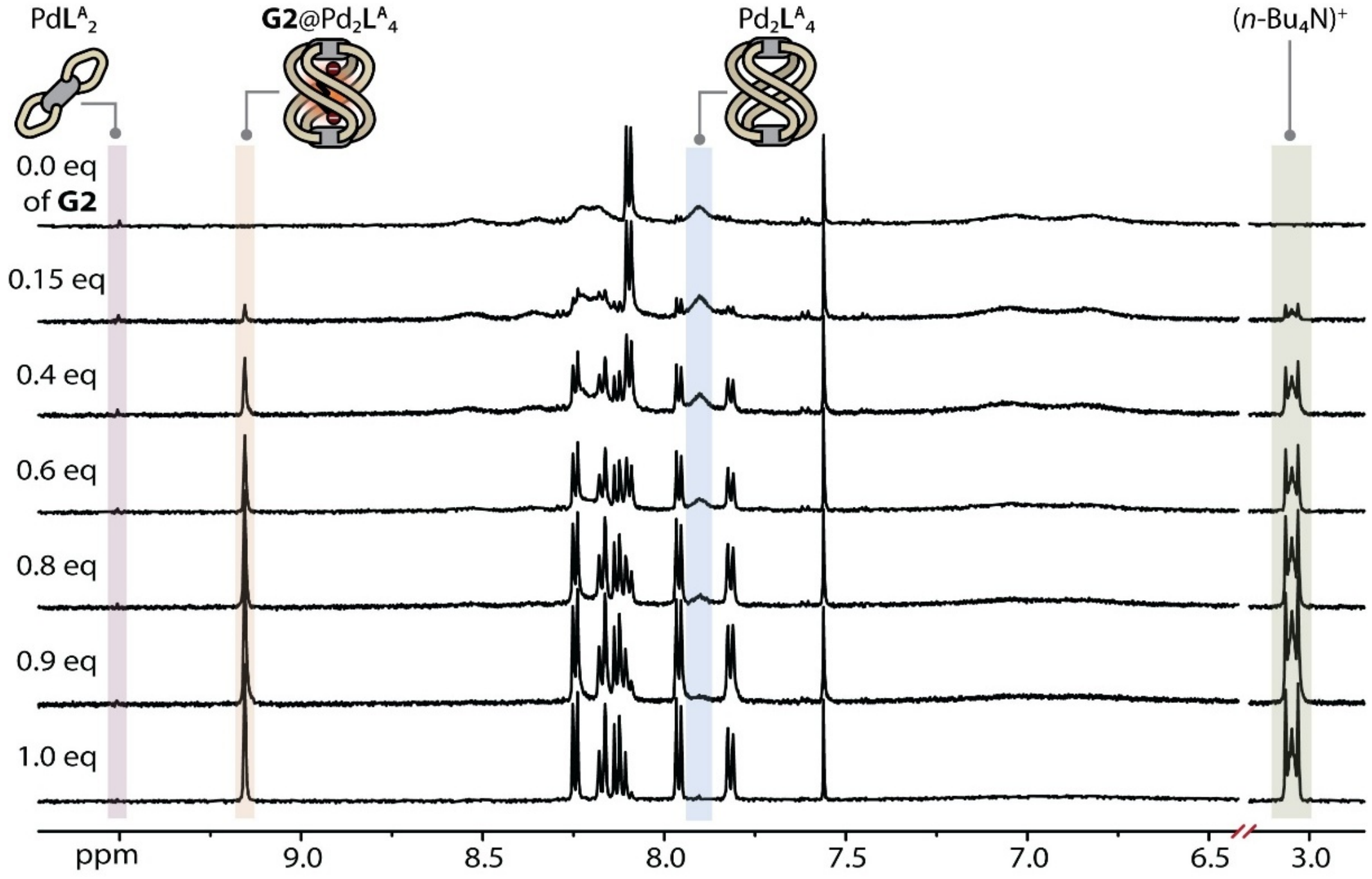

Figure S35. ^{1}H NMR (500 MHz, 298K, CD_3CN) titration of $[Pd_n\mathbf{L^A}_{2n}](BF_4)_{2n}$ (n = 1,2) (0.22 mM) by propane-1,3-disulfonate (**G2**).

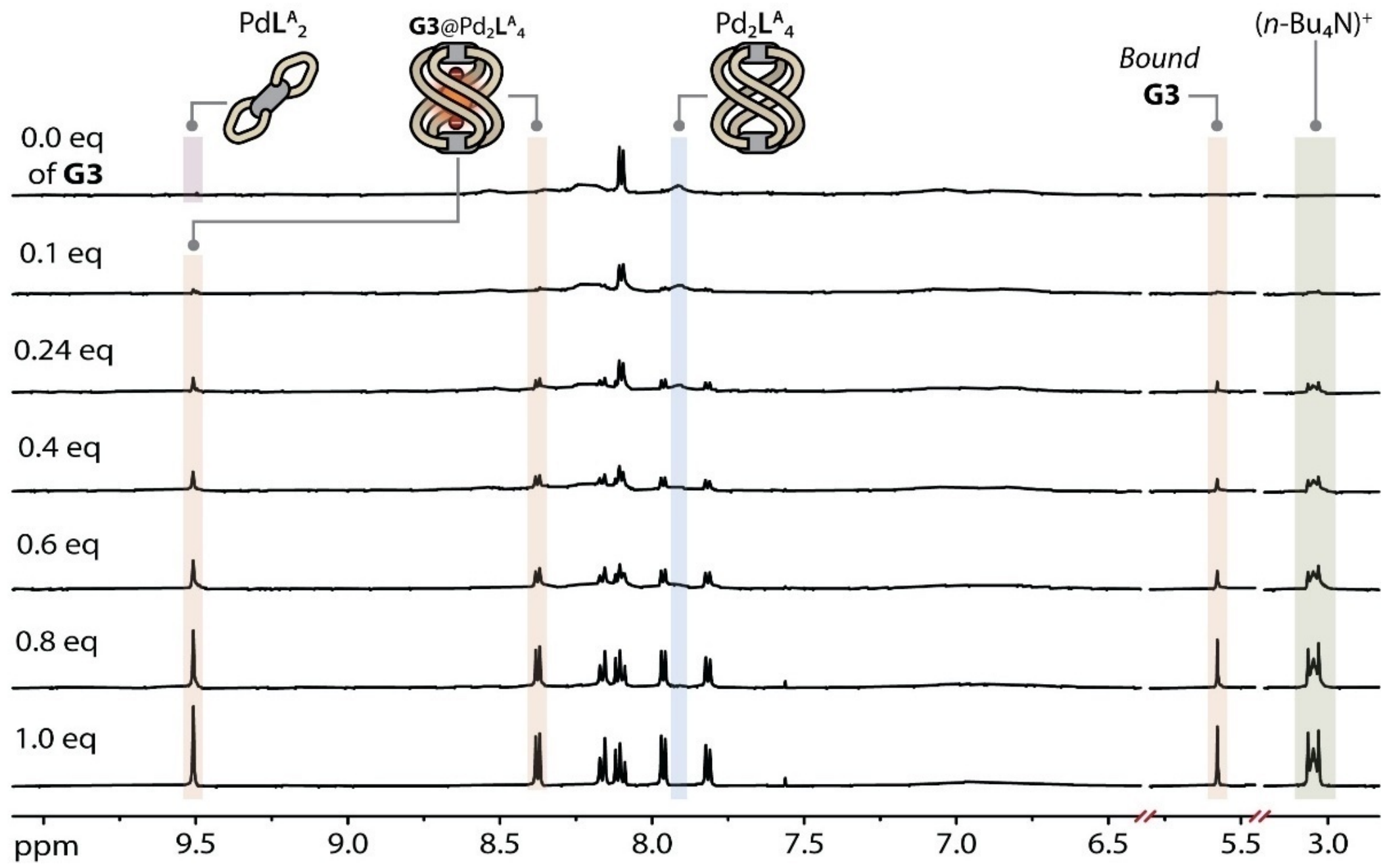

Figure S36. ^{1}H NMR (500 MHz, 298K, CD_3CN) titration of $[Pd_n\mathbf{L^A}_{2n}](BF_4)_{2n}$ (n = 1,2) (0.22 mM) by benzene-1,4-disulfonate (**G3**).

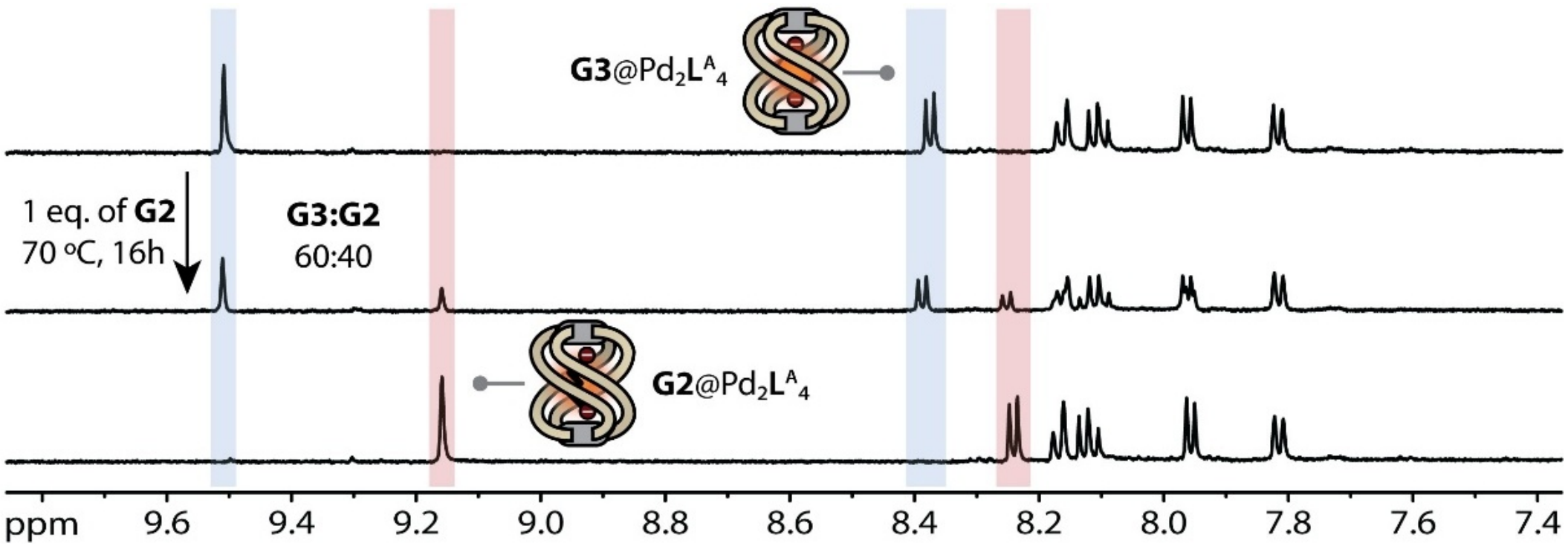

Figure S37. Stack of ^{1}H NMR (500 MHz, 298K, CD_3CN) spectra of **G3**@Pd_2**L**$^{\mathbf{A}}_4$ (top), **G3**@Pd_2**L**$^{\mathbf{A}}_4$ after addition 1 eq. of **G2** (middle), and **G2**@Pd_2**L**$^{\mathbf{A}}_4$ (bottom).

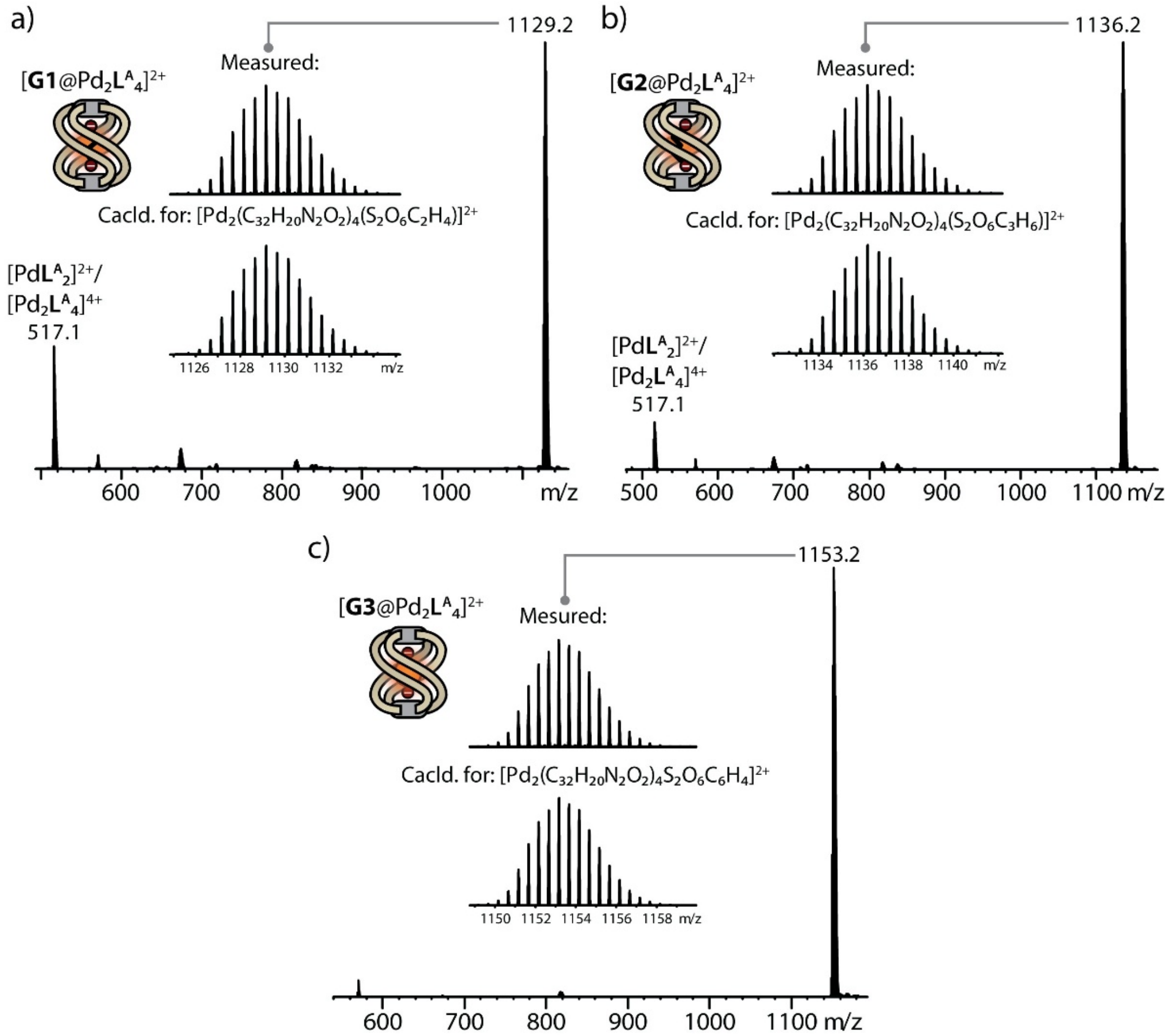

Figure S38. ESI-MS spectra of host-guest complexes (a) **G1**@Pd_2**L**$^{\mathbf{A}}_4$, (b) **G2**@Pd_2**L**$^{\mathbf{A}}_4$, (c) **G3**@Pd_2**L**$^{\mathbf{A}}_4$.

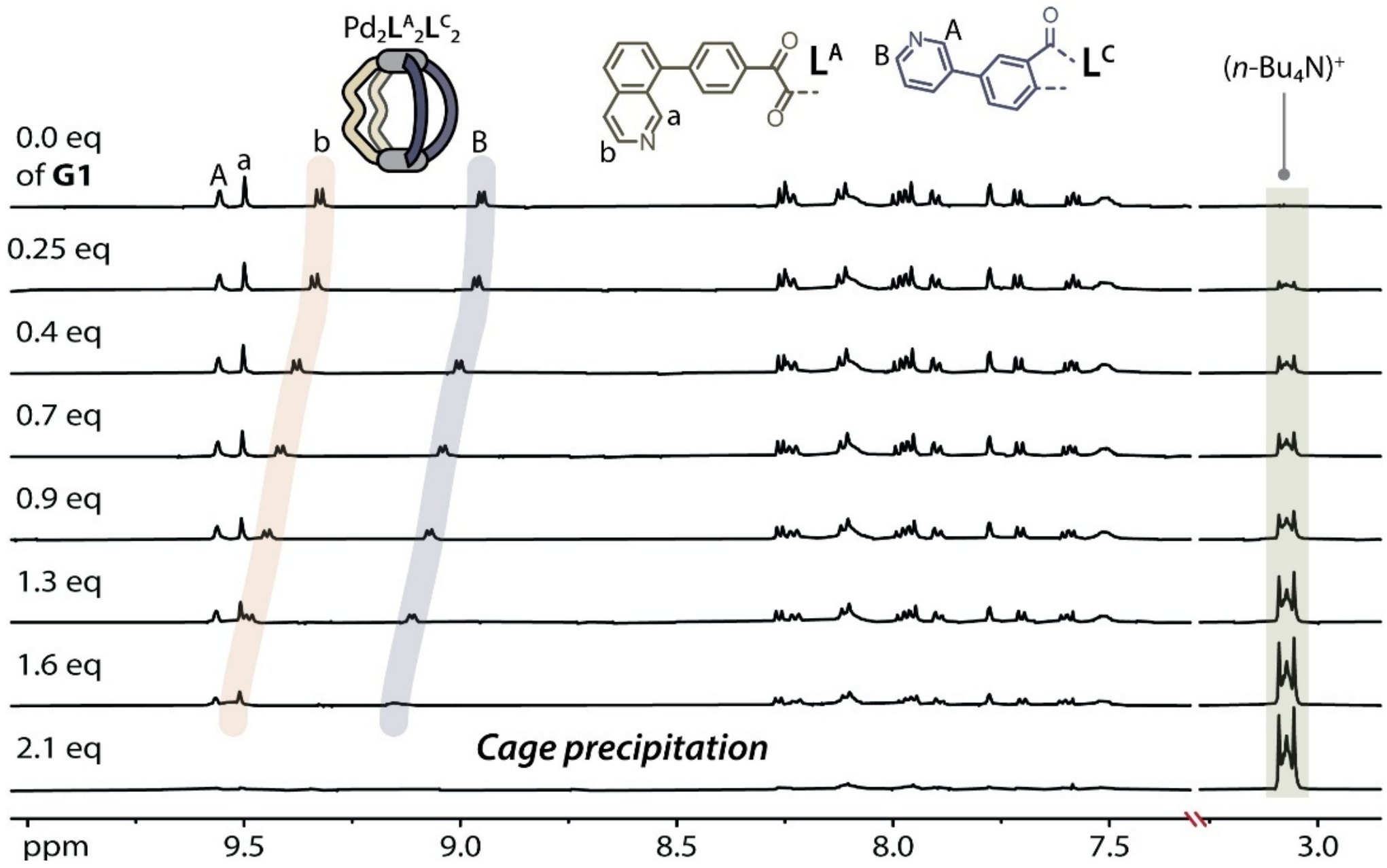

Figure S39. ^{1}H NMR (500 MHz, 298K, CD_3CN) titration of $Pd_2$$\mathbf{L^A}_2\mathbf{L^C}_2$ (0.7 mM) by ethane-1,2-disulfonate (**G1**).

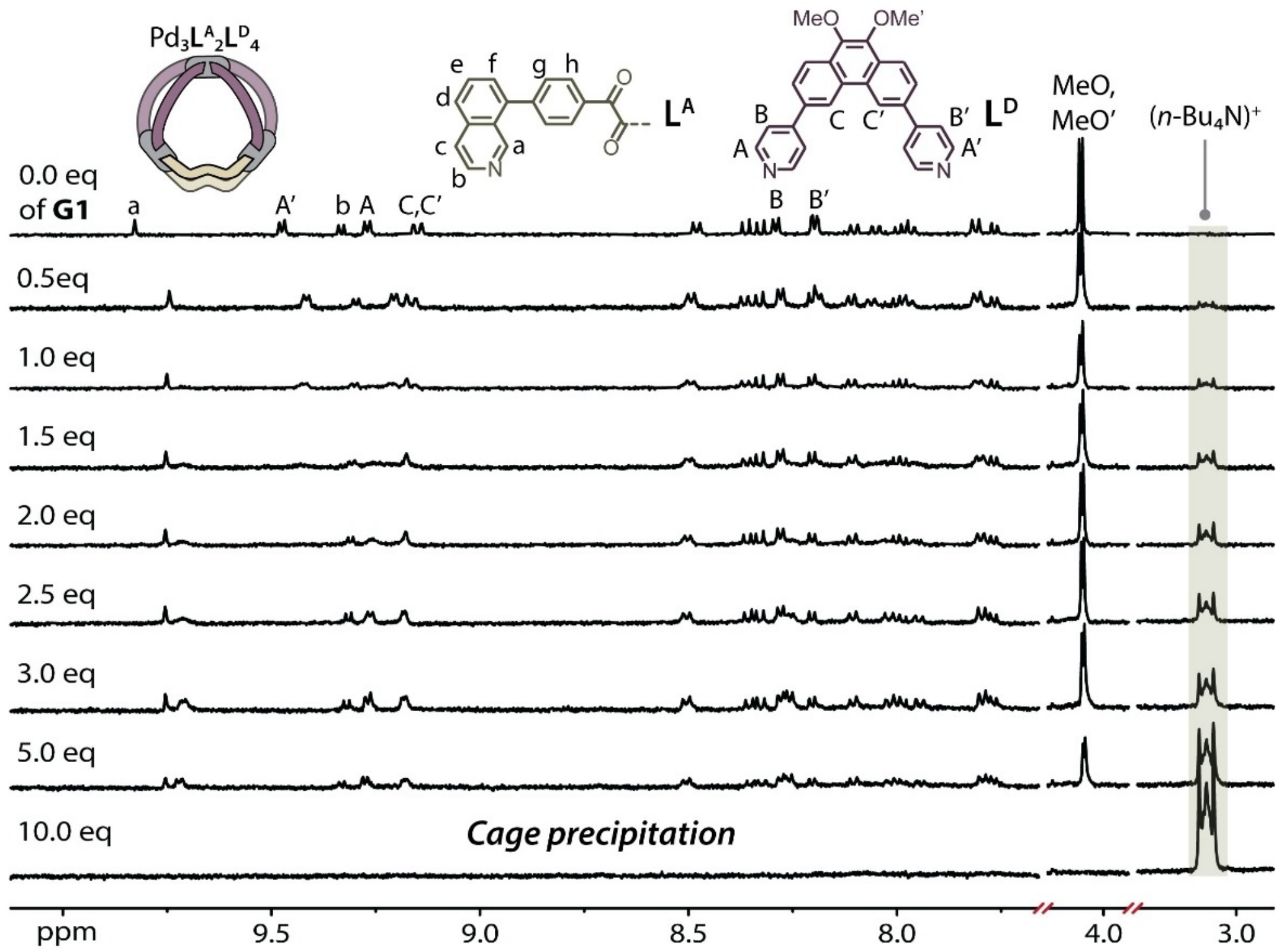

Figure S40. ^{1}H NMR (500 MHz, 298K, CD_3CN) titration of $Pd_3$$\mathbf{L^A}_2\mathbf{L^D}_4$ (0.35 mM) by ethane-1,2-disulfonate (**G1**).

S4.2 Guest-induced transformation of heteroleptic cages

The guest-induced transformations of heteroleptic cages was studied by ^{1}H NMR in CD_3CN at RT. The experiments were conducted at 70 °C in CD_3CN. 1,3,5-Trimethoxybenzene (1 eq.) was used as an internal standard. As the conversion was estimated in the relation to internal standard.

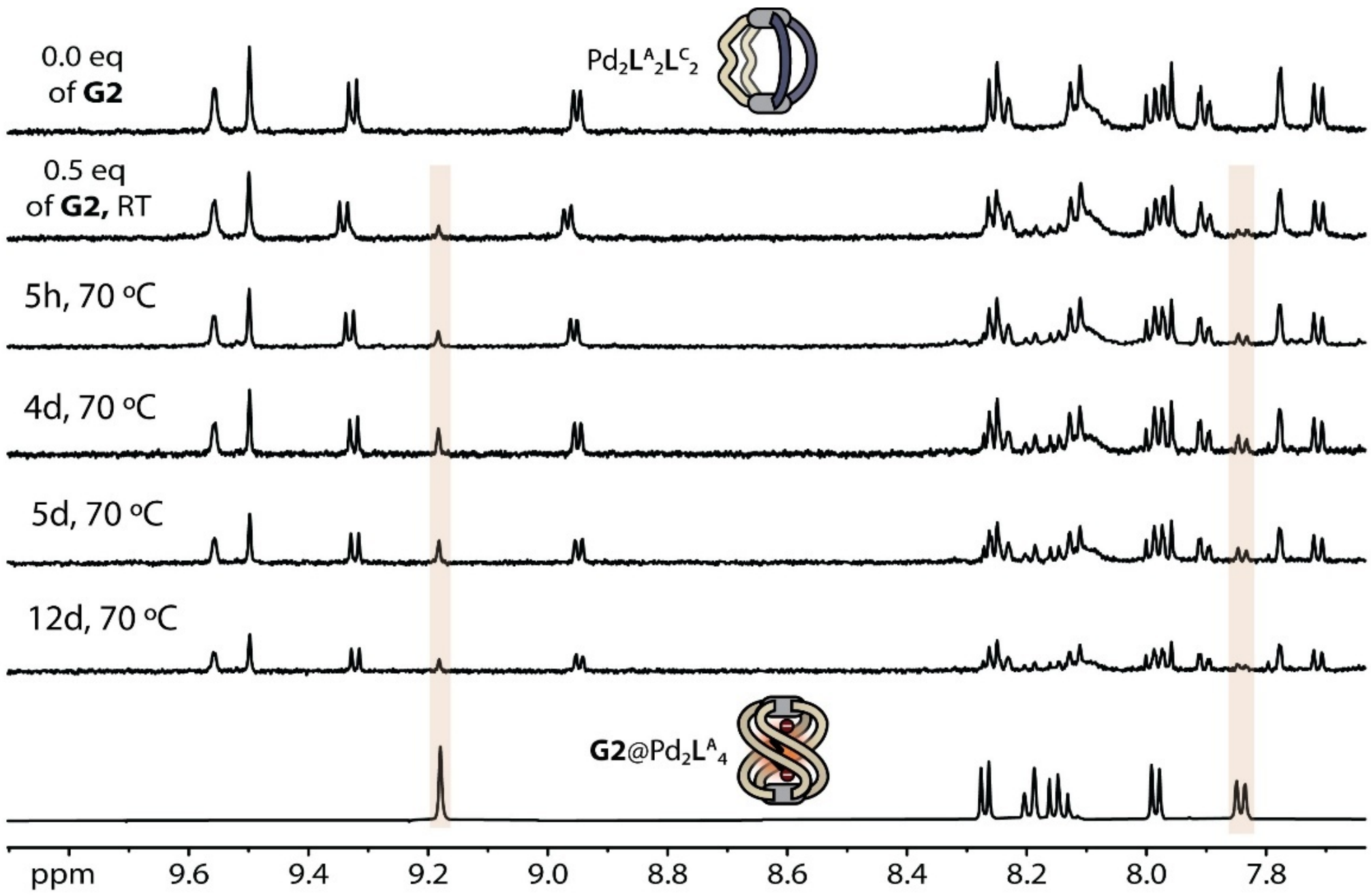

Figure S41. Stack of ^{1}H NMR (500 MHz, 298K, CD_3CN) spectra of Pd_2**L**$^A{}_2$**L**$^C{}_2$ (0.35 mM), addition of 0.5 eq. of propane-1,3-disulfonate (**G2**) to it, monitoring of the reaction at 70 °C at 5h, 4d, 5d, 12d, and separately obtained pure **G2**@Pd_2**L**$^A{}_4$.

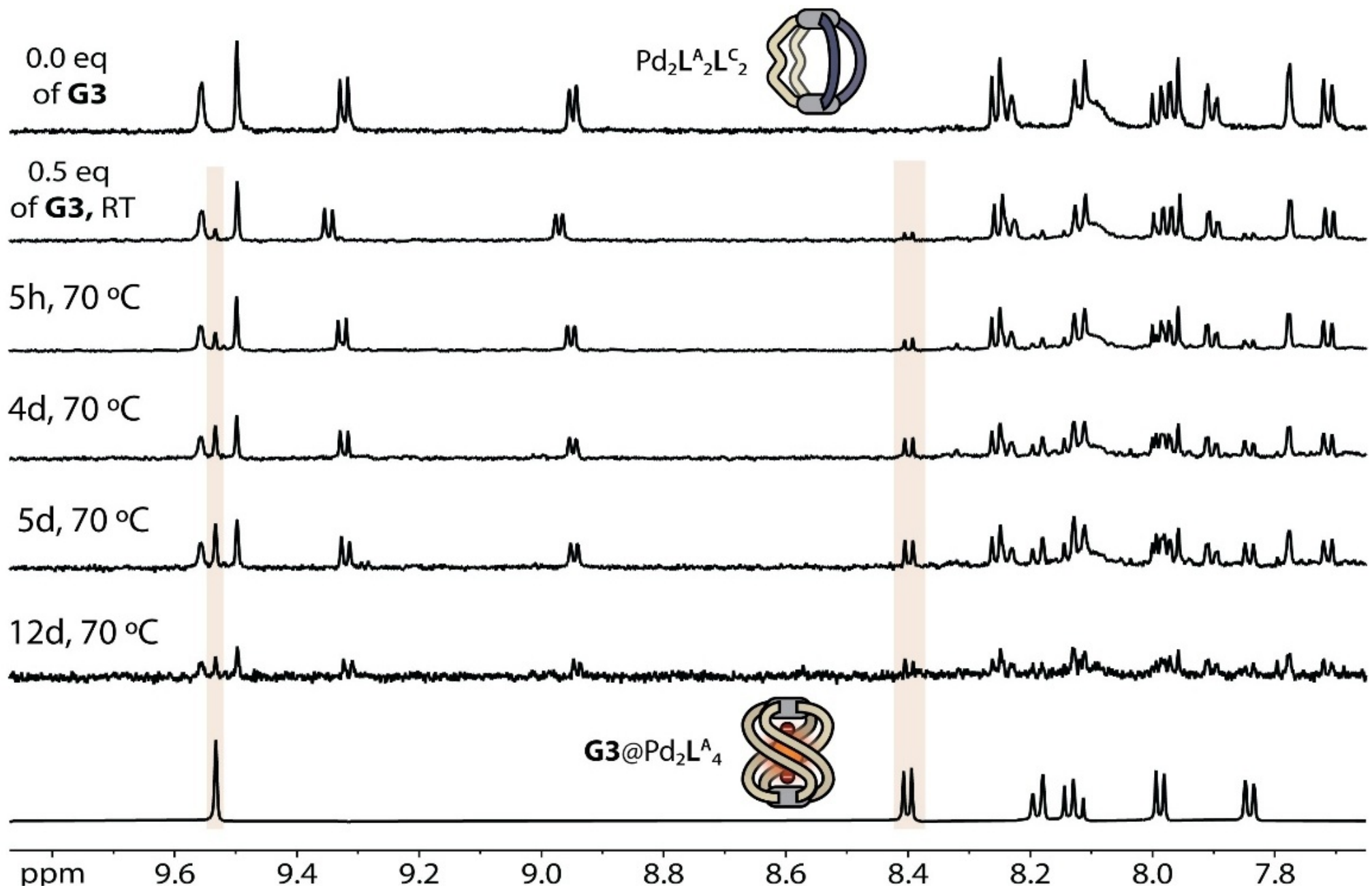

Figure S42. Stack of ^{1}H NMR (500 MHz, 298K, CD_3CN) spectra of Pd_2**L**$^A{}_2$**L**$^C{}_2$ (0.35 mM), addition of 0.5 eq. of benzne-1,4-disulfonate (**G3**) to it, monitoring of the reaction at 70 °C at 5h, 4d, 5d, 12d, and separately obtained pure **G3**@Pd_2**L**$^A{}_4$.

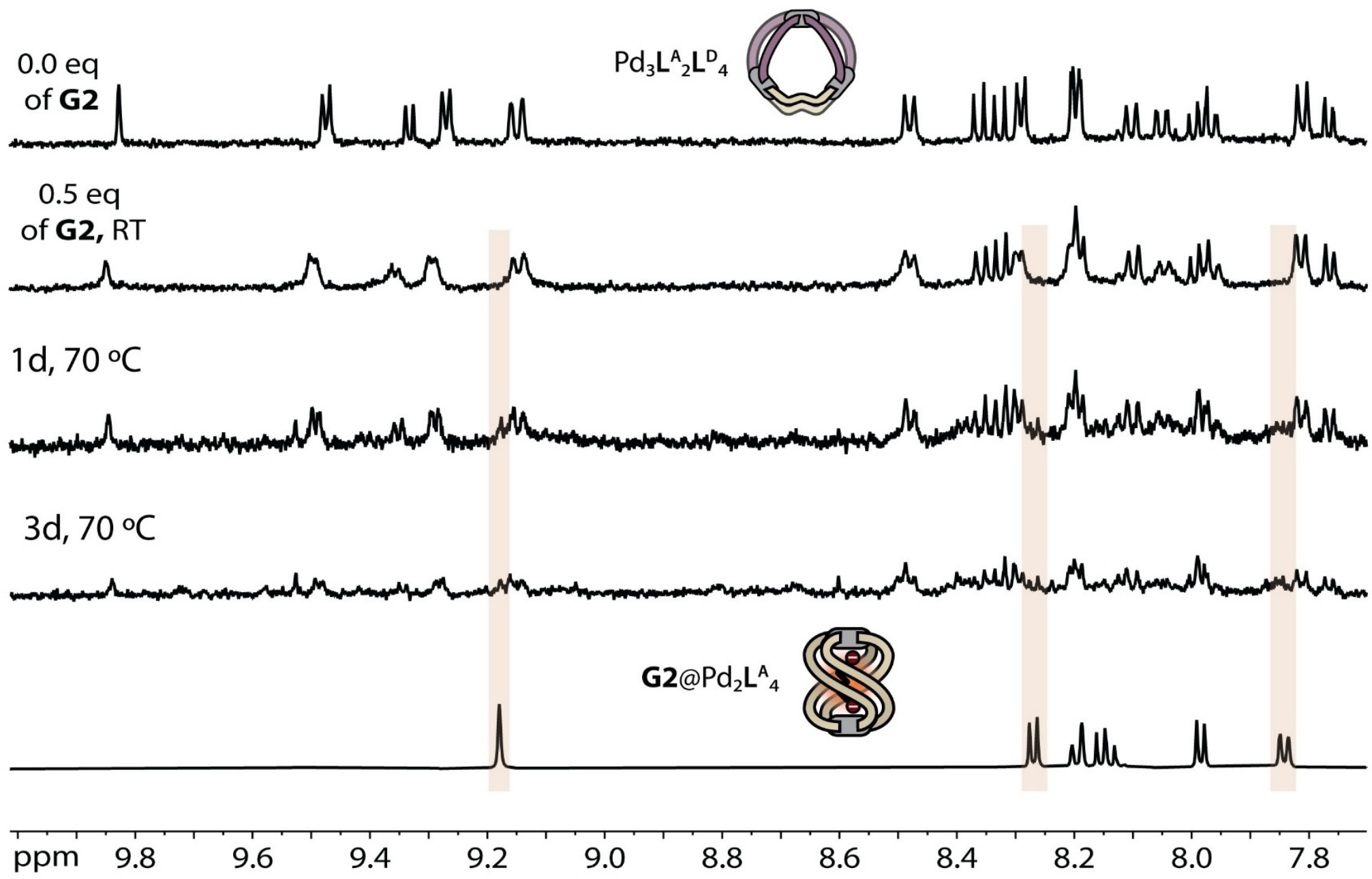

Figure S43. Stack of ^{1}H NMR (500 MHz, 298K, CD_3CN) spectra of $Pd_3\mathbf{L}^{\mathbf{A}}{}_2\mathbf{L}^{\mathbf{D}}{}_4$ (0.35 mM), addition of 0.5 eq. of propane-1,3-disulfonate (**G2**) to it, monitoring of the reaction at 70 °C at 1d and 3d, and separately obtained pure **G2**@$Pd_2\mathbf{L}^{\mathbf{A}}{}_4$.

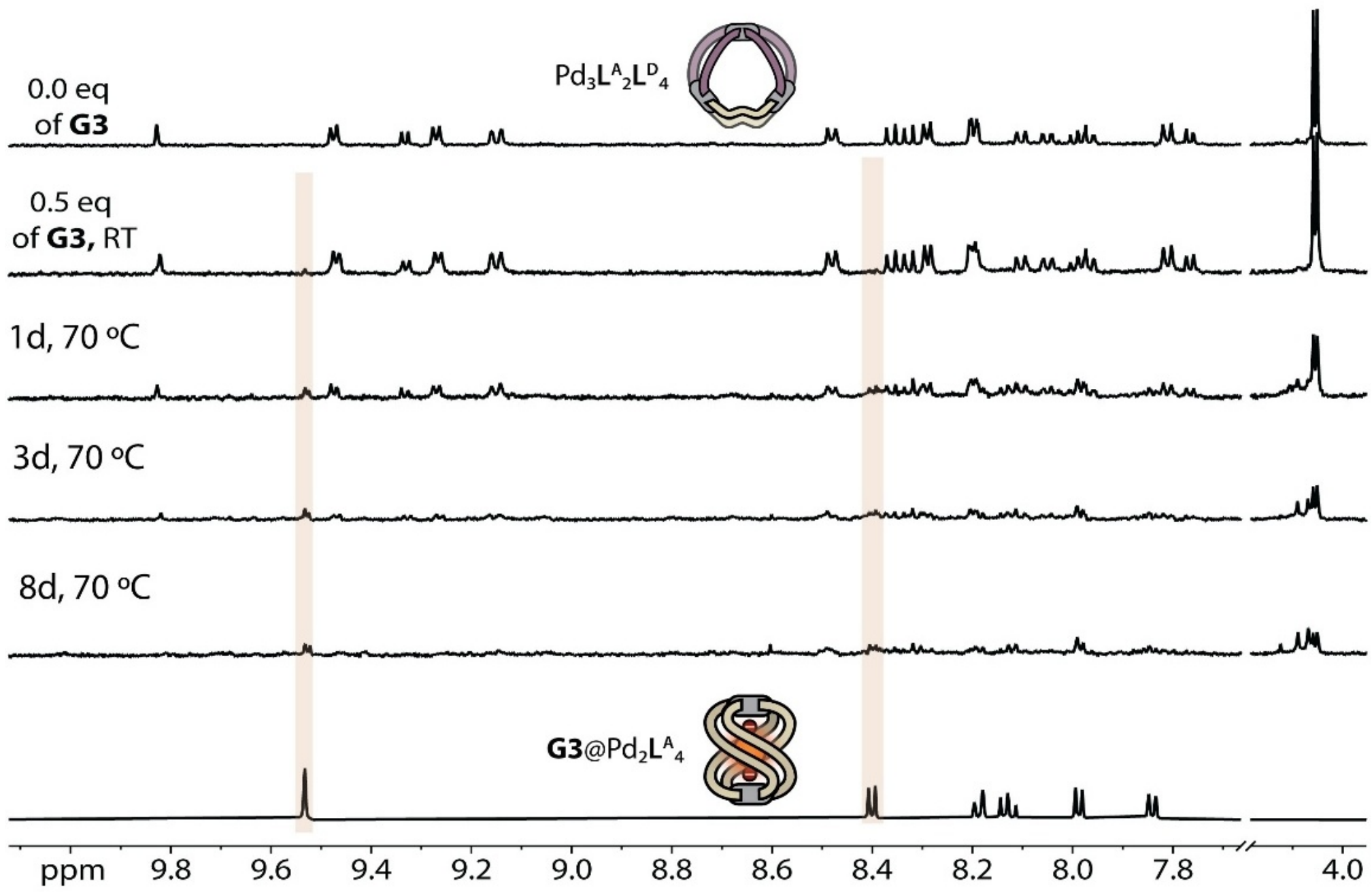

Figure S44. Stack of ^{1}H NMR (500 MHz, 298K, CD_3CN) spectra of $Pd_3\mathbf{L}^{\mathbf{A}}{}_2\mathbf{L}^{\mathbf{D}}{}_4$ (0.35 mM), addition of 0.5 eq. of benzne-1,4-disulfonate (**G3**) to it, monitoring of the reaction at 70 °C at 1d, 3d, 8d, and separately obtained pure **G3**@$Pd_2\mathbf{L}^{\mathbf{A}}{}_4$.

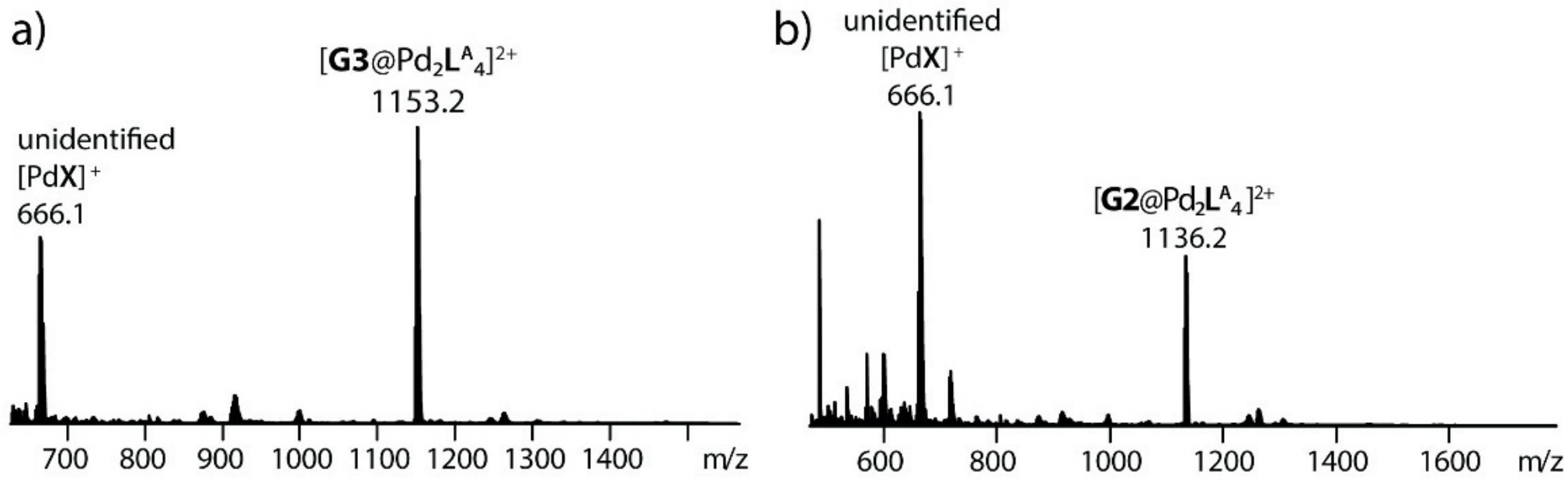

Figure S45. ESI-MS spectra of reaction mixtures of $Pd_3\mathbf{L}^{A}{}_2\mathbf{L}^{D}{}_4$ with **G3** (a) and **G2** (b).

S5. Post-assembly modification

The post-assembly condensation of homo- and heteroleptic cages with corresponding amounts of 1,2-phenylenediamine at RT and 70 °C was studied by ^1H NMR in CD_3CN. 1,3,5-Trimethoxybenzene was used as an internal standard to determine conversion.

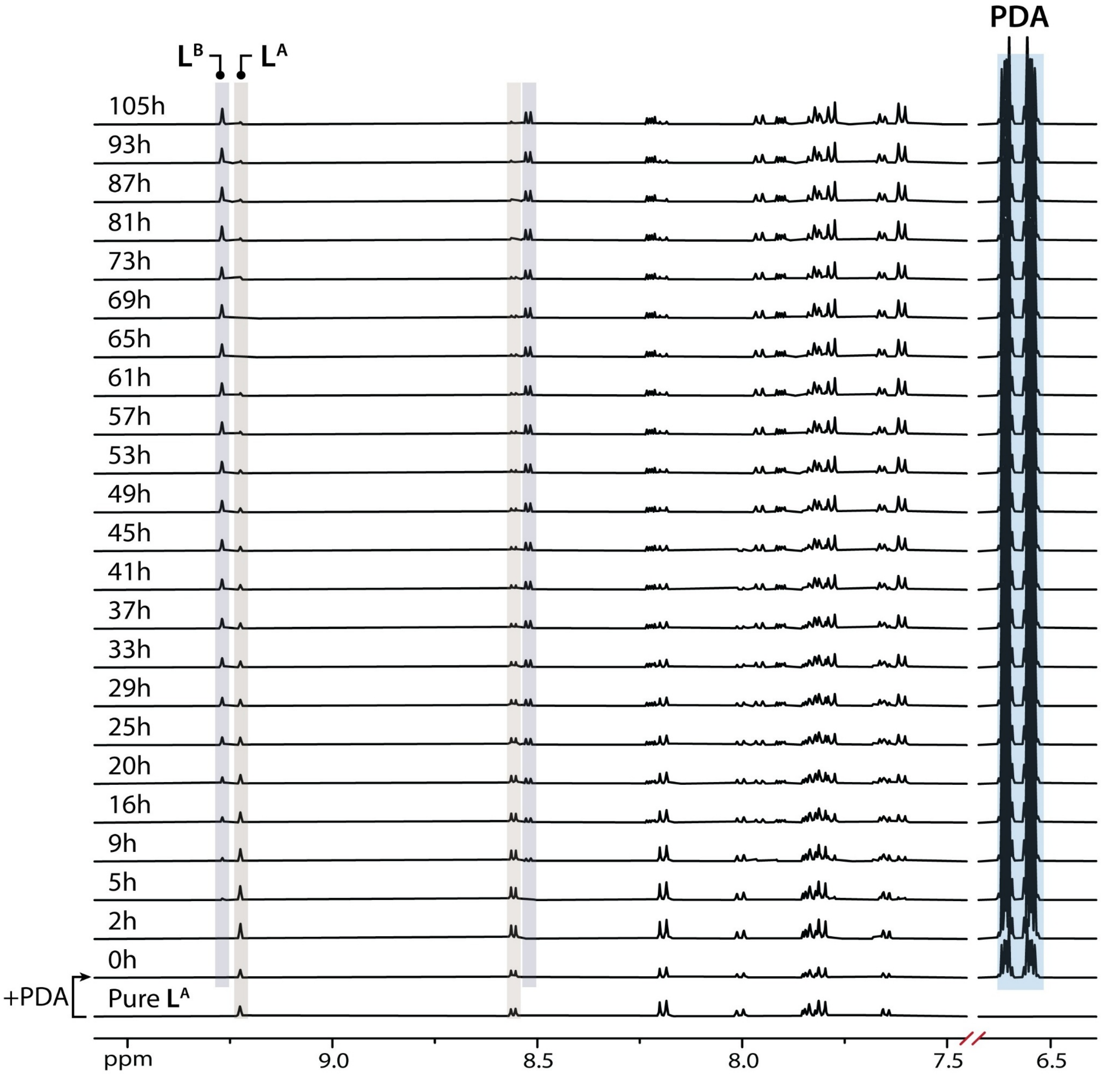

Figure S46. ^1H NMR (500 MHz, 298K, CD_3CN) monitoring of the reaction of **L^A** (3.1 mM used concertation, 0.3 mM actual concentration due to low solubility) with PDA (3.1 mM) at RT.

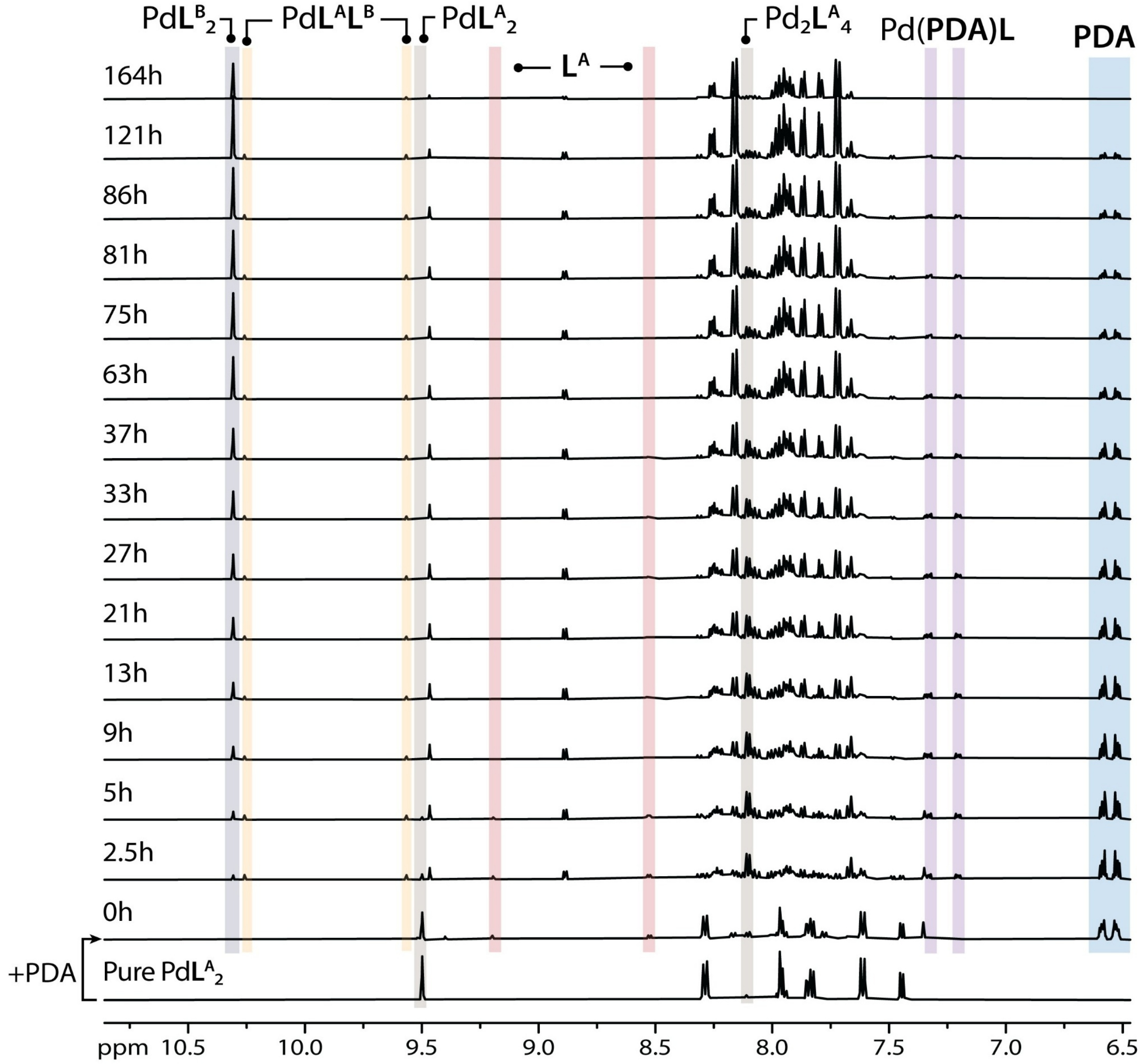

Figure S47. ^{1}H NMR (500 MHz, 298K, CD_3CN) monitoring of the reaction of freshly prepared Pd$\mathbf{L^A}_2$ (1.4 mM) with PDA (3.1 mM) at RT.

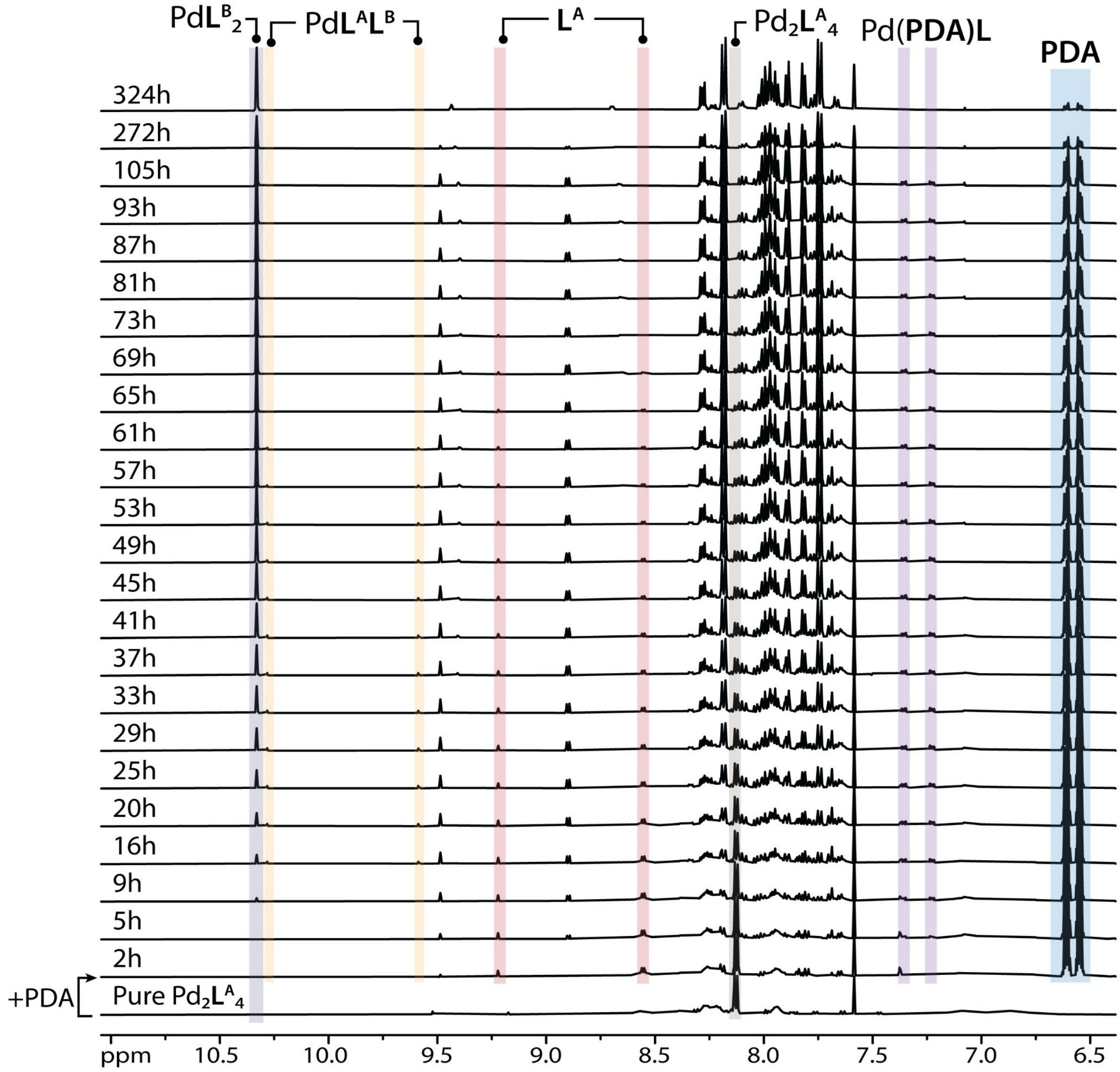

Figure S48. ^{1}H NMR (500 MHz, 298K, CD_3CN) monitoring of the reaction of $Pd_2\mathbf{L}^{\mathbf{A}}_4$ (0.7 mM) with PDA (3.1 mM) at RT.

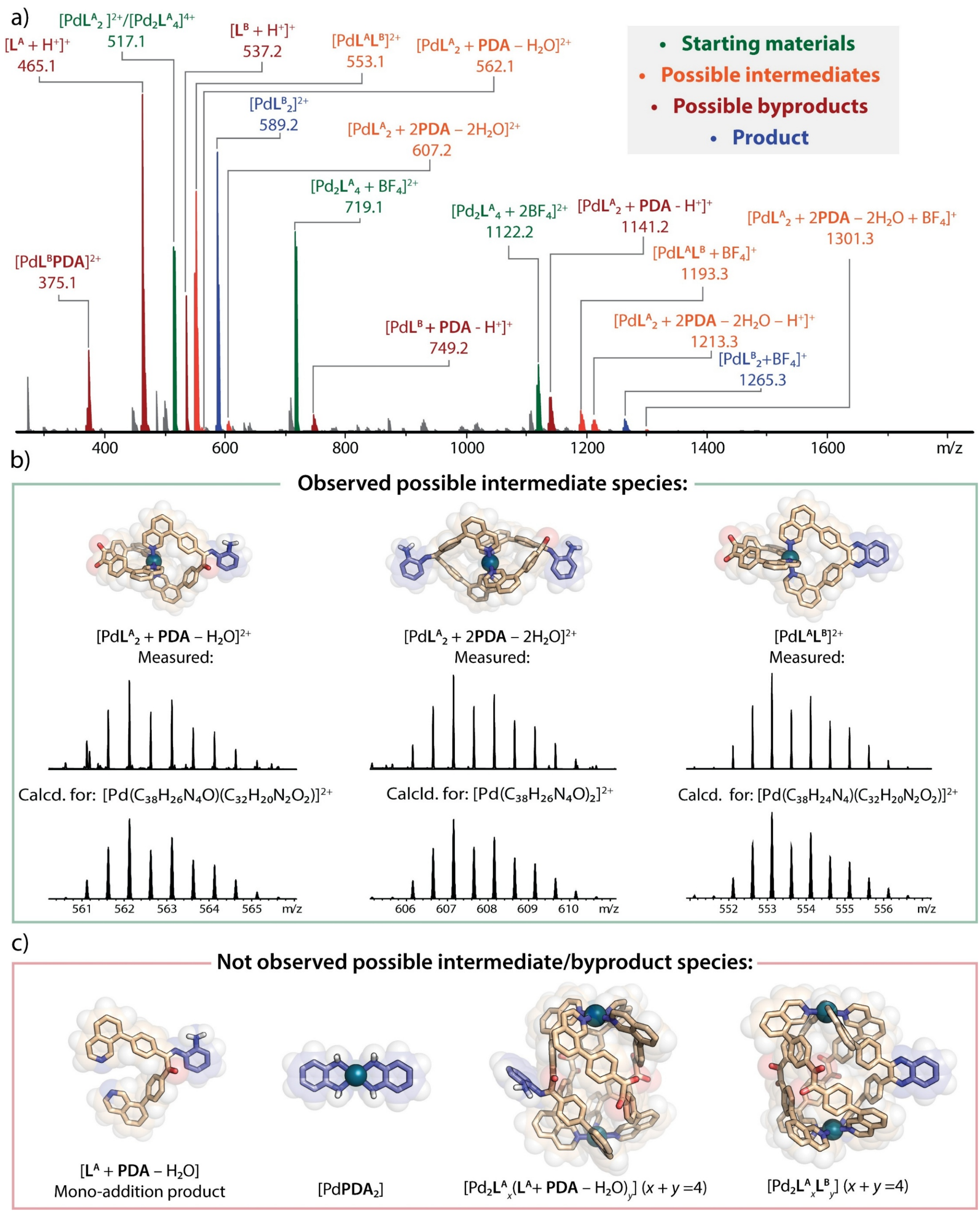

Figure S49. a) ESI-MS spectrum of the reaction mixture of $Pd_2\mathbf{L^A}_4$ with **PDA** at 70 °C; b) Selected observed signals and structures corresponding to possible reaction intermediates; c) The structures of not observed possible reaction intermediates and byproducts.

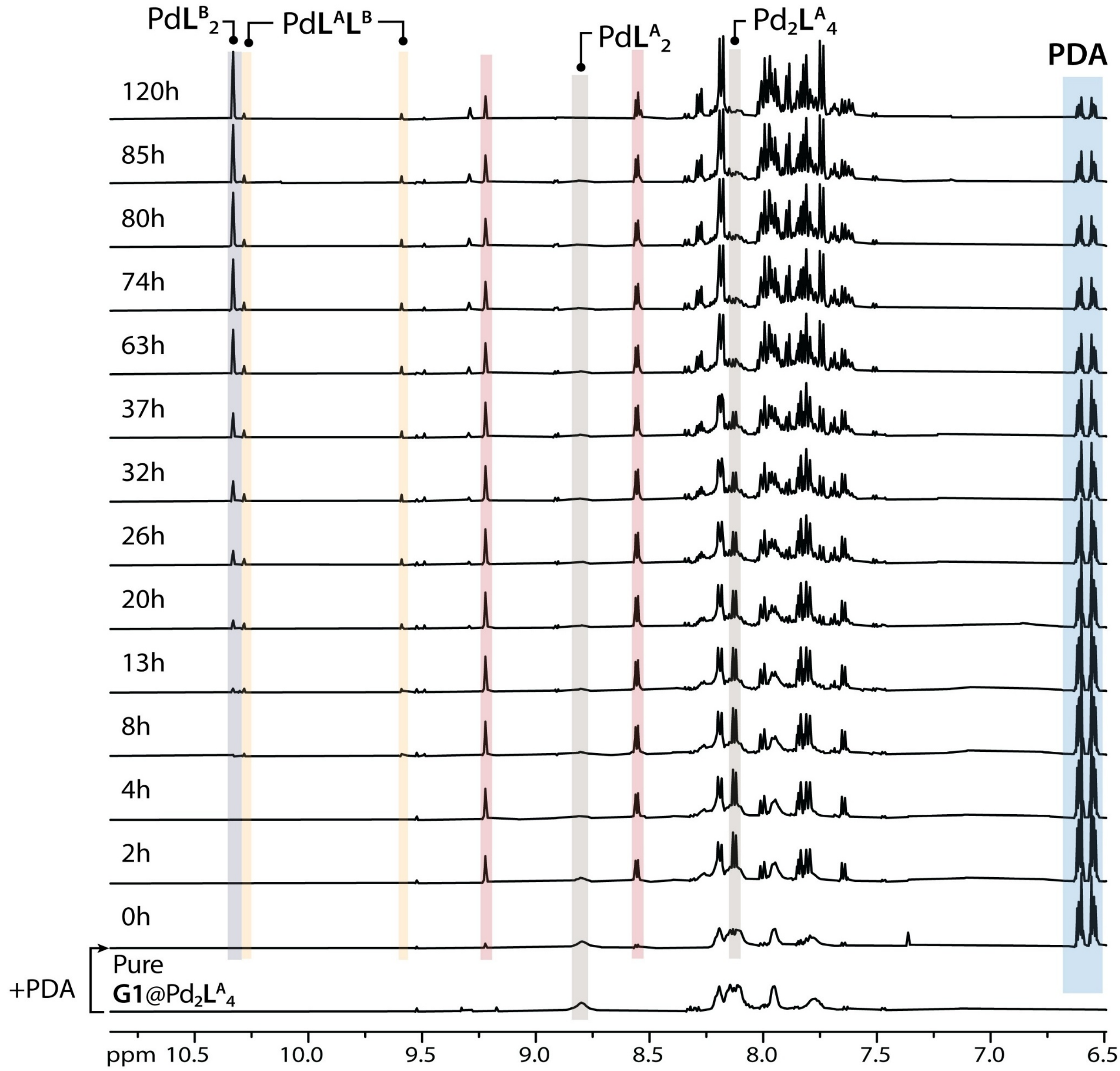

Figure S50. ^{1}H NMR (500 MHz, 298K, CD_3CN) monitoring of the reaction of **G1**@Pd_2**L**$^{A}_4$ (0.7 mM) with PDA (3.1 mM) at RT.

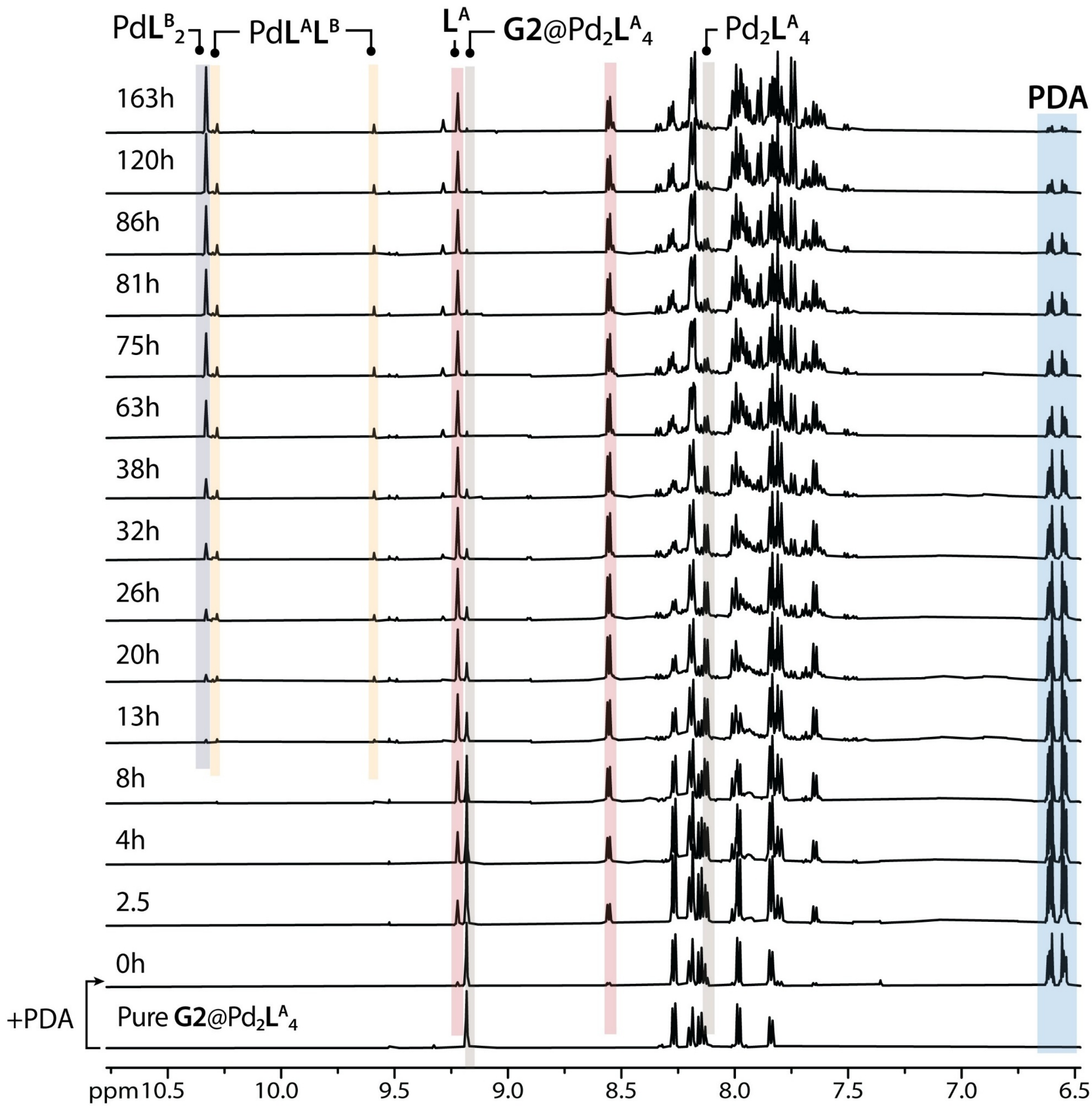

Figure S51. ^{1}H NMR (500 MHz, 298K, CD_3CN) monitoring of the reaction of **G2**@$Pd_2$$\mathbf{L}^A_4$ (0.7 mM) with PDA (3.1 mM) at RT.

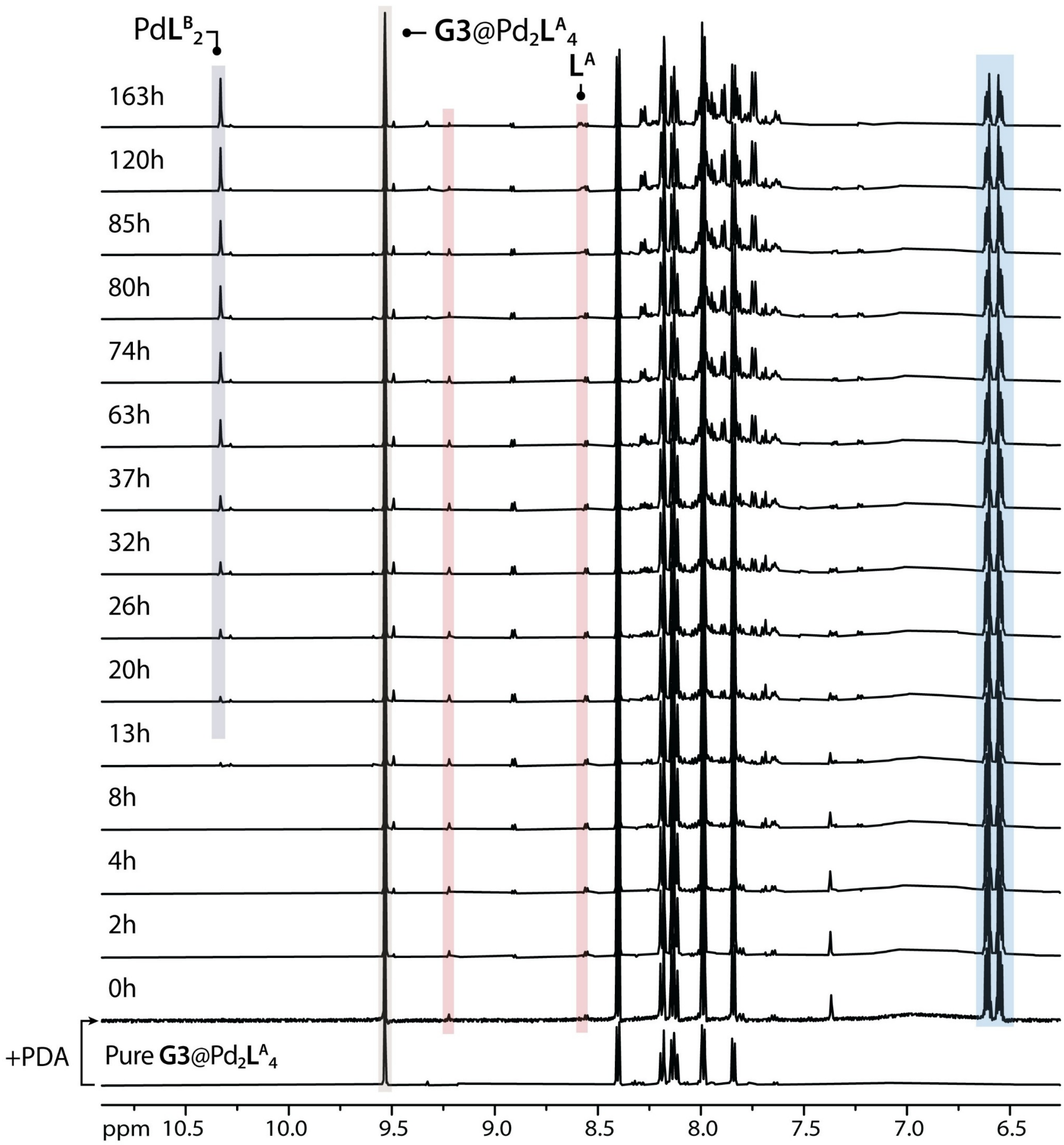

Figure S52. ^{1}H NMR (500 MHz, 298K, CD_3CN) monitoring of the reaction of **G3**@Pd_2**L**$^A_4$ (0.7 mM) with PDA (3.1 mM) at RT.

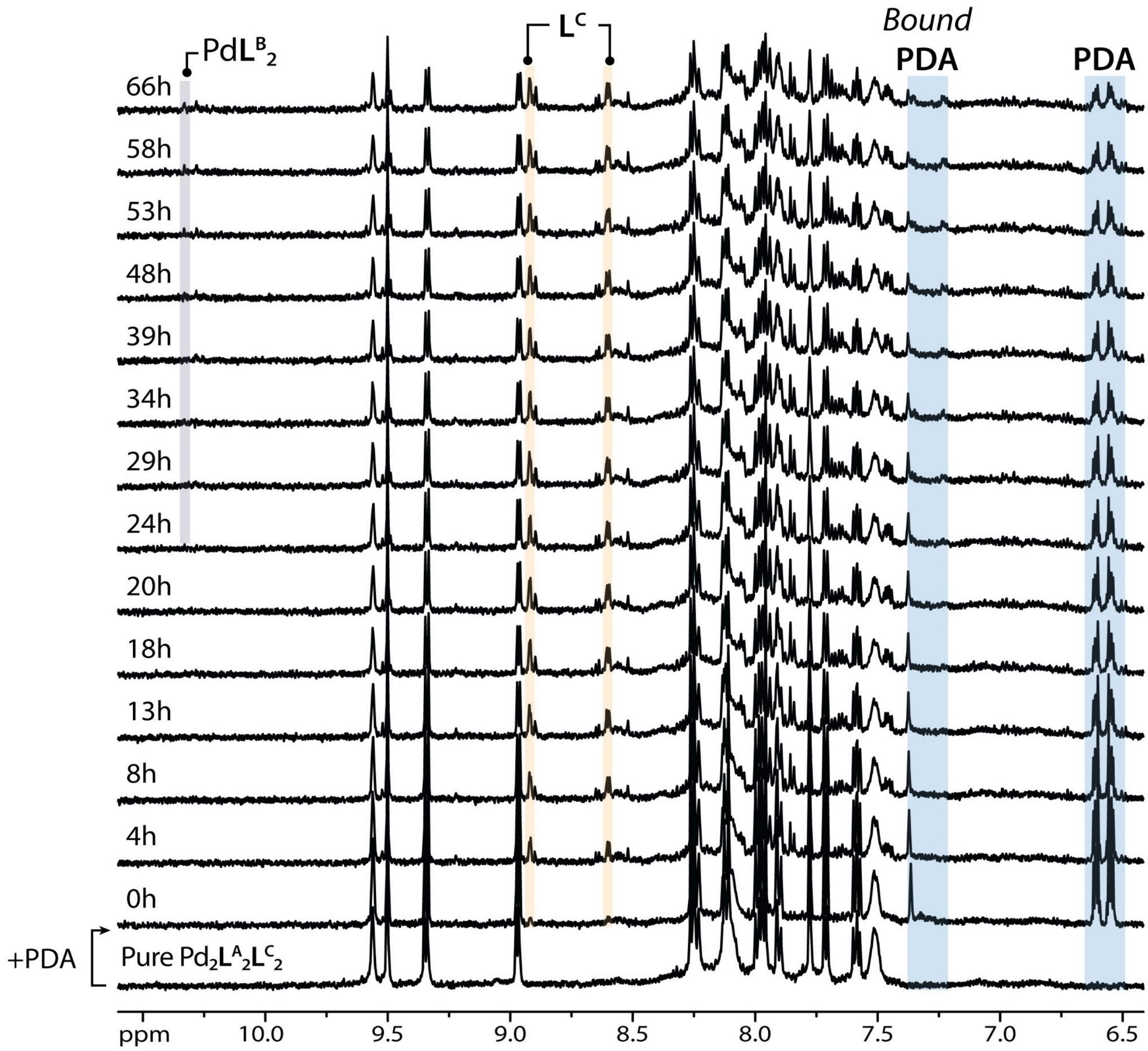

Figure S53. ^{1}H NMR (500 MHz, 298K, CD_3CN) monitoring of the reaction of $Pd_2\mathbf{L^A}_2\mathbf{L^C}_2$ (0.7 mM) with PDA (1.5 mM) at RT.

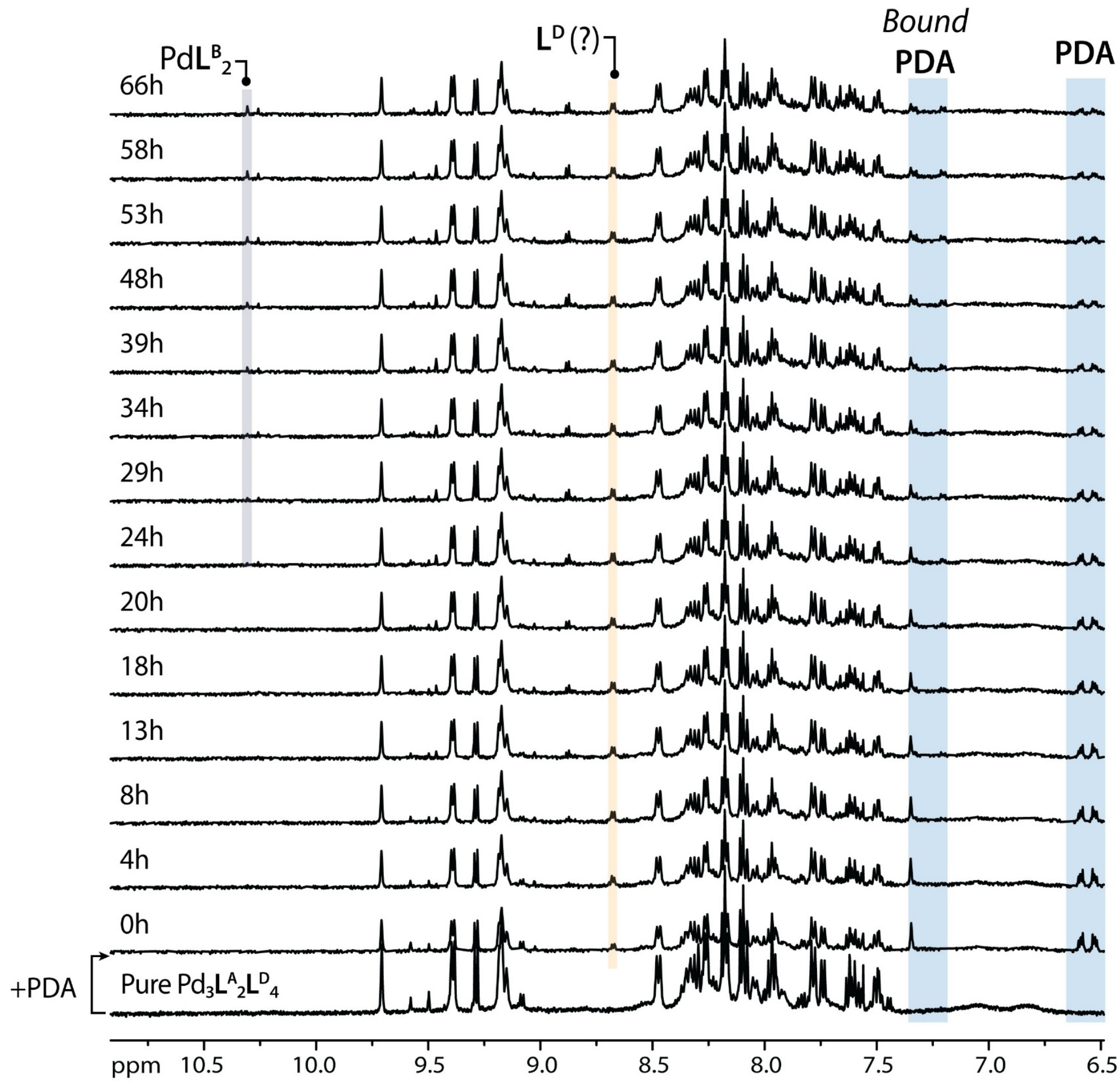

Figure S54. ^{1}H NMR (500 MHz, 298K, CD_3CN) monitoring of the reaction of $Pd_3\mathbf{L}^{\mathbf{A}}_2\mathbf{L}^{\mathbf{D}}_2$ (0.7 mM) with PDA (1.5 mM) at RT. The observed signals of $\mathbf{L}^{\mathbf{D}}$ are slightly shifted in comparison to the pure $\mathbf{L}^{\mathbf{D}}$ ligand, what may indicate its involvement in some chemical exchange or assembly.

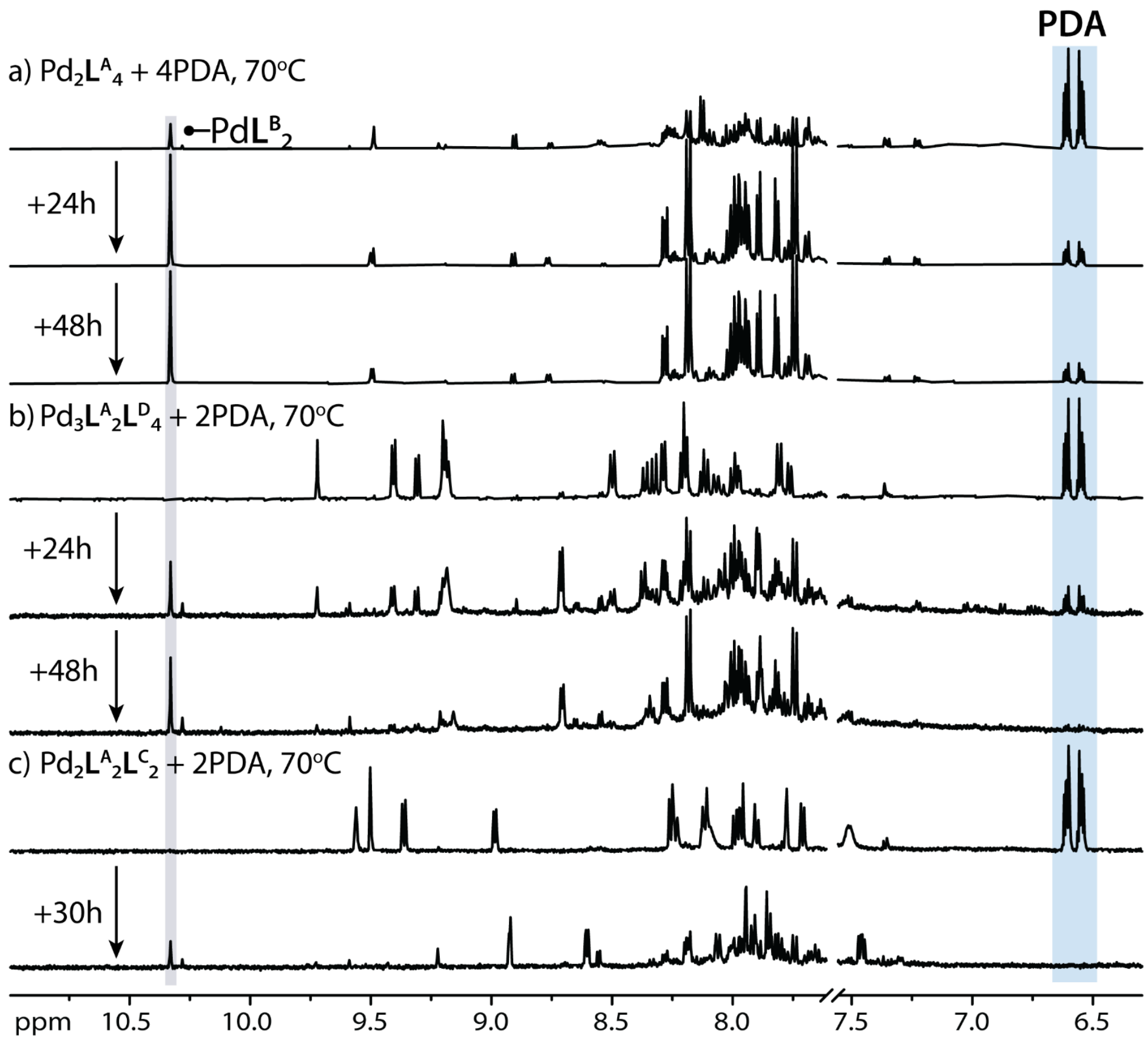

Figure S55. ^1H NMR (500 MHz, 298K, CD_3CN) monitoring of the reaction of a) $Pd_2\mathbf{L^A}_4$, b) $Pd_3\mathbf{L^A}_2\mathbf{L^D}_4$, and c) $Pd_2\mathbf{L^A}_2\mathbf{L^C}_2$ (0.7 mM) with PDA at 70 °C.

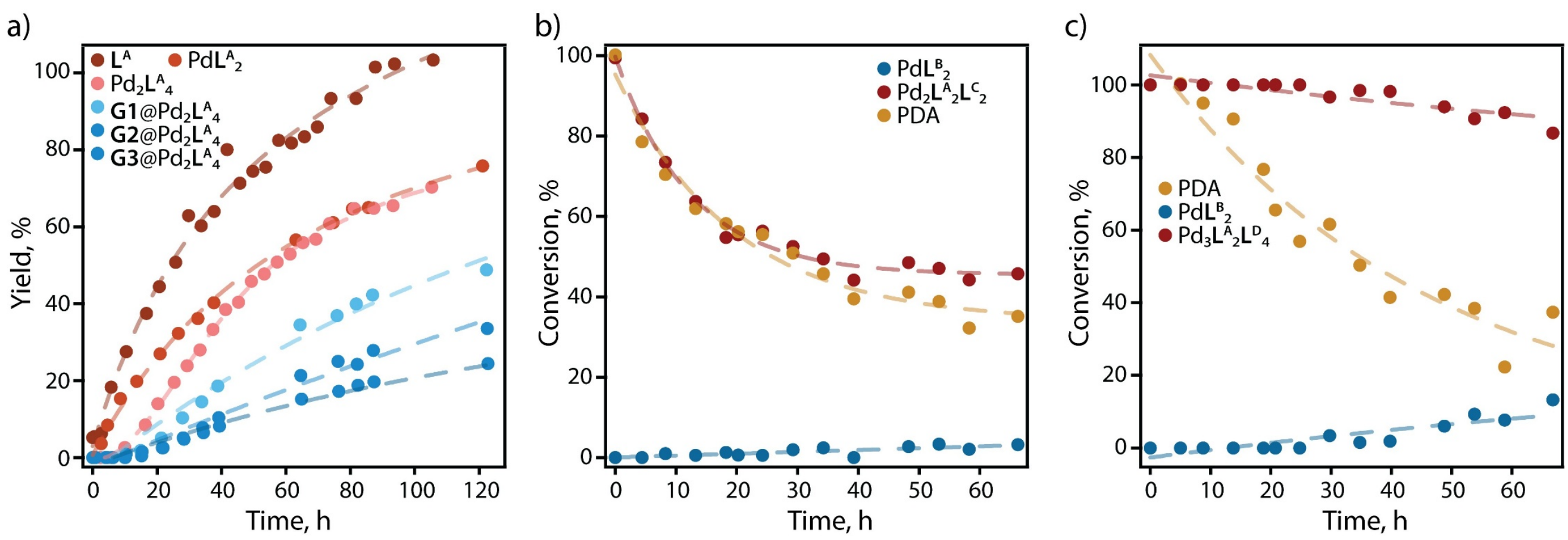

Figure S56. Plots: (a) condensation yields versus time for homoleptic systems at RT; (b) conversions of reactants and product yield versus time for $Pd_2\mathbf{L^A}_2\mathbf{L^C}_2$ condensation with PDA at RT; and (c) conversions of reactants and product yield versus time for $Pd_3\mathbf{L^A}_2\mathbf{L^D}_4$ condensation with PDA at RT. Yields and conversions were determined based on the initial concentration of $\mathbf{L^A}$ (free or as part of assembly) in solution prior to the addition of PDA.

S6. X-ray crystallography

S6.1 Crystal samples preparation

Colorless block crystals of Pd$\mathbf{L}^{\mathbf{B}}_2$ were grown by slow vapor diffusion of ethyl acetate into a solution of Pd$\mathbf{L}^{\mathbf{B}}_2$ in acetonitrile.

Colorless block crystals of $Pd_2$$\mathbf{L}^{\mathbf{A}}_4$ were grown by slow vapor diffusion of diethyl ether into a solution of $Pd_2$$\mathbf{L}^{\mathbf{A}}_4$ in acetonitrile.

Yellow plate crystals of **G2**@$Pd_2$$\mathbf{L}^{\mathbf{A}}_4$ were grown by slow vapor diffusion of diethyl ether into a solution of $Pd_2$$\mathbf{L}^{\mathbf{A}}_4$ in acetonitrile with 1 eq. of tetrabutylammonium propane-1,3-disulfonate.

Yellow plate crystals of **G3**@$Pd_2$$\mathbf{L}^{\mathbf{A}}_4$ were grown by slow vapor diffusion of diisopropyl ether into a solution of $Pd_2$$\mathbf{L}^{\mathbf{A}}_4$ in acetonitrile with 1 eq. of tetrabutylammonium benzene-1,4-disulfonate.

Colorless plate crystals of $Pd_2$$\mathbf{L}^{\mathbf{A}}_2$$\mathbf{L}^{\mathbf{C}}_2$ were grown by slow vapor diffusion of diethyl ether into a solution of $Pd_2$$\mathbf{L}^{\mathbf{A}}_2$$\mathbf{L}^{\mathbf{C}}_2$ in acetonitrile.

S6.2 X-ray data collection and refinement details

Data for Pd$\mathbf{L}^{\mathbf{B}}_2$ crystals were collected in-house on a Bruker D8 venture diffractometer equipped with an INCOATEC microfocus sealed tube (Iµs 3.0) using CuKα radiation at 100 K. The data was integrated with APEX3 software.

The crystals of $Pd_2$$\mathbf{L}^{\mathbf{A}}_4$, **G2**@$Pd_2$$\mathbf{L}^{\mathbf{A}}_4$, **G3**@$Pd_2$$\mathbf{L}^{\mathbf{A}}_4$, and $Pd_2$$\mathbf{L}^{\mathbf{A}}_2$$\mathbf{L}^{\mathbf{C}}_2$ were stored at cryogenic temperatures in dry shippers, which were used to safely transport them to the macromolecular synchrotron beamline P11 at PETRA III, DESY, Hamburg, Germany.

A wavelength (λ) of 0.6888 was selected using a liquid nitrogen-cooled double crystal monochromator. Single-crystal X-ray diffraction data were collected at 100(2) K on a single-axis goniometer equipped with an Oxford Cryostream 800 and an Eiger 2X 16M detector. A total of 3600 diffraction images were collected during a 360° φ sweep at a detector distance of 154 mm, with 100% filter transmission, a 0.1° step width, and an exposure time of 0.5 seconds per image. Data indexing, integration, and reduction were undertaken using autoPROC[74] and XDS[75] software.

All structures were solved by direct methods using SHELXT[76] and refined by full-matrix least-squares methods against F^2 by SHELXL[77] in the OLEX2 program package.[78,79] All non-hydrogen atoms were refined with anisotropic displacement parameters. The hydrogen atoms were refined

isotropically on calculated positions using a riding model. Crystal data and structure refinements are given in **Table S1–S3** and the selected geometrical parameters are given in **Table S4**.

Table S1. Crystal data and structure refinement for Pd$\mathbf{L^B}_2$ and $Pd_2$$\mathbf{L^A}_4$ crystal structures.

Name	Pd$\mathbf{L^B}_2$	$Pd_2$$\mathbf{L^A}_4$
CCDC code	2544681	2544682
Identification code	zz011-finalcif	zz110i-finalcif
Empirical formula	$C_{82}H_{57}B_2F_8N_{11}Pd$	$B_4C_{138}F_{16}N_{10}O_{9.5}Pd_2H_{99.5}$
Formula weight	1476.40	2609.81
Temperature/K	100.00	100(2)
Crystal system	monoclinic	triclinic
Space group	$P2_1/c$	P-1
a/Å	11.1372(7)	23.063(5)
b/Å	45.183(3)	23.357(5)
c/Å	13.2842(9)	24.871(5)
α/°	90	71.77(3)
β/°	95.804(3)	67.71(3)
γ/°	90	78.23(3)
Volume/Å^3	6650.5(8)	11719(5)
Z	4	4
ρ_{calc}g/cm^3	1.475	1.479
μ/mm^{-1}	2.922	0.364
F(000)	3016.0	5318.0
Crystal size/mm^3	0.05 × 0.05 × 0.02	0.075 × 0.03 × 0.01
Radiation	CuKα (λ = 1.54178)	synchrotron (λ = 0.6888)
2Θ range for data collection/°	3.91 to 158.058	1.776 to 56.234
Index ranges	$-13 \le h \le 13$, $-57 \le k \le 55$, $-16 \le l \le 16$	$-31 \le h \le 30$, $-30 \le k \le 30$, $-34 \le l \le 33$
Reflections collected	85485	154942
Independent reflections	14134 [R_{int} = 0.0814, R_{sigma} = 0.0443]	42054 [R_{int} = 0.0591, R_{sigma} = 0.0464]
Data/restraints/parameters	14134/0/940	42054/643/3360
Goodness-of-fit on F^2	1.019	1.045
Final R indexes [I>=2σ (I)]	R_1 = 0.0411, wR_2 = 0.1002	R_1 = 0.0658, wR_2 = 0.1812
Final R indexes [all data]	R_1 = 0.0554, wR_2 = 0.1083	R_1 = 0.0794, wR_2 = 0.2019
Largest diff. peak/hole / e Å^{-3}	0.88/-0.84	1.56/-1.62

Table S2. Crystal data and structure refinement for **G2**@Pd_2**L**$^A{}_4$ and **G3**@Pd_2**L**$^A{}_4$ crystal structures.

Name	**G2**@Pd_2**L**$^A{}_4$	**G3**@Pd_2**L**$^A{}_4$
CCDC code	2544684	2544683
Identification code	pgb122i-finalcif	pgb120j-finalcif
Empirical formula	$C_{143}H_{108}B_2F_8N_{12}O_{15}Pd_2S_2$	$C_{136}H_{87}B_2F_8N_9O_{14}Pd_2S_2$
Formula weight	2684.95	2521.68
Temperature/K	100	100
Crystal system	triclinic	monoclinic
Space group	P-1	P2/c
a/Å	13.860(3)	24.651(5)
b/Å	21.244(4)	21.206(4)
c/Å	22.401(5)	26.257(5)
α/°	87.24(3)	90
β/°	76.81(3)	117.36(3)
γ/°	72.49(3)	90
Volume/Å^3	6123(3)	12190(5)
Z	2	4
ρ_{calc}g/cm^3	1.456	1.374
μ/mm^{-1}	0.376	0.372
F(000)	2752.0	5136.0
Crystal size/mm^3	0.04 × 0.035 × 0.02	0.1 × 0.05 × 0.01
Radiation	synchrotron (λ = 0.6888)	synchrotron (λ = 0.6888)
2Θ range for data collection/°	1.81 to 52.404	2.516 to 56.07
Index ranges	-16 ≤ h ≤ 16, -26 ≤ k ≤ 26, -28 ≤ l ≤ 28	-32 ≤ h ≤ 33, -28 ≤ k ≤ 28, -34 ≤ l ≤ 34
Reflections collected	78795	155567
Independent reflections	21201 [R_{int} = 0.0409, R_{sigma} = 0.0337]	25502 [R_{int} = 0.0241, R_{sigma} = 0.0157]
Data/restraints/parameters	21201/244/1728	25502/478/1799
Goodness-of-fit on F^2	1.072	1.045
Final R indexes [I>=2σ (I)]	R_1 = 0.0825, wR_2 = 0.2427	R_1 = 0.0684, wR_2 = 0.2101
Final R indexes [all data]	R_1 = 0.1011, wR_2 = 0.2601	R_1 = 0.0727, wR_2 = 0.2167
Largest diff. peak/hole / e Å^{-3}	1.93/-0.69	1.78/-1.72

Table S3. Crystal data and structure refinement for $Pd_2\mathbf{L^A}_2\mathbf{L^C}_2$ crystal structure.

Name	$Pd_2\mathbf{L^A}_2\mathbf{L^C}_2$
CCDC code	2544685
Identification code	pgb90i-finalcif
Empirical formula	$C_{116}H_{77}B_4F_{16}N_{11}O_6Pd_2$
Formula weight	2280.92
Temperature/K	100
Crystal system	triclinic
Space group	P-1
a/Å	13.155(3)
b/Å	19.237(4)
c/Å	22.312(5)
α/°	94.53(3)
β/°	107.02(3)
γ/°	97.39(3)
Volume/Å^3	5314(2)
Z	2
ρ_{calc}g/cm^3	1.426
μ/mm^{-1}	0.389
F(000)	2308.0
Crystal size/mm^3	0.1 × 0.05 × 0.01
Radiation	synchrotron (λ = 0.6888)
2Θ range for data collection/°	1.864 to 52.392
Index ranges	-16 ≤ h ≤ 16, -24 ≤ k ≤ 24, -25 ≤ l ≤ 25
Reflections collected	50895
Independent reflections	18237 [R_{int} = 0.0594, R_{sigma} = 0.0637]
Data/restraints/parameters	18237/910/1583
Goodness-of-fit on F^2	1.043
Final R indexes [I>=2σ (I)]	R_1 = 0.0905, wR_2 = 0.2554
Final R indexes [all data]	R_1 = 0.1307, wR_2 = 0.2913
Largest diff. peak/hole / e Å^{-3}	1.52/-0.79

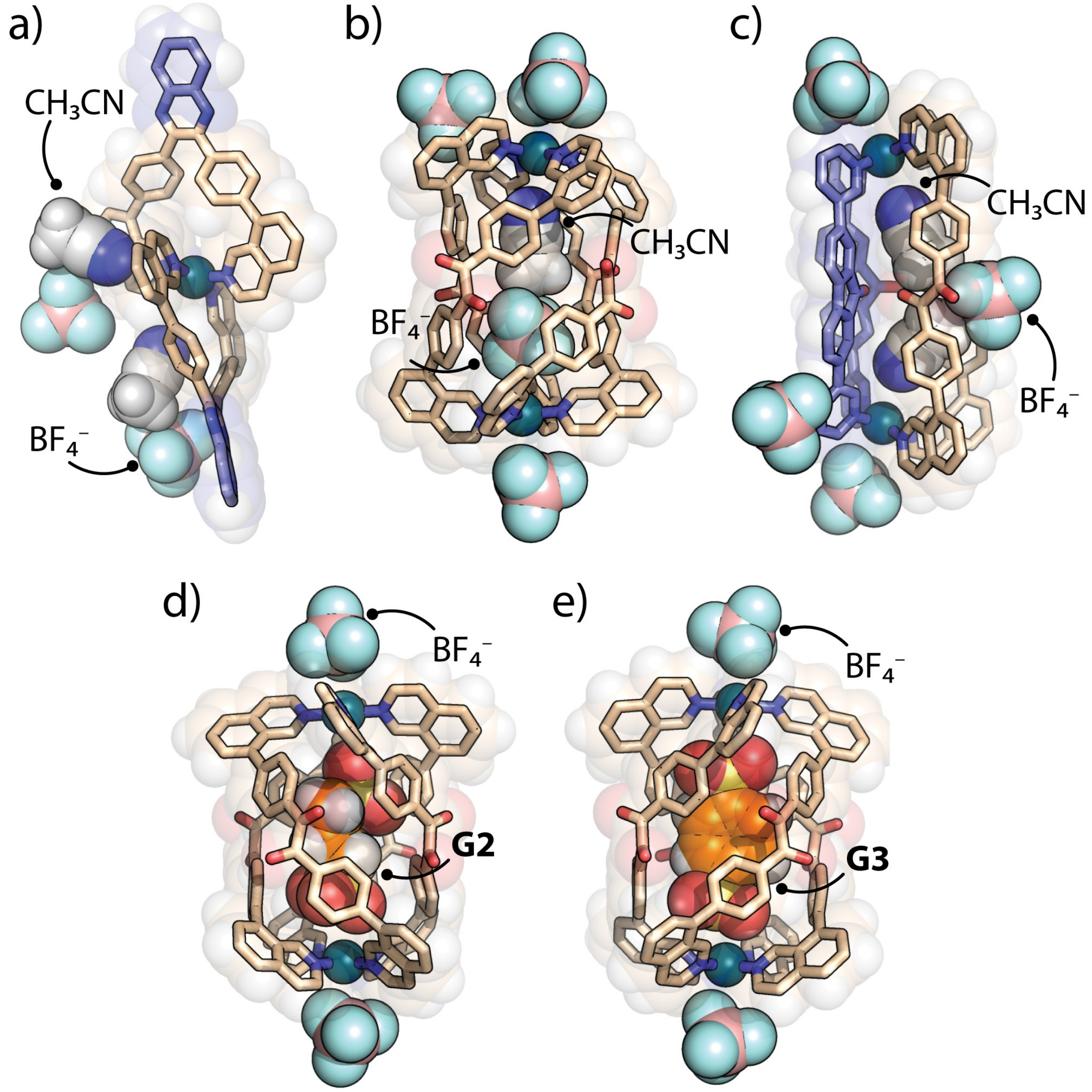

Figure S57. Views of crystal structures of (a) Pd$\mathbf{L}^{\mathbf{B}}_2$, (b) $Pd_2$$\mathbf{L}^{\mathbf{A}}_4$, (c) $Pd_2$$\mathbf{L}^{\mathbf{A}}_2$$\mathbf{L}^{\mathbf{C}}_2$, (d) **G2**@$Pd_2$$\mathbf{L}^{\mathbf{A}}_4$, and (e) **G3**@$Pd_2$$\mathbf{L}^{\mathbf{A}}_4$ with corresponding counter-ions and guest molecules.

S7. DFT calculations

S7.1 Calculations details

The geometry optimization and single-point energy calculations of the studied cages and ligands were conducted using the ORCA 6.0 software.[80] The initial structures of cages and ligands were built in ChemCraft software[81] and pre-optimized at the GFN2-xTB[82]/ALPB[83](CH_3CN) level using the xTB software.[84] The structures of ligands and cages were further optimized at the r^{2}SCAN-3c[62,main text] level using the CPCM(CH_3CN) (or DMSO in case of half-helical Pd_2**L**$^{\mathbf{A}}_4$ cage) solvation model.[63,main text] For homoleptic Pd_nL_{2n} assemblies with ligand **L**$^{\mathbf{C}}$ and **L**$^{\mathbf{D}}$ the Pd_3**L**$^{\mathbf{C}}_6$ and Pd_4**L**$^{\mathbf{D}}_8$ ring structures were selected as the model systems as these topologies were reported as major assembly products in the acetonitrile. [39,main text, 69] To find lowest energy conformer of **L**$^{\mathbf{A}}$, the Global Optimizer Algorithm (GOAT)[85] in ORCA 6.0 was utilized at r^{2}SCAN-3c/CPCM(CH_3CN) level. The views of the optimized geometries of the studied species are given in **Figure S58** and the selected geometrical parameters are given in **Table S4**, while the Cartesian atomic coordinates for the optimized model structures are provided as supplementary XYZ files. Conformational analysis of 1,2-diphenylethane-1,2-dione was performed using relaxed scan calculations of the dihedral angle between the *para*-carbon atoms of the phenyl rings at the r^{2}SCAN-3c/CPCM(CH_3CN) level. The dihedral angle varied from 0° to 360° and from 0° to −360° with step of 5° to ensure access to all local minimum structures.

Final single-point energy calculations for Pd(II) assemblies and ligand conformations were carried out at the ωB97X-D4/def2TZVPP/SMD-DRACO(CH_3CN),[64-68,main text] while thermostatistical corrections (G_{TRV}) for cage structures using the modified rigid rotor harmonic oscillator (mRRHO) approach[69,main text] were obtained from harmonic frequency calculations at the GFN2-xTB/ALPB(CH_3CN) level using the xTB software. The obtained energies and their differences are given in **Tables S5**–**S6**.

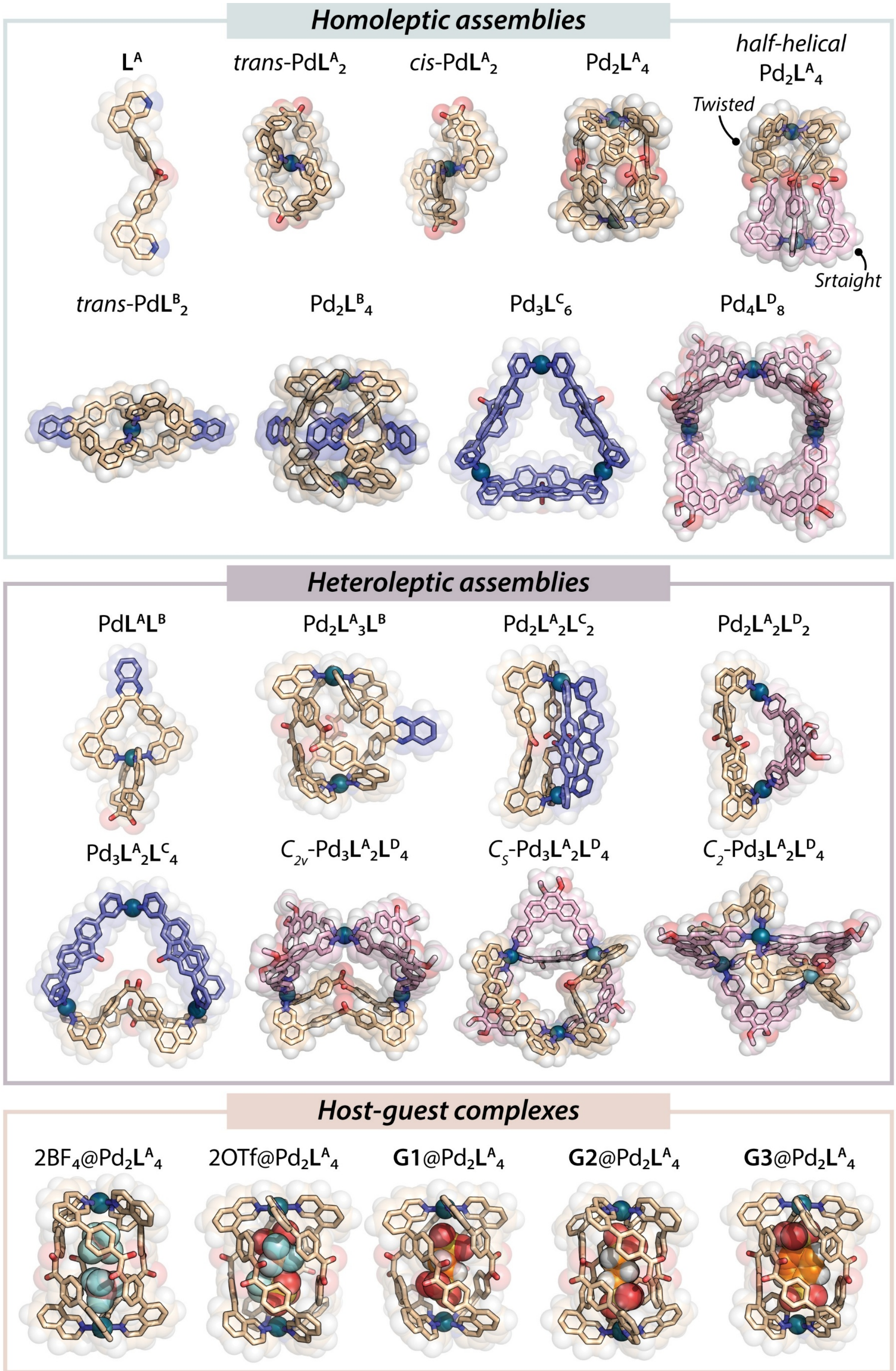

Figure S58. Views of the DFT optimized geometries of the studied species.

S7.2 Assemblies relative stability

To understand the relationship between the structures of homoleptic and heteroleptic Pd(II) assemblies and their stability, theoretical calculations of their structures and energies were performed. All structures were modeled without counter-ions to simplify the calculations.

In the case of the homoleptic $\mathbf{L^A}$ assemblies, the helicate $Pd_2\mathbf{L^A}_4$ cage is more stable than the mononuclear *trans*- and *cis*-$Pd\mathbf{L^A}_2$ complexes by 54 and 89 kJ/mol, respectively. At the same time, the *trans*-$Pd\mathbf{L^A}_2$ isomer is approximately 35 kJ/mol more energetically favorable than the cis isomer (Table S6). Conversely, for $\mathbf{L^B}$ assemblies, formation of the mononuclear *trans*-$Pd\mathbf{L^B}_2$ structure is more favorable than formation of the $Pd_2\mathbf{L^B}_4$ cage, likely due to the larger ligand strain energy (+13 kJ/mol, **Table S6**) associated with the rigid quinoxaline backbone in the cage structure compared to the mononuclear species.

For heteroleptic assemblies, the binuclear $Pd_2\mathbf{L^A}_2\mathbf{L^C}_2$ complex is significantly more stable than the trinuclear $Pd_3\mathbf{L^A}_2\mathbf{L^C}_4$ species by −188 kJ/mol for the transformation $3 \times Pd_3\mathbf{L^A}_2\mathbf{L^C}_4 \rightleftharpoons 3 \times Pd_2\mathbf{L^A}_2\mathbf{L^C}_2 + Pd_3\mathbf{L^C}_6$ (Figure S56 and Table S6). In contrast, $Pd_3\mathbf{L^A}_2\mathbf{L^D}_4$ is more stable than the binuclear heteroleptic $Pd_2\mathbf{L^A}_2\mathbf{L^D}_2$ cage by −8 kJ/mol for the transformation $4 \times Pd_2\mathbf{L^A}_2\mathbf{L^D}_2 + Pd_4\mathbf{L^D}_8 \rightleftharpoons 4 \times Pd_3\mathbf{L^A}_2\mathbf{L^D}_4$ (**Figure S59** and **Table S6**). However, this applies only to the C_{2v}-symmetric $Pd_3\mathbf{L^A}_2\mathbf{L^D}_4$ isosceles triangular structure, which is the most energetically favorable among all $Pd_3\mathbf{L^A}_2\mathbf{L^D}_4$ isomers (by −53 and −73 kJ/mol). Considering the relative stability of heteroleptic assemblies with different ligands, the C_{2v}-symmetric $Pd_3\mathbf{L^A}_2\mathbf{L^D}_4$ assembly should be more favorable than $Pd_2\mathbf{L^A}_2\mathbf{L^C}_2$ by 55 kJ/mol for the transformation $6 \times Pd_3\mathbf{L^A}_2\mathbf{L^D}_4 + 2 \times Pd_3\mathbf{L^C}_6 \rightleftharpoons 6 \times Pd_2\mathbf{L^A}_2\mathbf{L^C}_2 + 3 \times Pd_4\mathbf{L^D}_8$.

Considering the condensation of the $Pd_2\mathbf{L^A}_4$ assemblies with **PDA**, the calculations indicate that the formation of single $\mathbf{L^B}$ within the $Pd_2\mathbf{L^A}_4$ cage (yielding heteroleptic $Pd_2\mathbf{L^A}_3\mathbf{L^B}$ cage) should introduce significant strain (**Figure S60**) on the structure. While the $Pd_2\mathbf{L^A}_3\mathbf{L^B}$ cage structure is still on 13 kJ/mol more stable than combination of corresponding mononuclear $Pd\mathbf{L^A}\mathbf{L^B}$ and $Pd\mathbf{L^A}_2$, under the reaction condition and low concertation of generated intermediate species the mononuclear complexes $Pd\mathbf{L^A}\mathbf{L^B}$ and $Pd\mathbf{L^A}_2$ should dominate in quantity over $Pd_2\mathbf{L^A}_3\mathbf{L^B}$ cage structure.

Overall, the obtained results agree well with the experimental observations.

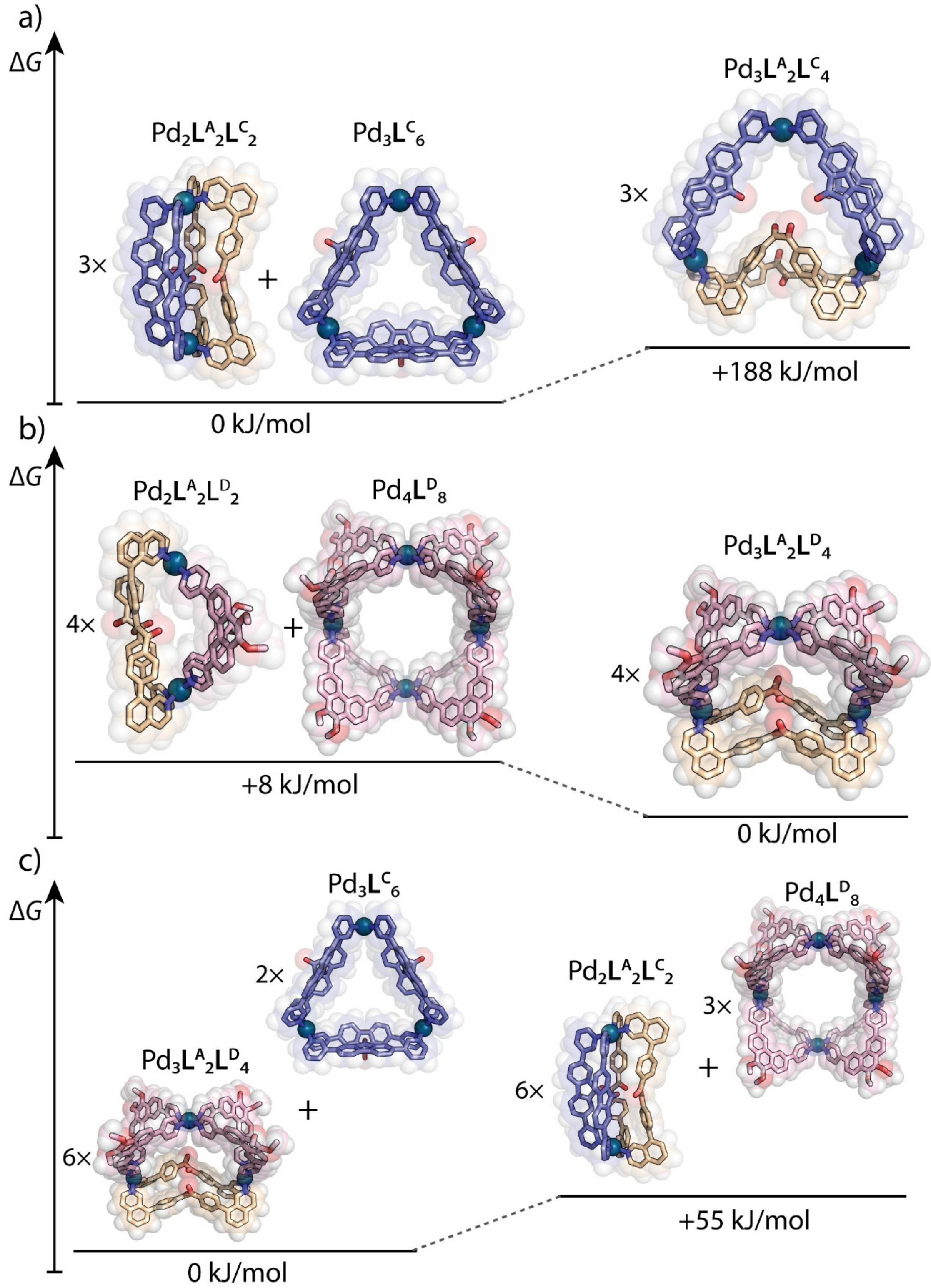

Figure S59. Calculated free energy differences between combination homo- and heteroletic cages corresponding to possible cage-to-cage transformations.

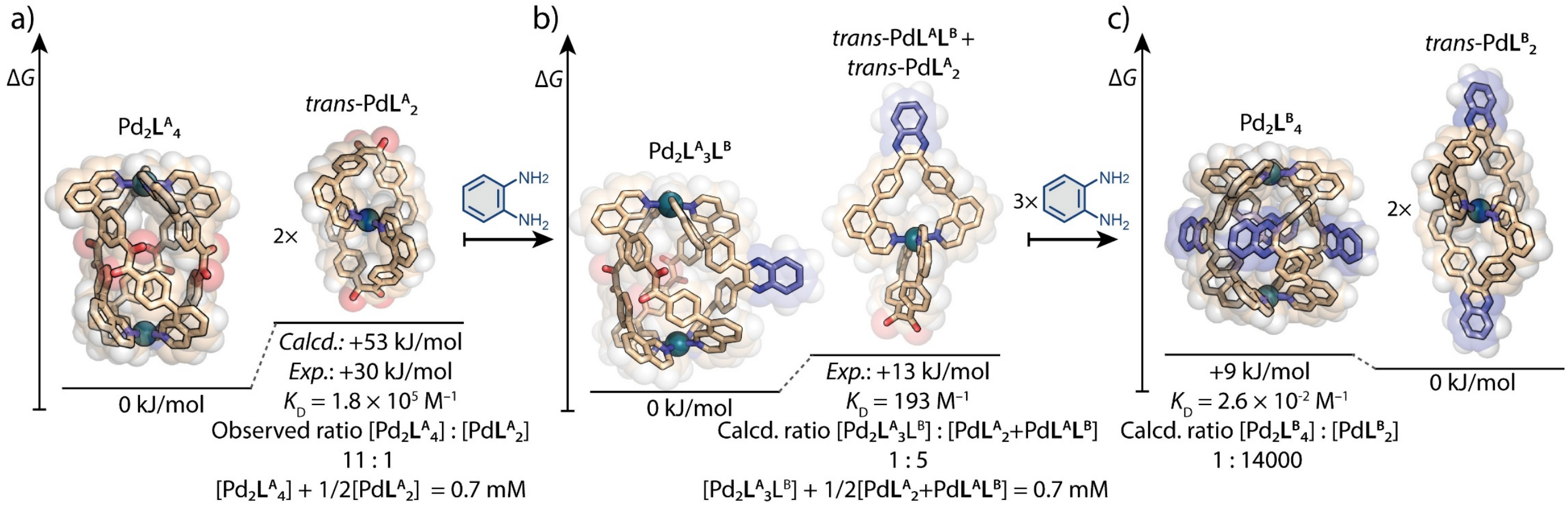

Figure S60. Calculated free energy differences, dimerization constants and expected component ratio for (a) homoleptic Pd_2**L**$^{\mathbf{A}}{}_4$ and Pd**L**$^{\mathbf{A}}{}_2$, heteroleptic Pd_2**L**$^{\mathbf{A}}{}_3$**L**$^{\mathbf{B}}$ and Pd**L**$^{\mathbf{A}}{}_2$+Pd**L**$^{\mathbf{A}}$**L**$^{\mathbf{B}}$, and (c) homoleptic Pd_2**L**$^{\mathbf{B}}{}_4$ and Pd**L**$^{\mathbf{B}}{}_2$.

S7.3 Guest binding energies

To evaluate the affinity of different guests for the Pd_2**L**$^{\mathbf{A}}{}_4$ cage, calculations of the structures and energies of the free Pd_2**L**$^{\mathbf{A}}{}_4$ cage, the anionic guest – tetrafluoroborate (BF_4^-), triflate (OTf^-), ethane-1,2-disulfonate (**G1**), propane-1,3-disulfonate (**G2**), and benzene-1,4-disulfonate (**G3**), and their corresponding host–guest complexes were performed. For the host–guest complexes with $BF4^-$ and OTf^-, two guest molecules were modeled inside the cage to allow comparison with the host–guest complexes containing the dianionic **G1**–**G3** guests.

Guest affinity was evaluated by subtracting the energies of the free host and guests from the energy of the host–guest complex: $E_{bind} = E(n\mathbf{G}@Pd_2\mathbf{L^A}_4) - [E(Pd_2\mathbf{L^A}_4) + n \times E(\mathbf{G})]$ (**Table S6**).

According to the DFT calculations, the following guest affinity order for the Pd_2**L**$^{\mathbf{A}}{}_4$ cage was obtained: $BF4^-$ < OTf^- < **G1** < **G2** < **G3**. This trend correlates with guest size and is in good agreement with the experimental results.

S7.4 Ligand conformational analysis

To study the conformational flexibility of **L**$^{\mathbf{A}}$ and the effect of ligand conformation on the structure and properties of the Pd(II) assemblies, a relaxed scan of the dihedral angle between the phenyl arms of the benzil as model compound was performed. Energy minima were observed at dihedral angles of 105° and 250°, whereas energy maxima occurred at dihedral angles of 180° (+19.5 kJ/mol) and 360° (+31 kJ/mol). In both cases, benzil experiences steric repulsion, either between the phenylene moieties in the fully folded *syn*-conformation or between the phenylene and carbonyl moieties in the *anti*-conformation.

The conformation adopted by $\mathbf{L^A}$ in the optimized structure of the free ligand (dihedral angle of 253°) lies close to the calculated energy minima of 1,2-diphenylethane-1,2-dione. Despite the similar dihedral angles of $\mathbf{L^A}$ in the $Pd_3\mathbf{L^A}_2\mathbf{L^D}_4$ cage (238° and 261°, **Table S4**), these conformations are less favorable than that of the free ligand by approximately 7.2–8.9 kJ/mol (**Table S6**). The latter can be explained by the additional strain in the isoquinoline and phenylene moieties caused by adjusting to the cage structure. The open conformation in $Pd_2\mathbf{L^A}_4$ is, as expected, even more strained relative to the free ligand, with a strain energy of 12.9 kJ/mol. Even higher strain energies are observed for $\mathbf{L^A}$ in $Pd\mathbf{L^A}_2$ and $Pd_2\mathbf{L^A}_2\mathbf{L^C}_2$, with ΔE_{strain} values of 14.5 and 16.7 kJ/mol, respectively (**Table S6**).

Table S4. Selected geometrical parameters in crystal and optimized structures of studied systems.

Species	Structure	Pd•••Pd, Å	Dicarbonyl dihedral angle, (°)
$\mathbf{L}^{\mathbf{A}}$	DFT Model	–	252.4
trans-Pd$\mathbf{L}^{\mathbf{A}}_2$	DFT Model	–	70.2/70.3
$Pd_2\mathbf{L}^{\mathbf{A}}_4$	X-ray	13.57<br>13.67	124.8/134.9/136.4/140.5<br>125.1/127.4/129.2/136.3
	DFT Model	13.57	125.9/126.3/126.6/127.4
$2BF_4$@$Pd_2\mathbf{L}^{\mathbf{A}}_4$	DFT Model	13.49	127.7/127.9/128.7/129.1
2OTf@$Pd_2\mathbf{L}^{\mathbf{A}}_4$	DFT Model	13.18	119.4/124.1/124.3/129.7
**G1**@$Pd_2\mathbf{L}^{\mathbf{A}}_4$	DFT Model	12.78	110.5/114.4/122.7/127.1
**G2**@$Pd_2\mathbf{L}^{\mathbf{A}}_4$	X-ray	13.02	128.4/130.8/131.0/
	DFT Model	13.53	128.1/129.5/129.5/130.4/131.4
**G3**@$Pd_2\mathbf{L}^{\mathbf{A}}_4$	X-ray	13.44/13.57	129.4/131.3/131.3/138.3
	DFT Model	13.69	124.0/134.6/128.7/130.1
$Pd_2\mathbf{L}^{\mathbf{A}}_2\mathbf{L}^{\mathbf{C}}_2$	X-ray	14.41	188.0/190.0
	DFT Model	14.44	210.2/211.6
$Pd_3\mathbf{L}^{\mathbf{A}}_2\mathbf{L}^{\mathbf{D}}_4$	DFT Model	11.94/12.13/16.00	237.5/260.7

Table S5. Diffusion coefficient (*D*) obtained by ^1H DOSY technique and calculated hydrodynamic radii (r_H) for studied assemblies.

Species	Solvent	$D\times10^{10}$, m^2/s	r_H, Å
$[Pd\mathbf{L}^{\mathbf{A}}_2](BF_4)_4$	CD_3CN	8.36±0.26	7.85±0.25
$[Pd\mathbf{L}^{\mathbf{A}}_2](OTf)_4$		7.91±0.34	8.32±0.35
$[Pd_2\mathbf{L}^{\mathbf{A}}_4](BF_4)_4$	CD_3CN	6.64±0.09	9.85±0.13
	CD_3NO_2	3.55±0.04	9.51±0.11
	DMSO-d_6	9.83±0.13	11.16±0.14
$[Pd_2\mathbf{L}^{\mathbf{A}}_4](OTf)_4$	CD_3CN	6.28±0.02	10.43±0.03
[**G2**@$Pd_2\mathbf{L}^{\mathbf{A}}_4$]$(BF_4)_2$		6.80±0.04	9.62±0.06
[**G3**@$Pd_2\mathbf{L}^{\mathbf{A}}_4$]$(BF_4)_2$		6.70±0.02	9.77±0.03
$[Pd_2\mathbf{L}^{\mathbf{A}}_2\mathbf{L}^{\mathbf{C}}_2](BF_4)_4$		6.15±0.05	10.64±0.09
$[Pd_3\mathbf{L}^{\mathbf{A}}_2\mathbf{L}^{\mathbf{D}}_4](BF_4)_6$		5.12±0.04	12.78±0.10
$[Pd\mathbf{L}^{\mathbf{B}}_2](BF_4)_4$		7.49±0.04	8.73±0.05

Table S6. Calculated energies (Harteree) for the studied cages, guests, and host-guest complexes.

Compound	E_{scf}	E_{disp}	E_{tot}	G_{TRV}	G
Homoleptic assemblies					
cis-Pd$\mathbf{L}^{\mathbf{A}}_{2}$	-3110.89909	-0.04267	-3110.94176	0.74977	-3110.19199
trans-Pd$\mathbf{L}^{\mathbf{A}}_{2}$	-3110.91307	-0.04262	-3110.95569	0.75042	-3110.20527
$Pd_2\mathbf{L}^{\mathbf{A}}_{4}$	-6221.86031	-0.10441	-6221.96472	1.53346	-6220.43126
trans-Pd$\mathbf{L}^{\mathbf{B}}_{2}$	-3491.15938	-0.04956	-3491.20894	0.90729	-3490.30165
$Pd_2\mathbf{L}^{\mathbf{B}}_{4}$	-6982.31758	-0.12724	-6982.44482	1.84496	-6980.59986
$Pd_3\mathbf{L}^{\mathbf{C}}_{6}$	-6801.67758	-0.09478	-6801.77237	1.63674	-6800.13563
$Pd_4\mathbf{L}^{\mathbf{D}}_{8}$	-10614.20457	-0.13551	-10614.34008	2.83743	-10611.50264
Heteroleptic assemblies					
$Pd\mathbf{L}^{\mathbf{A}}\mathbf{L}^{\mathbf{B}}$	-3301.03604	-0.04591	-3301.08196	0.82772	-3300.25424
$Pd_2\mathbf{L}^{\mathbf{A}}_{3}\mathbf{L}^{\mathbf{B}}$	-6411.96682	-0.10886	-6412.07569	1.61121	-6410.46447
$Pd_2\mathbf{L}^{\mathbf{A}}_{2}\mathbf{L}^{\mathbf{C}}_{2}$	-5378.14287	-0.08052	-5378.22339	1.30826	-5376.91513
$Pd_3\mathbf{L}^{\mathbf{A}}_{2}\mathbf{L}^{\mathbf{C}}_{4}$	-7645.34863	-0.11805	-7645.46668	1.86357	-7643.60311
$Pd_2\mathbf{L}^{\mathbf{A}}_{2}\mathbf{L}^{\mathbf{D}}_{2}$	-5764.46845	-0.08107	-5764.54952	1.46783	-5763.08168
C_{2v}-$Pd_3\mathbf{L}^{\mathbf{A}}_{2}\mathbf{L}^{\mathbf{D}}_{4}$	-8418.02864	-0.11390	-8418.14253	2.18444	-8415.95810
C_{s}-$Pd_3\mathbf{L}^{\mathbf{A}}_{2}\mathbf{L}^{\mathbf{D}}_{4}$	-8418.00738	-0.11657	-8418.12394	2.18585	-8415.93809
C_{2}-$Pd_3\mathbf{L}^{\mathbf{A}}_{2}\mathbf{L}^{\mathbf{D}}_{4}$	-8417.99269	-0.12273	-8418.11541	2.18517	-8415.93024
Guests					
BF_4^{-}	-424.69967	-0.00030	-424.69997	-0.01273	-424.71270
OTf^{-}	-961.79123	-0.00114	-961.79236	-0.00734	-961.79970
$\mathbf{G1}^{2-}$	-1326.79997	-0.00271	-1326.80268	0.04186	-1326.80539
$\mathbf{G2}^{2-}$	-1366.11901	-0.00318	-1366.12219	0.06791	-1366.05428
$\mathbf{G3}^{2-}$	-1479.22592	-0.00438	-1479.23030	0.06194	-1479.16837
Host-guest complexes					
$2BF_4@Pd_2\mathbf{L}^{\mathbf{A}}_{4}$	-7071.26125	-0.11582	-7071.37707	1.54593	-7069.83115
$2OTf@Pd_2\mathbf{L}^{\mathbf{A}}_{4}$	-8145.45622	-0.12684	-8145.58306	1.56559	-8144.01747
$\mathbf{G1}@Pd_2\mathbf{L}^{\mathbf{A}}_{4}$	-7548.69961	-0.12683	-7548.82644	1.59890	-7547.22754
$\mathbf{G2}@Pd_2\mathbf{L}^{\mathbf{A}}_{4}$	-7588.01971	-0.12717	-7588.14689	1.62598	-7586.52090
$\mathbf{G3}@Pd_2\mathbf{L}^{\mathbf{A}}_{4}$	-7701.12830	-0.13196	-7701.26026	1.62229	-7699.63797
L conformations					
Free $\mathbf{L}^{\mathbf{A}}$	-1491.58582	-0.01464	-1491.60047	–	–
trans-Pd$\mathbf{L}^{\mathbf{A}}_{2}$	-1491.57839	-0.01572	-1491.59411	–	–
$Pd_2\mathbf{L}^{\mathbf{A}}_{4}$	-1491.58102	-0.01455	-1491.59557	–	–
$Pd_2\mathbf{L}^{\mathbf{A}}_{2}\mathbf{L}^{\mathbf{C}}_{2}$	-1491.58043	-0.01451	-1491.59493	–	–
$Pd_3\mathbf{L}^{\mathbf{A}}_{2}\mathbf{L}^{\mathbf{D}}_{4}$	-1491.58242	-0.01466	-1491.59709	–	–
	-1491.58314	-0.01459	-1491.59772	–	–
Free $\mathbf{L}^{\mathbf{B}}$	-1681.70489	-0.01986	-1681.724757	–	–
trans-Pd$\mathbf{L}^{\mathbf{B}}_{2}$	-1681.70200	-0.01945	-1681.721453	–	–
$Pd_2\mathbf{L}^{\mathbf{B}}_{4}$	-1681.69761	-0.01880	-1681.716402	–	–

$E_{disp.}$: Dispersion correction energy; G_{TRV}: thermostatistical correction;
$E_{tot} = E_{scf} + E_{disp}$; $G = E_{tot} + G_{TRV}$

Table S7. Calculated selected energy differences (kJ/mol) for estimation of relative stability of isomers, homoleptic and heteroleptic assemblies, and host-guest complexes.

Compound	ΔE_{scf}	ΔE_{disp}	ΔE_{tot}	ΔG_{TRV}	ΔG

***Isomer relative stability**: $E(trans) - E(cis)$*					
$Pd\mathbf{L}^{\mathbf{A}}_2$	-36.7	0.1	-36.6	1.7	-34.9
***Assembly relative stability**: $E(Pd_2\mathbf{L}_4) - 2\times E(Pd\mathbf{L}_2)$*					
$\mathbf{L^A}$	-89.7	-50.3	-140.0	85.6	-54.4
$\mathbf{L^B}$	3.1	-73.8	-70.7	79.7	9.0
***Topology relative stability**: $E(C_{2v}\text{-}Pd_3\mathbf{L}^{\mathbf{A}}_2\mathbf{L}^{\mathbf{D}}_4) - E(C_x\text{-}Pd_3\mathbf{L}^{\mathbf{A}}_2\mathbf{L}^{\mathbf{D}}_4)$*					
$C_s\text{-}Pd_3\mathbf{L}^{\mathbf{A}}_2\mathbf{L}^{\mathbf{D}}_4$	55.8	-7.0	48.8	3.7	52.5
$C_2\text{-}Pd_3\mathbf{L}^{\mathbf{A}}_2\mathbf{L}^{\mathbf{D}}_4$	94.4	-23.2	71.2	1.9	73.1
***Cage-to-cage transformation energy**: $E(Pd_2\mathbf{L}^{\mathbf{A}}_3\mathbf{L}^{\mathbf{B}}) - ((E(Pd\mathbf{L}^{\mathbf{A}}\mathbf{L}^{\mathbf{B}}) + E(Pd\mathbf{L}^{\mathbf{A}}_2))$*					
-	-46.5	-53.4	-99.9	86.8	-13.0
***Cage-to-cage transformation energy**: $3\times E(Pd_3\mathbf{L}^{\mathbf{A}}_2\mathbf{L}^{\mathbf{C}}_4) - ((3\times E(Pd_2\mathbf{L}^{\mathbf{A}}_2\mathbf{L}^{\mathbf{C}}_2) + E(Pd_3\mathbf{L}^{\mathbf{C}}_6))$*					
-	158.3	-46.7	111.6	76.6	188.2
***Cage-to-cage transformation energy**: $4\times E(Pd_3\mathbf{L}^{\mathbf{A}}_2\mathbf{L}^{\mathbf{D}}_4) - (4\times E(Pd_2\mathbf{L}^{\mathbf{A}}_2\mathbf{L}^{\mathbf{D}}_2) + E(Pd_4\mathbf{L}^{\mathbf{D}}_8))$*					
**-**	-95.0	11.0	-21.0	76.1	-7.9
***Cage-to-cage transformation energy**:* $(6\times E(Pd_3\mathbf{L}^{\mathbf{A}}_2\mathbf{L}^{\mathbf{D}}_4) + 2\times E(Pd_3\mathbf{L}^{\mathbf{C}}_6)) - (6\times E(Pd_2\mathbf{L}^{\mathbf{A}}_2\mathbf{L}^{\mathbf{C}}_2) + 3\times E(Pd_4\mathbf{L}^{\mathbf{D}}_8))$					
-	-147.1	43.9	-103.3	47.9	-55.4
***Guest binding energy**: $E(n\mathbf{G}@Pd_2\mathbf{L}^{\mathbf{A}}_4) - (E(Pd_2\mathbf{L}_4) + n\times E(\mathbf{G}))$*					
$2BF_4@Pd_2\mathbf{L}^{\mathbf{A}}_4$	-4.2	-28.4	-32.6	99.6	67.0
$2OTf@Pd_2\mathbf{L}^{\mathbf{A}}_4$	-35.3	-52.9	-88.2	122.9	34.6
**G1**$@Pd_2\mathbf{L}^{\mathbf{A}}_4$	-103.2	-51.7	-155.0	61.9	23.9
**G2**$@Pd_2\mathbf{L}^{\mathbf{A}}_4$	-106.0	-51.4	-157.4	64.6	-92.8
**G3**$@Pd_2\mathbf{L}^{\mathbf{A}}_4$	-110.4	-60.8	-171.2	70.6	-100.6
***Ligand strain energy**: $E(\mathbf{L}_{assembly}) - E(\mathbf{L}_{free})$*					
trans-$Pd\mathbf{L}^{\mathbf{A}}_2$	19.5	-2.8	16.7	–	–
$Pd_2\mathbf{L}^{\mathbf{A}}_4$	12.6	0.3	12.9	–	–
$Pd_2\mathbf{L}^{\mathbf{A}}_2\mathbf{L}^{\mathbf{C}}_2$	14.1	0.4	14.5	–	–
$Pd_3\mathbf{L}^{\mathbf{A}}_2\mathbf{L}^{\mathbf{D}}_4$	8.9	0.0	8.9	–	–
	7.0	0.2	7.2	–	–
trans-$Pd\mathbf{L}^{\mathbf{B}}_2$	7.6	1.1	8.7	–	–
$Pd_2\mathbf{L}^{\mathbf{B}}_4$	19.1	2.8	21.9	–	–
$\Delta E_{disp.}$: Dispersion correction energy difference; ΔG_{TRV}: thermostatistical correction difference; $\Delta E_{tot} = \Delta E_{scf} + \Delta E_{disp}$; $\Delta G = \Delta E_{tot} + \Delta G_{TRV}$					

S8. References

[73] W. M. Bloch, Y. Abe, J. J. Holstein, C. M. Wandtke, B. Dittrich, G. H. Clever, "Geometric Complementarity in Assembly and Guest Recognition of a Bent Heteroleptic cis-[Pd2LA2LB2] Coordination Cage" *Journal of the American Chemical Society* 138 (2016): 13750–13755, https://doi.org/10.1021/jacs.6b08694.

[74] A. Jerschow, N. Müller, "3D Diffusion-Ordered TOCSY for Slowly Diffusing Molecules" *Journal of Magnetic Resonance, Series A* 123 (1996): 222–225, https://doi.org/10.1006/jmra.1996.0241.

[75] A. Jerschow, N. Müller, "Suppression of Convection Artifacts in Stimulated-Echo Diffusion Experiments. Double-Stimulated-Echo Experiments" *Journal of Magnetic Resonance* 125 (1997): 372–375, https://doi.org/10.1006/jmre.1997.1123.

[76] E. O. Stejskal, J. E. Tanner, "Spin Diffusion Measurements: Spin Echoes in the Presence of a Time-Dependent Field Gradient" *The Journal of Chemical Physics* 42 (1965): 288–292, https://doi.org/10.1063/1.1695690.

[77] J. E. Tanner, E. O. Stejskal, "Restricted Self-Diffusion of Protons in Colloidal Systems by the Pulsed-Gradient, Spin-Echo Method" *The Journal of Chemical Physics* 49 (1968): 1768–1777, https://doi.org/10.1063/1.1670306.

[78] C. Vonrhein, C. Flensburg, P. Keller, A. Sharff, O. Smart, W. Paciorek, T. Womack, G. Bricogne, "Data processing and analysis with the autoPROC toolbox" *Acta Crystallographica Section D: Biological Crystallography* 67 (2011): 293–302, https://doi.org/10.1107/s0907444911007773.

[79] W. Kabsch, "XDS" *Acta Crystallographica Section D: Biological Crystallography* 66 (2010): 125–132, https://doi.org/10.1107/s0907444909047337.

[80] G. M. Sheldrick, "SHELXT – Integrated space-group and crystal-structure determination" *Acta Crystallographica Section A: Foundations and Advances* 71 (2015): 3–8, https://doi.org/10.1107/s2053273314026370.

[81] G. M. Sheldrick, "A short history of SHELX" *Acta Crystallographica Section A: Foundations of Crystallography* 64 (2008): 112–122, https://doi.org/10.1107/s0108767307043930.

[82] L. J. Bourhis, O. V. Dolomanov, R. J. Gildea, J. A. K. Howard, H. Puschmann, "The anatomy of a comprehensive constrained, restrained refinement program for the modern computing environment – Olex2 dissected" *Acta Crystallographica Section A: Foundations and Advances* 71 (2015): 59–75, https://doi.org/10.1107/s2053273314022207.

[83] O. V. Dolomanov, L. J. Bourhis, R. J. Gildea, J. A. K. Howard, H. Puschmann, "OLEX2: a complete structure solution, refinement and analysis program" *Journal of Applied Crystallography* 42 (2009): 339–341, https://doi.org/10.1107/s0021889808042726.

[84] F. Neese, "Software update: The ORCA program system—Version 5.0" *Wiley Interdisciplinary Reviews: Computational Molecular Science* 12 (2022): e1606, https://doi.org/10.1002/wcms.1606.

[85] Chemcraft - graphical software for visualization of quantum chemistry computations. Version 1.8, build 682. https://www.chemcraftprog.com

[86] C. Bannwarth, S. Ehlert, S. Grimme, "GFN2-xTB—An Accurate and Broadly Parametrized Self-Consistent Tight-Binding Quantum Chemical Method with Multipole Electrostatics and Density-Dependent Dispersion Contributions" *Journal of Chemical Theory and Computation* 15 (2019): 1652–1671, https://doi.org/10.1021/acs.jctc.8b01176.

[87] S. Ehlert, M. Stahn, S. Spicher, S. Grimme, "Robust and Efficient Implicit Solvation Model for Fast Semiempirical Methods" *Journal of Chemical Theory and Computation* 17 (2021): 4250–4261, https://doi.org/10.1021/acs.jctc.1c00471.

[88] C. Bannwarth, E. Caldeweyher, S. Ehlert, A. Hansen, P. Pracht, J. Seibert, S. Spicher, S. Grimme, "Extended tight-binding quantum chemistry methods" *Wiley Interdisciplinary Reviews: Computational Molecular Science* 11 (2021): e1493, https://doi.org/10.1002/wcms.1493.

[89] B. de Souza, "GOAT: A Global Optimization Algorithm for Molecules and Atomic Clusters" *Angewandte Chemie International Edition* 64 (2025): e202500393, https://doi.org/10.1002/anie.202500393.
